# Sera from people with HIV and depression induce commensurate metabolic alterations in astrocytes: toward precision diagnoses and therapies

**DOI:** 10.1515/nipt-2024-0001

**Published:** 2024-03-27

**Authors:** Anna Elizabeth Laird, Alexandra Anh Le, Jacqueline R. Kulbe, Anya Umlauf, Melody Sagarian, Matthew Spencer, Anish Sathe, David J. Grelotti, Jennifer Iudicello, Brook Henry, Ronald J. Ellis, Jerel Adam Fields

**Affiliations:** Department of Psychiatry, University of California, San Diego, CA, USA; Department of Neurosciences, University of California, San Diego, CA, USA

**Keywords:** depression, neurocognitive impairment, neuroinflammation, metabolism, astrocytes, HIV

## Abstract

**Objectives:**

People with HIV (PWH) have high rates of depression and neurocognitive impairment (NCI) despite viral suppression on antiretroviral therapy (ART). Mounting evidence suggests that immunometabolic disruptions may contribute to these conditions in some PWH. We hypothesized that metabolic dysfunction in astrocytes is associated with depressive symptoms and cognitive function in PWH.

**Methods:**

Human astrocytes were exposed to sera from PWH (*n*=40) with varying degrees of depressive symptomatology and cognitive function. MitoTracker^TM^ Deep Red FM (MT) was used to visualize mitochondrial activity and glial fibrillary acidic protein (GFAP) as an indicator of astrocyte reactivity using the high-throughput fluorescent microscopy and image analyses platform, CellInsight CX5 (CX5). The Seahorse platform was used to assess glycolytic and mitochondrial metabolism.

**Results:**

More severe depression, as indexed by higher Beck's Depression Inventory (BDI-II) scores, was associated with lower MT signal measures. Better cognitive function, as assessed by neuropsychiatric testing t-scores, was associated with increased MT signal measures. GFAP intensity negatively correlated with several cognitive t-scores. Age positively correlated with (higher) MT signal measures and GFAP intensity. Worse depressive symptoms (higher BDI-II scores) were associated with decreased oxygen consumption rate and spare respiratory capacity, concomitant with increased extracellular acidification rate in astrocytes.

**Conclusions:**

These findings show that factors in the sera of PWH alter mitochondrial activity in cultured human astrocytes, suggesting that mechanisms that alter mitochondrial and astrocyte homeostasis can be detected peripherally. Thus, *in vitro* cultures may provide a model to identify neuropathogenic mechanisms of depression or neurocognitive impairment in PWH and test personalized therapeutics for neurologic and psychiatric disorders.

## Introduction

People with human immunodeficiency virus (HIV; PWH) experience significant comorbidities, including psychiatric disorders (ex: depression) and neurological disorders (ex: HIV-associated neurocognitive disorders (HAND)) despite effective antiretroviral therapies (ART) [1–5]. Depression occurs in PWH at a rate of 2–3× the general population, with some meta-analyses finding that a global average of 39 % of PWH experience depression [6]. Additionally, PWH are living longer as a result of treatment with ART, leading to an increase in the prevalence of HIV-associated neurologic disorders [7]. Low levels of central nervous system (CNS) viral expression and antiretroviral drugs themselves can lead to chronic neuroinflammation and mitochondrial dysfunction [3], pathologies known to occur in a variety of neurologic and psychiatric disorders [8–10]. However, the mechanisms underlying HIV and ART-induced psychiatric and neurologic dysfunction are incompletely understood.

Astrogliosis is a well characterized feature of HIV-associated neuropathology [8, 9, 11] and alterations in glial fibrillary acidic protein (GFAP), a marker of astrocyte reactivity, are associated with a variety of other brain disorders, including major depressive disorder (MDD) [12–17]. Astrocytes play a critical role in maintaining brain homeostasis through forming the blood brain barrier (BBB), providing metabolic support to neurons, responding to inflammatory stimuli, pruning synapses, and regulating neurotransmitters [18–21]. As integral components of the BBB, astrocytes are the first brain cells in contact with the peripheral blood supply, exposing them to both HIV migration into the brain and peripheral factors in the blood, including inflammatory metabolites and cytokines, which may mediate disorders such as depression [22, 23]. Moreover, proteins associated with astrocyte reactivity, such as GFAP, can be detected peripherally. In fact, increases in GFAP have been detected in the blood of people with neurologic or psychiatric disorders [13, 15], including PWH with depression [14]. One way in which astrocytes regulate neuron homeostasis is through alterations in their own metabolism [18, 24, 25]. For example, astrocytes shift from glycolysis to oxidative phosphorylation upon immune stimulation, including exposure to HIV, a metabolic switch that leads to reductions in neuronal mitochondrial activity, but is blocked by inhibiting metabolic activity and immune activation of astrocytes [26–31]. Thus, astrocytes may provide a mechanistic link, through immune reactivity and/or alterations in metabolic activity, between systemic stimuli (whether peripherally or centrally derived) and the neuronal dysfunction that occurs in psychiatric or neurologic disorders.

Therapies targeting HIV-associated neurologic disorders, such as HAND, are lacking, and inflammatory depression, the phenotype that potentially underlies HIV-associated depression, is less responsive to antidepressants [32]. Thus, new approaches are needed to identify mechanisms of and treatments for HIV- and ART-associated psychiatric and neurologic disorders. A recent study showed that sera collected from bipolar patients, compared to sera from controls, induced dendritic simplification in neurons in culture [33], demonstrating that *in vitro* cultures exposed to blood specimens from patients suffering from a psychiatric disorder might reflect and provide a platform to investigate neuropathogenic mechanisms occurring in patients. However, the degree to which sera-induced dendritic simplification varied among the donor samples suggests individualized neuropathogenic mechanisms. Therefore, it is possible that blood samples may be used to identify specific mechanisms underlying disease in individual patients and lead to patient-specific therapeutic approaches.

In this study, we sought to determine if *in vitro* exposure of astrocytes to sera collected from PWH could induce alterations in astrocyte reactivity and mitochondrial activity and if such changes correlate with depressive symptoms or cognitive function. Sera from PWH likely contains peripherally derived inflammatory and hormonal factors as well as brain-derived biomarkers of psychiatric or neurologic disorders. To our knowledge, this is the first study that combines sera samples from PWH with *in vitro* brain cell models to investigate mechanisms underlying depression and cognitive function in this population. The findings here suggest that combining human sera with brain cells may provide valuable insights into personalized mechanisms leading to psychiatric and neurological complications in PWH.

## Methods and materials

### Study population

The inclusion criteria for this study were HIV individuals on ART with viral suppression. Sera from a total of 40 participants with HIV from the HIV Neurobehavioral Research Program [Institutional Review Board (IRB) #080323] were tested, 20 of which were from PWH who had lifetime or current major depressive disorder (MDD) diagnoses and 20 from PWH who did not have MDD diagnoses. Study participants underwent complete neuromedical evaluation, and routine clinical measures were assessed in blood. All studies adhered to the ethical guidelines of the National Institutes of Health and the University of California, San Diego.

### Psychiatric assessments

Participants were evaluated for lifetime (any point in one’s lifetime) and current (last 30 days) MDD and substance use disorder (dependence or abuse) diagnoses using the Composite International Diagnostic Interview (CIDI) [34], a computerized psychodiagnostics clinical interview based on the Diagnostic and Statistical Manual of Mental Disorders, Fourth Edition (DSM-IV), as study methodology was developed prior to the release of the DSM-5. In addition to the diagnostic evaluation, participants completed the Beck Depression Inventory-II (BDI-II) [34], to measure depressive symptoms experienced in the past 2 weeks. The possible range of the BDI-II score is from 0 to 63, with a total score of 0–13 considered no to minimal depressive symptoms, 14–19 mild, 20–28 moderate, and 29–63 severe. In order to preserve statistical power in primary analyses, we chose to model current depression with the total BDI-II score instead of MDD diagnosis, as only 10 total study participants had a current MDD diagnosis. Domain-specific BDI-II scores reflecting cognitive (possible range: 0–27), affective (possible range: 0–12), and somatic (possible range: 0–24) symptoms of depression were computed based on a previous factor analysis of the BDI-II in 1583 PWH [34].

### Neuropsychological assessments

Neuropsychological evaluation was performed as previously described [34–36]. Briefly, seven neurocognitive domains were assessed, including executive function, motor skill, processing speed, episodic memory, attention/working memory, language, and visual perception. Raw test scores were transformed into normally distributed T-scores that were adjusted for demographic variables, including age, education, gender, and race, based on normative samples of HIV participants and were then averaged across all tests to obtain a global cognitive T-score and within domains to obtain cognitive domain-specific T-scores.

### Neuromedical assessment

Baseline demographic data such as age and sex were collected. Medical comorbidities and medications were determined by interview. Additional HIV disease-related variables were collected. These included a history of Acquired Immunodeficiency Syndrome (AIDS), estimated duration of HIV disease (years), current CD4+ T cell count, nadir CD4+ T cell count, and duration of ART use (years). HIV RNA level was measured in plasma by RT-PCR (Abbott Diagnostics; lower limit of quantitation 50 copies/mL).

### Human astrocytes

This study was approved by the University of California, San Diego Human Research Protections Program and deemed IRB exempt (Federal-wide Assurance #00000021 and Institutional Review Board #IORG0000210 [7 March 2019]). All data presented for this study used astrocytes that were from a differentiated cell line originally generated prior to 5 June 2019 (as per NIH NOT-OT-19-128) from fetal human brain tissue from terminated pregnancy between 12 and 16 weeks of gestation, as previously described [37]. Donors gave written informed consent for research use of the cells and tissue. The experiments were repeated and findings were corroborated in SVG p12 astrocyte cultures (ATCC, cat. No. CRL-8261).

### Treatment of astrocytes and high-throughput fluorescent microscopic analyses of astrocytes exposed to patient sera

**Figure 1:**
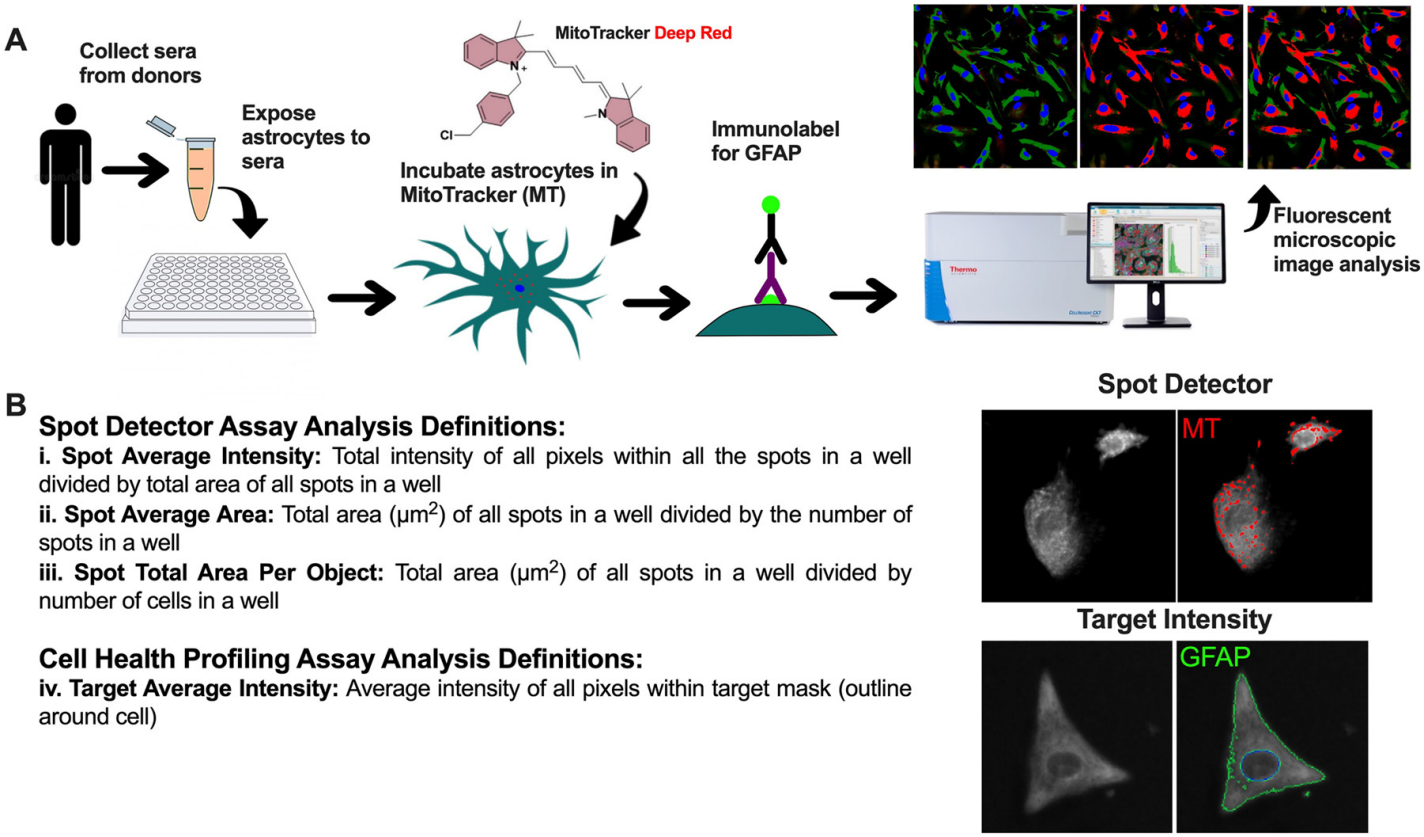
Schematic of experimental design and analysis. (A) Sera was collected from donors (*n*=40). Human astrocytes were exposed to sera for 24 h and then incubated with the potentiometric dye, MitoTracker (MT) and immunolabeled for GFAP (glial fibrillary acidic protein). Astrocytes were then imaged using the high throughput fluorescent microscope image analysis system, Thermo Scientific CellInsight CX5 (CX5). (B) Spot detector assay analysis definitions for spot average intensity, spot average area, and spot total area per object; and cell health profiling assay analysis definitions target average intensity. (C) Representative images for spot detector for MT and target intensity for GFAP.

As depicted in Figure 1A, primary human fetal astrocytes were cultured in one 96-well plate at 5000 cells/well one day prior to treatment. Astrocyte cell cultures were treated in duplicate with sera from 40 PWH. Sera was separated from participant blood using serum separator tubes (BD, cat. No. 367988) and used to treat primary human astrocytes, making up 10 % of the total volume of culture media minus fetal bovine sera (FBS). In parallel on the same plate, astrocytes were treated with culture media containing FBS without interleukin-1β (IL-1β) (negative control) or with IL-1β at 20 ng/mL (positive control, prototypical inflammatory stimuli). Mitochondrial activity was visualized using MitoTracker™ Deep Red (MT) FM (Invitrogen, cat. no. M22426). After incubating with sera for 24 h at 37 °C, astrocyte cultures were incubated with MT at 250 nM for 45 min followed by two washes with 1X PBS and fixation in 4 % paraformaldehyde (PFA) at 4 °C for 20 min. Fixed astrocytes were incubated in blocking buffer (5 % BSA and 0.2 % Triton X-100 in PBS) for 1 h at room temperature before incubating overnight at 4 °C with primary antibody, GFAP 1:500 (Sigma-Aldrich; cat. no. G3893). After three washes with PBS, astrocytes were incubated in secondary antibody, Alexa Fluor Goat anti-Mouse 488 1:500 (Invitrogen, cat. No. A11001), for 30 min. The cells were then stained with blue fluorescent stain, DAPI, 1:10,000, for 5 min and then washed in PBS three times, and imaged and analyzed using Thermo Scientific CellInsight CX5 (CX5) imaging platform (Figure 1B). Images using three channels were captured for four fields of view per well, and this imaging was repeated three more times using different fields of view per well with every scan. Cell Health Profiling Assay was performed to analyze MT and GFAP. A threshold intensity was selected and applied to all wells to identify these targets. MT signal was assessed using the Spot Detector Assay function on the CX5 Cell Insight platform. Data were analyzed as follows: (1) Spot average intensity=total intensity of all pixels within all spots imaged in a well divided by the total area of all spots imaged in a well (i.e. average individual mitochondrion intensity per well), (ii) Spot average area=total area (μm^2^) of all spots imaged in a well divided by the number of spots imaged in a well (i.e. average individual mitochondrion size per well), (iii) Spot total area per object=total area (μm^2^) of all spots in a well divided by the number of cells in a well (i.e. average mitochondrial area per cell per well), and (iv) Target average intensity=average intensity of all pixels within the target mask (outline around cell) (i.e. average intensity of MT or GFAP per cell). This experiment was repeated with SVG p12 (ATCC, cat. No. CRL-8261) cultured in two 96-well plates at 10,000 cells/well (Supplementary data).

### Extracellular flux analyses

To determine if the observed changes in the MT readouts were consistent with metabolic changes in astrocytes, human sera samples with minimal depressive symptoms (BDI<13) and severe depressive symptoms (BDI>25) that fell close to the linear regression line were chosen for further bioenergetic analysis (*n*=5/group). Astrocytes were split into a seahorse plate at 3 × 10^4^ cells/well and exposed to sera (10 % of media volume) for 24 h. Cultures were incubated in a non-CO_2_ incubator at 37 °C to equilibrate for approximately 30 min prior to assay. Baseline measurements of oxygen consumption rate (OCR) and extracellular acidification rate (ECAR) were taken prior to the addition of oligomycin (2 μM), followed by a titrated concentration of FCCP, and then rotenone (500 nM) together with antimycin (1 μM) (Sigma-Aldrich, cat no. A8674). After each addition of mitochondrial inhibitor, three readings were taken before injection of the subsequent inhibitor. Maximum oxygen consumption rate was recorded after two consecutive injections of 250 nM FCCP. Spare respiratory capacity (SRC) was calculated by subtracting the basal OCR from the maximum OCR. ECAR was automatically calculated and recorded by the Seahorse XFe96 software. Rates were calculated by the Seahorse analyzer and reported as pM O_2_/minute and log of H^+^ production rate, respectively. Samples were run in biological replicates of five in three independent experiments.

### Statistical analysis

Statistical analysis was performed using GraphPad Prism 9 and R. For IL-1β treatments and experiments evaluating the effects of sera by BDI-II category (minimal – severe), statistical significance was tested using two-sample *t*-tests or one-way ANOVA with post-hoc Tukey’s pairwise comparisons. Comparisons between MT signal measures and current antidepressant use were done by a two-tailed *t*-test. For the extracellular flux analyses, statistical significance was tested with one-way ANOVA with post-hoc Tukey’s. Simple linear regression was performed to determine the strength of the association between MT signal measures, GFAP intensity, or extracellular flux analyses and clinical measures, including BDI-II scores, cognitive T-scores, or age. R^2^ is used to measure the effect size for each association. MT signal measures and GFAP intensity were normalized to control astrocytes that were treated with media containing FBS. Separate multivariable linear models adjusted for the covariates age, sex, ethnicity, metabolic syndrome, height, duration, and total ART exposure were used to assess the influence of these covariates on the magnitude and significance of the tested associations (Supplementary data). To visually assess how MT and GFAP signals relate to clinical data from sera donors, the data were plotted with the use of a correlation matrix.

## Results

### Clinical characteristics

Demographic and clinical data are illustrated in Table 1. The majority of participants were male. Mean age, duration of ART, and CD4 count do not differ significantly between minimal, mild, moderate, and severe depression groups. Of the 20 individuals with a lifetime diagnosis of MDD, six had a diagnosis of MDD prior to HIV infection. A total of 23 individuals were currently taking antidepressants. One individual had a substance use disorder, specifically cannabis use disorder.

**Table 1:** Clinical characteristics of human cohort.

BDI scores	None/Minimal (*n*=20)	Depressed		
		Mild (*n*=8)	Moderate (*n*=5)	Severe (*n*=7)
**Demographics**				
Sex (f/m)	2/18	1/7	1/4	1/6
Years of age	49.7 ± 12.1	48.4 ± 13.7	47.4 ± 16.0	45.7 ± 6.3
**HIV disease characteristics**				
Nadir CD4 count	188.45 ± 138.96	179.75 ± 130.64	78.6 ± 93.81	274.14 ± 357.07
Current CD4 count	724.2 ± 207.9	657.9 ± 415.2	570.4 ± 313.8	551.8 ± 331.4
Duration of infection (years)	16.96 ± 8.94	16.27 ± 7.2	14.69 ± 10.61	16.64 ± 8.36
Duration on ART regimen (years)	10.4 ± 5.5	11.2 ± 7.9	15.4 ± 15.8	12.3 ± 6.4
AIDS ever (Y/N)	11/7	5/3	5/0	4/3
**Clinical characteristics**				
Current antidepressants	10/20	5/8	2/5	6/7
MDD before/After HIV diagnosis (before/After)	2/7	2/5	0/5	2/3
Current substance use disorder	0/20	0/8	0/5	1/7

### IL-1β induces increased MitoTracker measures and GFAP in cultured primary human astrocytes compared to control and human sera collected from people with HIV and minimal – severe depressive symptoms

**Figure 2:**
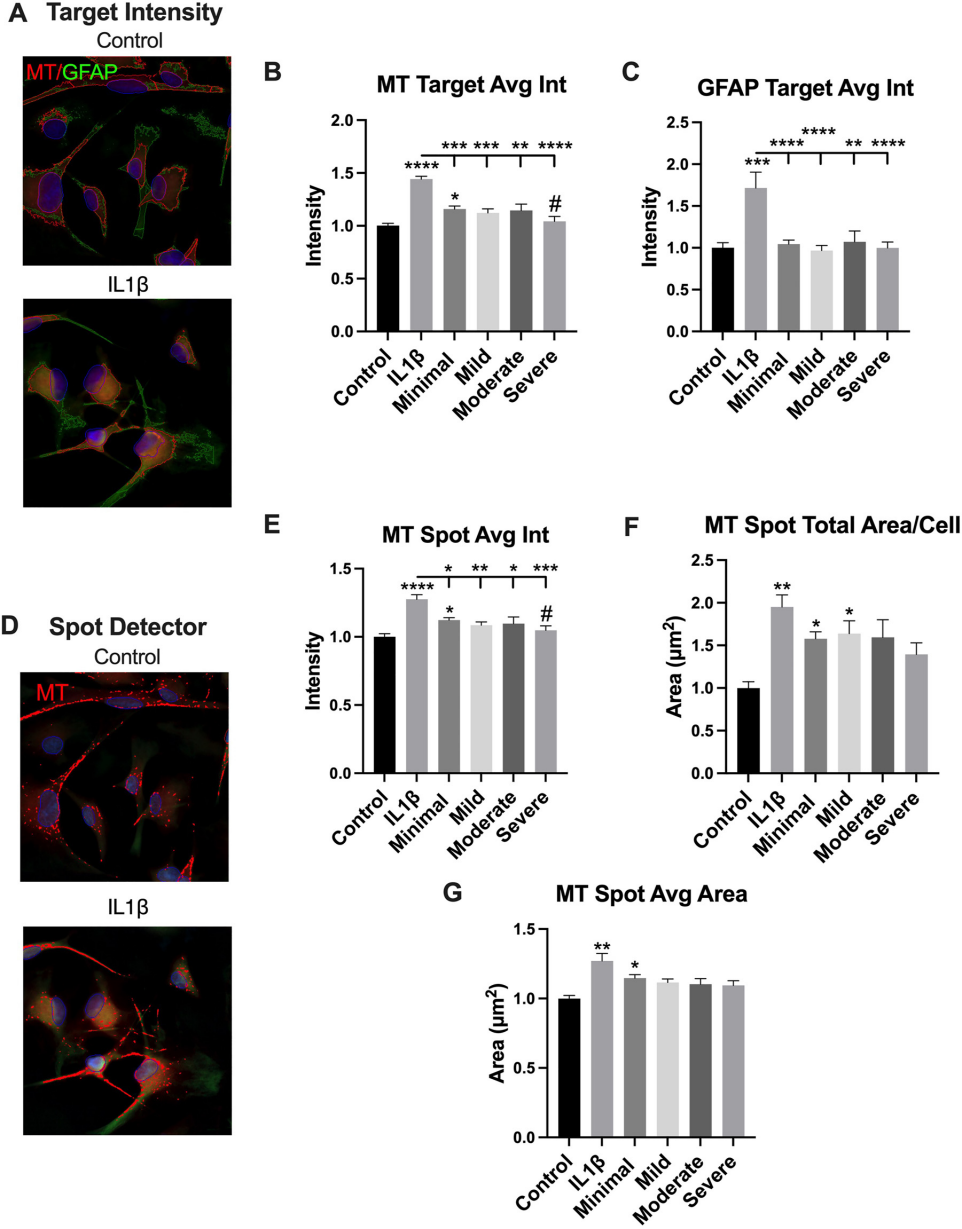
IL-1β induces increased MitoTracker measures and GFAP in cultured primary human astrocytes compared to control and human sera collected from people with HIV and minimal – severe depressive symptoms. (A) Representative images demonstrating target intensity for GFAP and mitotracker (MT) between control (media + FBS) and 20 ng/mL IL-1β (in media + FBS). Scale bar=10 µm. Quantification of target average intensity between control and 24 h of IL-1β treatment or 10 % v/v human sera/media minus FBS for (B) MT and (C) GFAP. (D) Representative images demonstrating spot detector for MT between control and IL-1β. Quantification of (E) MT spot average intensity (F) MT spot total area/cell and (G) MT spot average area between control and 24 h of IL-1β or human sera treatment. Statistical analysis one-way ANOVA with post-hoc Tukey’s *p<0.05, **p<0.01, ***p<0.0001 compared to control or IL-1β (bar). #p<0.05 compared to minimal depressive symptoms via t-tailed *t*-test. *n*=4–6 biologic replicates (Mean ± SEM).

IL-1β induces an 80 and 40 % increase in GFAP and MT target average intensity, respectively, compared to control (media + FBS) (Figure 2A–C). IL-1β significantly increases MT target average intensity compared to human sera collected from PWH with minimal (p<0.001), mild (p<0.001), moderate (p<0.01), or severe depressive symptoms (p<0.0001) (Figure 2B). IL-1β significantly increases GFAP target average intensity compared to human sera collected from PWH with minimal (p<0.0001), mild (p<0.0001), moderate (p<0.01), or severe depressive symptoms (0.0001) (Figure 2C). Sera from PWH with minimal depressive symptoms significantly increases MT target average intensity compared to control (p<0.05) and sera from PWH with severe depressive symptoms (p<0.05) (Figure 2B). IL-1β increases MT spots throughout astrocyte cell bodies and processes (Figure 2D), and quantities of spot total area, average area, and average intensity by 100 , 30, and 35 %, respectively, compared to control (Figure 2E–G). IL-1β significantly increases MT spot average intensity compared to human sera collected from PWH with minimal (p<0.05), mild (p<0.01), moderate (p<0.05), or severe depressive symptoms (p<0.001) (Figure 2E). Sera from PWH with minimal depressive symptoms significantly increase MT spot average intensity compared to control (p<0.05) and sera from PWH with severe depressive symptoms (p<0.05) (Figure 2E). Compared to control, MT spot total area was significantly increased by human sera collected from PWH and minimal (p<0.05) or mild depressive symptoms (p<0.05) (Figure 2F). MT spot average area was significantly increased by human sera from PWH and minimal depressive symptoms (p<0.05) compared to control (Figure 2G).

### Heat map demonstrating relationships between clinical data of donors and astrocyte reactivity and mitochondrial activity in 24 h sera-exposed human astrocyte cultures

**Figure 3:**
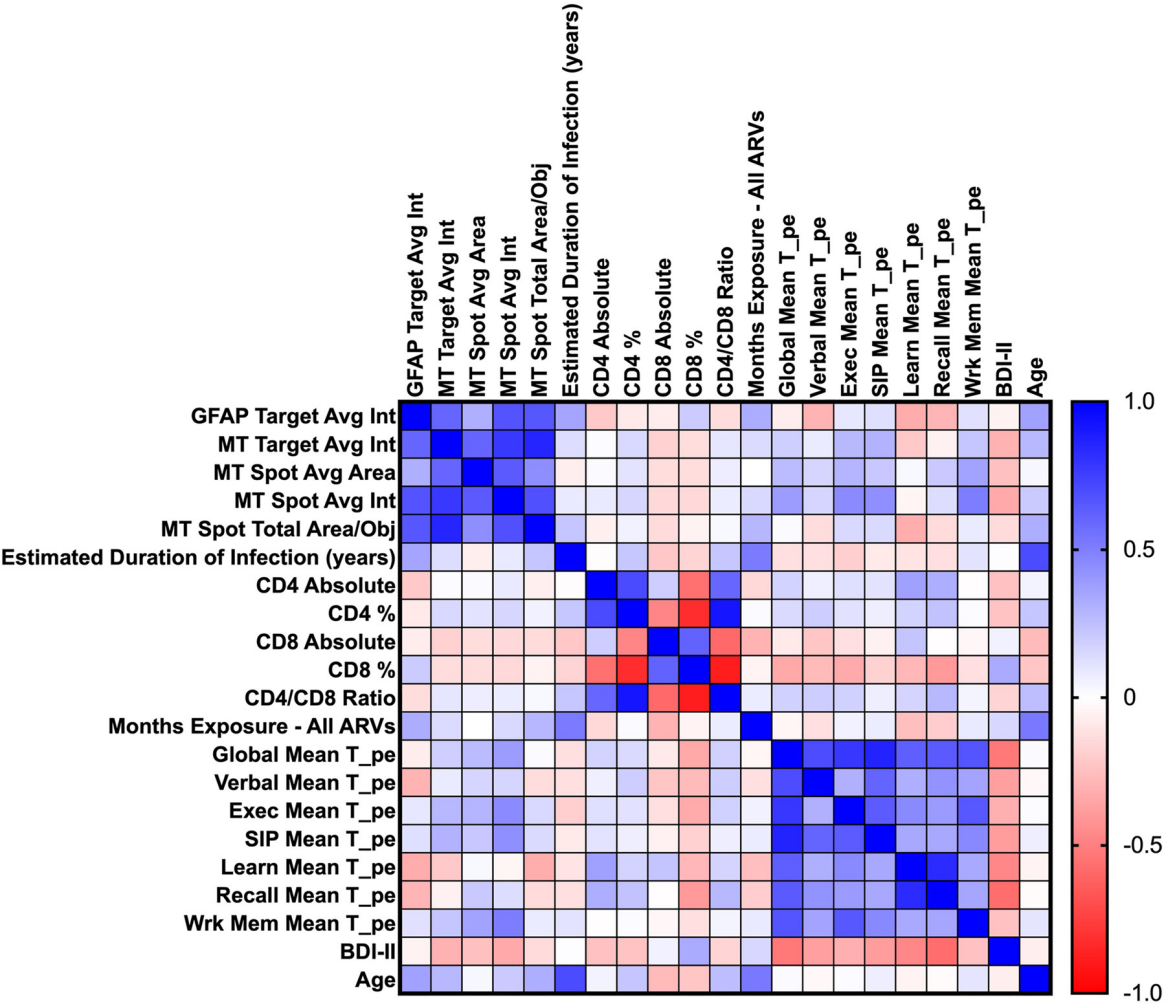
Heat map demonstrating relationships between clinical data of donors and astrocyte reactivity and mitochondrial activity in 24 h sera-exposed human astrocyte cultures. Correlation matrix demonstrating Pearson r correlation coefficients ranging from 1- to −1 and color coded with darker hues representing stronger associations, blue for a positive correlation, white for no correlation, and red for negative correlation. The strongest relationships with GFAP target average intensity occur with age (positive), learning memory (negative), recall memory (negative), and verbal fluency (negative). The strongest relationships with mitotracker (MT) signal measures are with Beck’s depression inventory (BDI-II, negative), executive function (positive), speed of information processing (SIP, positive), and working memory (positive). The matrix also illustrates a strong negative relationship between BDI-II and cognitive T-scores and a strong positive correlation between GFAP target average intensity and MT signal measures.

Correlation matrix analysis (Figure 3) indicates that the strongest relationships with GFAP target average intensity occur with age (positive), learning memory (negative), recall memory (negative), and verbal fluency (negative). The strongest relationships with MT signal measures are with BDI-II (negative), executive function (positive), speed of Information processing (SIP) (positive), and working memory (positive). The matrix also illustrates a strong negative relationship between BDI-II and cognitive T-scores and a strong positive correlation between GFAP target average intensity and MT signal measures.

### A higher burden of depression symptoms was associated with decreased mitochondria activity in sera-exposed astrocyte cultures

**Figure 4:**
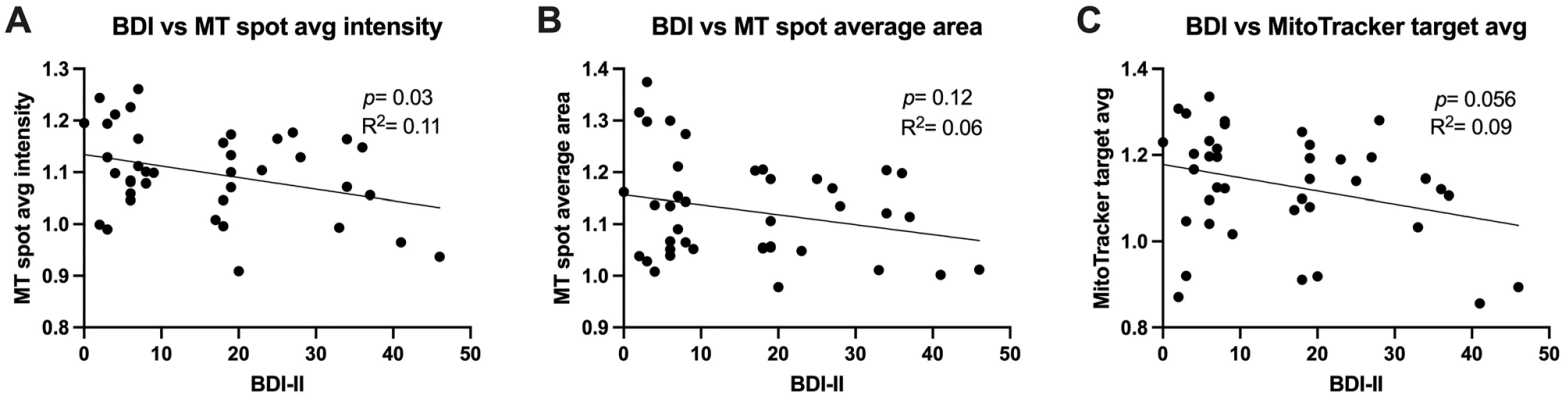
A higher burden of depression symptoms is associated with decreased mitochondrial activity in 24 h sera-exposed human astrocyte cultures. Simple linear regression was conducted to analyze the strength of association between BDI (Beck’s depression inventory) and (A) mitotracker (MT) spot average intensity (B) MT spot average area and (C) MT target average intensity. Higher BDI-II scores are representative of more severe depressive symptoms and higher MT signal measures are representative of more active mitochondria.

MT spot average intensity in astrocyte cultures decreased with increasing BDI-II score (Figure 4A; p=0.03; R^2^=0.11). MT spot average area in astrocyte cultures decreased with increasing BDI-II score (Figure 4B; p=0.12; R^2^ =0.06). MT target average intensity in astrocyte cultures decreased with increasing BDI-II score (Figure 4C; p =0.056; R^2^ =0.09).

### Higher cognitive function T-scores, except for learning T-score, were associated with increased mitochondrial activity in sera-exposed astrocyte cultures

**Figure 5:**
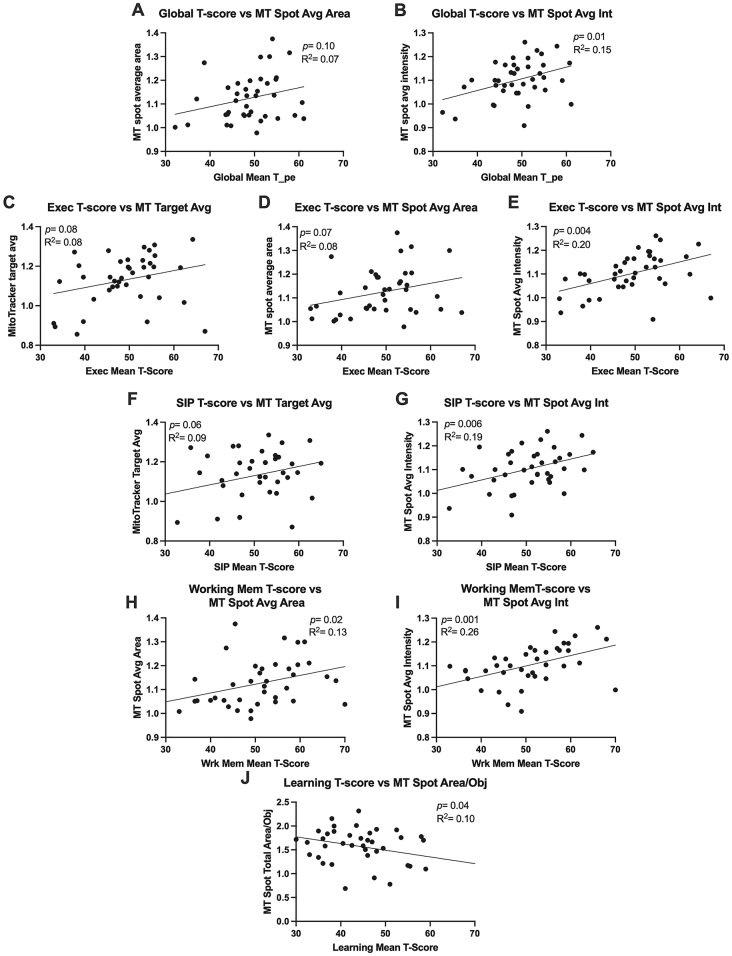
Higher cognitive function T-Scores, except for learning T-score, were associated with increased mitochondrial activity in 24 h sera-exposed human astrocyte cultures. Simple linear regression was conducted to analyze the strength of association between (A) global T-score and mitotracker (MT) spot average area (B) global T-score and MT spot average intensity (C) executive T-score and MT target average intensity (D) executive T-score and MT spot average area (E) executive T-score and MT spot average intensity (F) speed of information processing (SIP) T-score and MT target average intensity (G) SIP T-score and MT spot average intensity (H) working memory T-score and MT spot average intensity (I) working memory T-score and MT spot average intensity (J) learning T-score and MT spot total area/object. Higher T-scores are representative of better performance and higher MT signal measures are representative of more active mitochondria.

MT spot average area (Figure 5A; p =0.10; R^2^ =0.07) and intensity (Figure 5B; p =0.01; R^2^ =0.15) in astrocyte cultures increased with increasing global T-score. MT target average intensity (Figure 5C; p =0.08; R^2^ =0.08), spot average area (Figure 5D; p =0.07; R^2^ =0.08), and spot average intensity (Figure 5E; p =0.004; R^2^ =0.20) in astrocyte cultures increased with increasing executive T-score. MT target average intensity (Figure 5F; p =0.06; R^2^ =0.09) and MT spot average intensity (Figure 5G; p =0.006; R^2^ =0.19) in astrocyte cultures increased with SIP T-score. MT spot average area (Figure 5H; p =0.02; R^2^ =0.13) and MT spot average intensity (Figure 5I; p =0.001; R^2^ =0.26) in astrocyte cultures increased with working memory T-scores. MT spot total area/obj (Figure 5J; p =0.04; R^2^ =0.10) in astrocyte cultures decreased with learning memory T-scores.

### Higher cognitive function T-scores were associated with decreased astrocyte reactivity in sera-exposed astrocyte cultures

**Figure 6:**
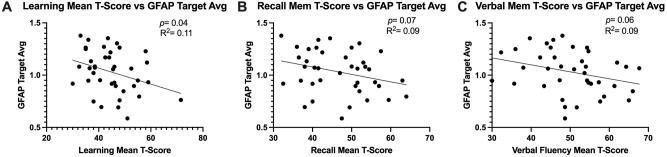
Higher cognitive function T-Scores were associated with decreased astrocyte reactivity in 24 h sera-exposed human astrocyte cultures. Simple linear regression was conducted to analyze the strength of association between GFAP target average intensity and (A) learning mean T-score (B) recall memory T-score (C) verbal fluency T-score. Higher T-scores are representative of better performance and higher GFAP signal intensity is representative of increases in astrocyte reactivity.

GFAP target Average Intensity in astrocyte cultures decreased with increasing learning mean T-score (Figure 6A; p =0.04; R^2^ =0.11), recall memory T-score (Figure 6B; p =0.07; R^2^ =0.09) and verbal fluency T-score (Figure 6C; p =0.06; R^2^ =0.09).

### Increased age is associated with increased mitochondrial activity and astrocyte reactivity in sera-exposed astrocyte cultures

**Figure 7:**
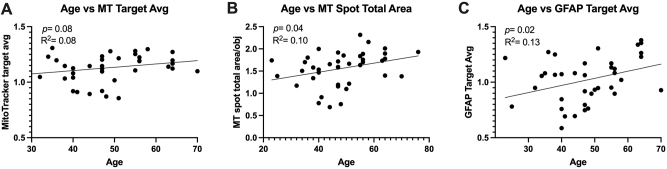
Increased age is associated with increased mitochondrial activity and astrocyte reactivity in 24 h sera-exposed astrocyte cultures. Simple linear regression was conducted to analyze the strength of association between age and (A) mitotracker (MT) target average intensity (B) MT spot total area/obj (C) GFAP target average intensity. Age represented in years. Higher MT signal measures represent more active mitochondria and higher GFAP signal intensity represents increased astrocyte reactivity.

MT target average intensity (Figure 7A; p =0.06; R^2^ =0.08) and MT spot total area/obj (Figure 7B; p =0.04; R^2^ =0.10) in astrocyte cultures increased with increasing age. GFAP target average intensity (Figure 7C; p =0.02; R^2^ =0.13) in astrocyte cultures increased with increasing age. Multivariable models showed results consistent with the results of univariable analyses (Supplementary Material).

### Increased depressive symptoms were associated with decreased oxidative phosphorylation and increased glycolytic activity in sera-exposed astrocytes

**Figure 8:**
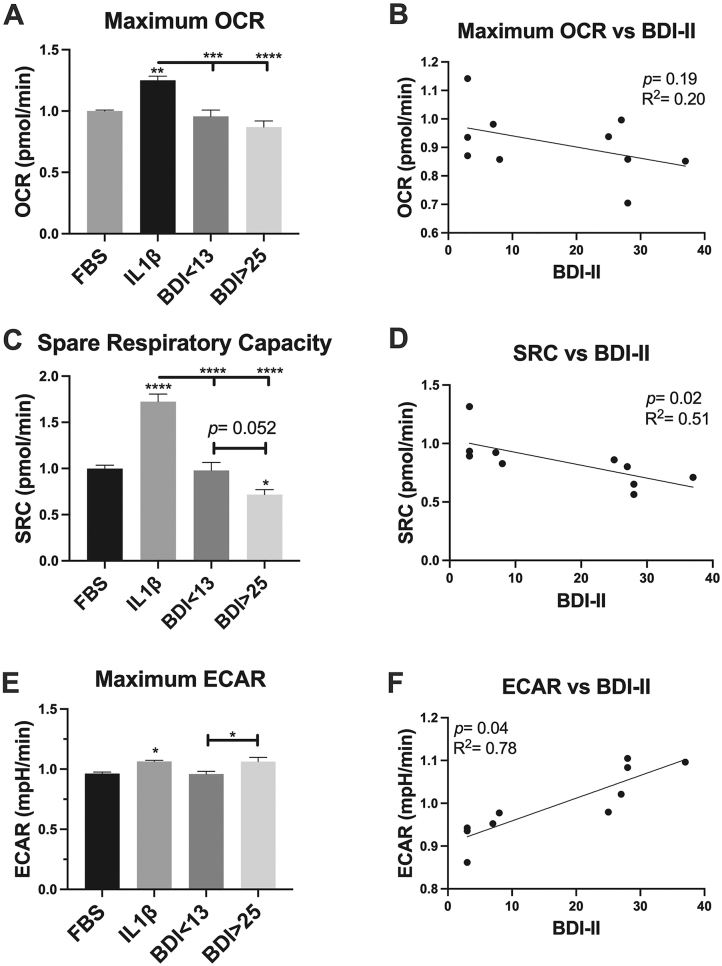
Increased depressive symptoms are associated with reduced oxidative phosphorylation (OCR, SRC) and increased glycolysis (ECAR) in 24 h sera-exposed astrocyte cultures. (A, C, F) FBS=control (media + FBS), IL-1β=20 ng/mL IL-1β in media + FBS, BDI=human sera 10 % v/v in media without FBS. *n*=5/group. *p<0.05, ****p<0.0001 compared to FBS, IL-1β (bar), or BDI=<13 (bar). One-way ANOVA, post-hoc Tukey’s (Mean ± SEM). (B, E, F) Simple linear regression. OCR (oxygen consumption rate), SRC (spare respiratory capacity), ECAR (extracellular acidification rate) were assessed using a Seahorse extracellular Flux Analyzer. Data were normalized to cell count in each well.

OCR and SRC are measures of oxidative phosphorylation. ECAR is a measure of glycolytic activity. Sera from PWH with BDI>25 induced a 10 % reduction in OCR (Figure 8A), a 35 % reduction in SRC (Figure 8C; p=0.052), and a 10 % increase in ECAR (Figure 8E; p <0.05) in astrocytes compared to sera from PWH with BDI<13. IL-1β significantly increases OCR (Figure 8A, p<0.01), SRC (Figure 8C, p<0.0001), and ECAR (Figure 8E, p<0.05) compared to control (media + FBS). There is a non-significant negative correlation between OCR and BDI-II (Figure 8B, p=0.19, R^2^=0.20), i.e. OCR decreases with more severe depressive symptoms. There is a significant negative association between SRC and BDI-II (Figure 8D, p=0.02, R^2^=0.51), i.e. SCR decreases with more severe depressive symptoms. There is a significant positive correlation between ECAR and BDI-II (Figure 8F, p=0.04, R^2^=0.78), i.e. ECAR increases with more severe depressive symptoms.

### Antidepressant medication use was not related to alterations in MT signal in sera-exposed astrocyte cultures

**Figure 9:**
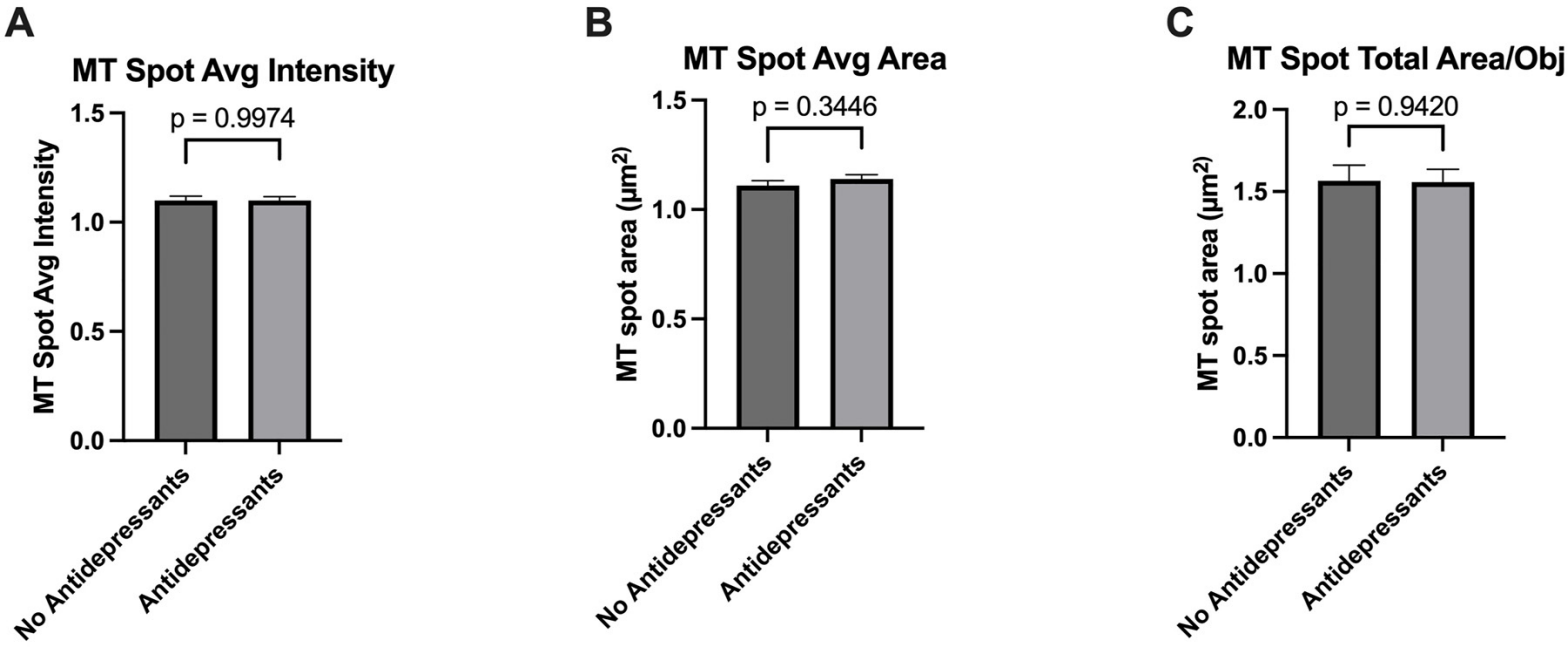
Antidepressant medication usage had no significant change in mitochondrial activity in 24 h sera-exposed astrocyte cultures. Quantification of (A) MT spot average intensity, (B) MT spot average area, and (C) MT spot total area/obj between antidepressant medication users and nonusers. Statistical analysis was performed using a two-tailed *t*-test. *n*=17–23/group(Mean ± SEM).

MT Spot Average Intensity (Figure 9A; p =0.9974), MT Spot Average Area (Figure 9B; p =0.3346), and MT Spot Total Area/Obj (Figure 9C; p =0.9420) in cultured astrocytes had no significant difference between treatment with sera from antidepressant medication users and nonusers.

## Discussion

This is the first study to test the effects of sera from PWH with varying degrees of depressive symptoms and cognitive function on mitochondrial activity and GFAP expression in cultured human astrocytes. These experiments were designed with the hypothesis that peripherally circulating factors in the sera of PWH would induce changes to astrocyte metabolism *in vitro*. Overall, the data confirms our hypothesis and shows that sera from PWH with worse depressive symptoms (as measured by BDI-II scores) leads to decreases in mitochondrial activity, decreases in oxidative phosphorylation, and increases in glycolytic activity in cultured human astrocytes. These alterations in mitochondrial activity are not attributable to antidepressant usage. Additionally, sera from PWH with higher cognitive T-scores leads to increases in mitochondrial activity and decreases in astrocyte reactivity. Both increases in mitochondrial activity and astrocyte reactivity are associated with older age. The properties of sera from PWH to induce changes in astrocyte reactivity and mitochondrial metabolic activity may either be due to the presence of circulating factors derived directly from the CNS (ex: exosomes, metabolites) or from factors in the periphery that possess the ability to regulate astrocyte homeostasis through a signaling mechanism that may involve the BBB. In either case, such compounds likely occur at lower concentrations in the periphery than in the CNS.

There is mounting evidence for the role of inflammation in depression [7]. Peripheral levels of inflammatory cytokines are elevated in people with MDD [38], and the inflammasome pathway, which leads to IL1β activation, has been implicated both in depression [39] and HIV-associated neurological dysfunction [40, 41]. The inflammatory association is particularly important in PWH because elevated levels of inflammation are associated with depressive symptoms, and chronic inflammation occurs in PWH despite viral suppression with ART [34, 42, 43]. Neuroinflammation is associated with increases in GFAP, reflective of increases in astrocyte reactivity [11, 12, 14, 16]. Reactive astrocytes produce inflammatory cytokines and undergo morphologic changes, leading to increased energy demands. Thus, neuroinflammation is also associated with an increase in astrocyte mitochondrial oxidative phosphorylation [24, 25].

Therefore, we hypothesized that higher depressive symptoms would be associated with increases in both GFAP and mitochondrial activity, as seen with the prototypical inflammatory stimuli IL-1β. Although there was an overall positive correlation between GFAP intensity and mitochondrial activity, the observation that decreases in mitochondrial activity were associated with higher depression symptoms absent changes in GFAP was somewhat unexpected.

Despite our unexpected findings, these results do not exclude a role for inflammation in HIV-associated depression. Chronic levels of neuroinflammation in PWH induce astrocytes to switch from their basal glycolytic state to one of oxidative phosphorylation [26], [27], [28, 44], which has been demonstrated to be toxic to surrounding neurons [29]. Therefore, it is possible that a compensatory mechanism in the sera of PWH with depression is being produced in order to induce astrocytes back to a glycolytic state, which may allow astrocytes to better provide supportive substrates to surrounding neurons [18, 45], [46], [47]. This is consistent with our data indicating that more severe depressive symptoms are associated with higher rates of glycolysis as assessed by ECAR. Alternately, the relationship between inflammation and depressive symptoms cannot be completely explored with the limited outcome measures of mitochondrial activity and GFAP intensity. For example, inflammatory cytokines can alter the reuptake and synthesis of the monoamine neurotransmitters (ex: serotonin, norepinephrine, and dopamine) involved in depression [48–50] and induce monoamine oxidase, a mitochondrial enzyme involved in the metabolism of monoamines [51]. However, these measures were not included as part of this study. It is also possible that although IL-1β is a marker of inflammasome activation, a pathway implicated in depression [39], it is not the ideal model for depressive inflammation. This would be consistent with the current findings that demonstrate differential responses between astrocytes treated with IL-1β and human sera. In fact, a recent study failed to find increases in peripheral IL-1β in individuals with depression [38]. Unfortunately, sera levels of IL-1β or other pro-inflammatory cytokines were unavailable for this cohort. The lack of these measures represents a limitation of this study, and future studies should include these assessments.

Of note, only six individuals in this study carried a diagnosis of MDD prior to HIV infection (Table 1). It is possible that for the individuals whose HIV infection preceded their depression, their depression is due to a virally related pathogenic mechanism. As discussed above, chronic inflammation in PWH, even while virally suppressed with ART, is thought to contribute to depression in PWH [51]. However, due to the small number of individuals with MDD diagnoses preceding HIV infection, this relationship could not be further explored. Future studies investigating the temporal relationship between MDD and HIV infection and their associated immunometabolic processes are needed, as well as studies of MDD in HIV-negative individuals. Overall, this work highlights the complexity of the factors underlying depressive symptoms in PWH and illustrates a platform that could be used to investigate individual pathogenic mechanisms and to identify and probe potential therapeutic targets.

This study also examined the associations between cognitive T-scores and *in vitro* sera-induced changes to mitochondrial activity and GFAP reactivity. Overall, the results demonstrate that higher cognitive T-scores are associated with increases in mitochondrial activity and decreases in astrocyte reactivity. In other words, lower mitochondrial activity is associated with lower cognitive T-scores. Of note, lower mitochondrial activity was also associated with higher depressive symptoms, and higher depressive symptoms were correlated with lower cognitive function. Therefore, it is possible that in this cohort of individuals, lower cognitive function occurs secondary to depressive symptoms rather than being an independent neurodegenerative process. However, this analysis was beyond the scope of this study and should be pursued in future investigations.

The increases in GFAP intensity that occur in the astrocytes treated with sera derived from individuals with lower cognitive T-scores may suggest that an inflammatory stimulus is present in the sera. Previous studies have shown that increases in GFAP intensity coincide with increases in mitochondrial activity [26, 27, 52], an overall correlation that holds true in this study. Therefore, it may appear counterintuitive that in the context of cognition function, lower cognitive T-scores are associated with higher GFAP intensity but lower mitochondrial activity. It is possible that the astrocytes are being directed toward a more glycolytic state in a homeostatic attempt to provide metabolic support for impaired neurons that may be present in individuals with lower cognitive T-scores [29, 31]. Additionally, although astrocytes are often canonically presented as increasing oxidative phosphorylation following an inflammatory stimulus and can be simplistically divided into A1 (inflammatory) and A2 (neuroprotective) phenotypes, the reality is that astrocyte phenotypes are expressed on a more nuanced spectrum [20, 53], [54], [55]. This may be a factor in the current studies in which individual patient sera is being used to treat astrocytes, and likely represents a more complex paradigm than simple IL-1β stimulation.

This study also examined the associations between age and *in vitro* sera-induced changes to mitochondrial activity and GFAP reactivity. Age is associated with elevated levels of inflammation in PWH [8]. Therefore, it is unsurprising that increases in GFAP and mitochondrial activity were associated with older age, reflecting the pattern seen *in vitro* with the inflammatory stimulus IL-1β. This is opposite to what was seen with the cognitive T-score data. It is possible that homeostatic mechanisms capable of inducing a metabolic switch to the more neuronally supportive glycolytic astrocyte phenotype become impaired with age.

Within the statistically significant linear regression models, the R^2^ values ranged from 0.06 to 0.25, implying that additional variables beyond depression, cognitive T-scores, and age likely influence changes to mitochondria and GFAP signal measures. Controlling for covariates did not substantially change the results seen in the simple linear regression (Supplementary data). It is likely that individual variability accounts for the lower R^2^ values, which is not unexpected in clinic samples and consistent with *in vitro* studies demonstrating differential effects of sera from bipolar patients on *in vitro* dendritic sprouting [33]. Pathophysiologic variability amongst people with depression is known to occur. For example, biomarkers of inflammation are elevated in some people with depression but not others, suggesting that inflammation may be only one component of a complex interaction of pathways involved in depressive symptoms. Additionally, the methods used in this study serve as a high throughput screening tool to identify associations between cellular processes and clinical measures. It allows for future studies to validate associations using more in-depth methods such as mitochondrial bioenergetic analyses, and to identify mechanistic differences between individuals who fall along the regression slope versus outlying individuals.

As stated previously, inflammation has been implicated in a variety of psychiatric and neurologic disorders, including depression and HAND [8], [9], [10, 43, 56, 57]. However, clinical trials targeting such mechanisms have shown minimal effectiveness across populations. This has led to increased emphasis on personalized medicine to use genetics to identify therapies best suited for particular individuals. However, while providing powerful insights into disease, genetics does not explain all variability in any given population. Thus, it would be useful to identify tools that can disentangle inflammation-associated depression or cognitive symptoms from depression or cognitive symptoms associated with other neuropathogenic mechanisms. *In vitro* models that expose neuroinflammatory cells such as astroglia to patient sera samples may be useful in both identifying patients with inflammation-associated symptoms of depression or cognitive dysfunction, and for testing therapeutic strategies *in vitro*. However, more detailed mechanistic studies in larger cohorts, run in parallel with peripheral measures of inflammation, such as cytokine analyses, are needed to determine the feasibility of this approach.

This study is limited by several factors. The small size of the cohort and the lack of diversity controls for demographic characteristics between donors are not ideal for such translational studies. Future studies are needed to investigate these findings amongst cohorts of different age, sex, and race. This cohort only included people with HIV, which limits its generalizability to non-HIV populations. Future studies should be repeated in individuals without HIV. The human astrocyte model is generated from fetal tissues, which may confer epigenetic architecture that is not reflective of adult PWH nor their astrocytes. Astrocytes derived from adult postmortem brain tissues or iPSC-derived astrocytes from the serum donor may provide a model with more relevant epigenetic architecture. Although serum consists of all the components that interact with astrocytes at the BBB, not all serum proteins and glycoproteins cross the BBB. Thus, exposing astrocytes to donor CSF may provide a more accurate reflection of the *in vivo* environment. Neurocognitive impairment diagnoses remain high in PWH, and while we did find significant relationships between astrocyte metabolic activity and individual cognitive T-scores, we did not consider relationships with diagnoses of neurocognitive impairment. Future studies are needed to investigate the relationship between patient sera-induced changes in metabolic activity in brain cells and cognitive diagnoses. This study provides associations and implicates possible mechanisms but does not provide orthogonal experiments to definitively test such mechanisms. Further studies are needed to elucidate the molecular mechanisms underlying the effects of sera from PWH and depressive and cognitive symptoms on astrocyte mitochondrial activity and GFAP intensity. More in-depth studies are needed to determine the causal relationship between mitochondrial alterations and astrocyte reactivity in PWH with depressive and cognitive symptoms. Investigating the signaling pathways and cellular processes involved in these changes could provide insights into potential therapeutic targets for managing depressive and cognitive symptoms. Longitudinal studies that track the changes in astrocyte mitochondrial activity and GFAP intensity in response to sera collected over time could provide insights into the progression of depressive and cognitive disorders. This approach could potentially identify patterns of astrocytic and mitochondrial alterations that correlate with disease progression, remission, or treatment response. Exploring the genetic and epigenetic factors that contribute to variations in astrocyte responses to sera from individuals with depressive and cognitive symptoms could help explain the observed diversity in signal changes. Investigating potential gene-environment interactions may provide a more comprehensive understanding of these relationships. If the correlations observed in this study are confirmed in larger and more diverse populations, it could open up possibilities for developing novel therapeutic strategies that target astrocyte activity to alleviate depressive symptoms and enhance cognitive function.

## Conclusions

This is the first study to our knowledge that combines sera samples from PWH with *in vitro* brain cell models to investigate mechanisms underlying depression and cognitive function in PWH. This study provides evidence for the impact of sera from PWH with varying depressive symptoms on mitochondria activity and astrocyte reactivity in human astrocyte cultures. It also establishes relationships between these signals, cognitive factors, and age. It sheds light on potential connections between brain function, depressive symptoms, cognitive function, and astrocyte mitochondrial activity. Specifically, the findings support a role for astrocytes and disrupted mitochondrial homeostasis in depression and cognitive function during HIV infection. The findings support the utility of combining patient specimens with *in vitro* and ex vivo brain cell models to gain insights into brain dysfunction and potentially test personalized therapeutic strategies.

## Highlights

– Depression in people with HIV is associated with reduced mitochondrial oxidative phosphorylation and increased glycolytic activity in astrocytes
– Better cognitive T-scores in people with HIV are associated with increased mitochondrial activity and decreased astrocyte reactivity
– Combining patient sera with human brain cells provides personalized mechanistic insights into neurological disorders

## Supplementary Material

Supplementary Material Details
