# Supplementary material for: Sera from people with HIV and depression induce commensurate metabolic alterations in astrocytes: toward precision diagnoses and therapies
Source: NeuroImmune Pharm Ther. 2024 Mar 27;3(2):113–28. doi: 10.1515/nipt-2024-0001 (PMC11338010; doi:10.1515/nipt-2024-0001)
Supplement: Supplementary file 1 — Supplementary Material Details [file j_nipt-2024-0001_suppl_001.pdf]

Multivariable analyses: mitochondrial reactivity and GFAP expression

Prepared by Anya Umlauf

September 18, 2023

Contents

1 Data Summary 2
1.1 Outcomes . . . 2
1.2 Predictors . . . 4
1.3 Covariates . . . 5

2 Simple associations 6
2.1 Correlation between outcomes & predictors . . . 6
2.2 Simple associations with covariates . . . 12
2.3 Summary of univariable analyses . . . 32
2.3.1 Simple correlations between outcomes and predictors . . . 32
2.3.2 Associations between outcomes and covariates . . . 32
2.3.3 Associations between predictors and covariates . . . 32

3 Multivariable models 33
3.1 Controling for one covariate. . . . 33
3.2 Controling for covariates significant at 0.20 level . . . 39

Note: None of the analyses shown below are corrected for multiple testing.

1 Data Summary

There are data for n=40 participants. The following tables and figures summarize variables used for the analyses. Note that measure units were guessed to be mg/dL for cholesterol and triglycerides.

1.1 Outcomes

Table 1: Summary of the outcomes.

Outcome	Mean (SD)	Median [IQR]	Min-Max	Missing n (%)
BDI-II	15.9 (12.6)	13.0 [6.0, 23.5]	0.0 - 46.0	0 (0%)
BDI-II Cognitive	5.35 (5.46)	4.00 [1.00, 8.25]	0.00 - 18.00	0 (0%)
BDI-II Apathy	3.62 (3.18)	3.00 [1.00, 6.00]	0.00 - 11.00	0 (0%)
BDI-II Somatic	7.47 (5.55)	6.00 [3.75, 11.00]	0.00 - 21.00	0 (0%)
BDI-II Affective	3.08 (2.78)	2.00 [0.75, 5.25]	0.00 - 9.00	0 (0%)
Global Mean T	48.9 (6.51)	49.0 [45.3, 53.2]	32.2 - 61.1	1 (2.5%)
Verbal T	50.2 (9.22)	50.7 [44.7, 55.0]	30.0 - 67.7	1 (2.5%)
Executive T	49.1 (8.30)	49.7 [45.4, 54.4]	33.0 - 67.0	1 (2.5%)
SIP T	50.2 (8.25)	51.8 [46.2, 55.4]	28.5 - 65.0	1 (2.5%)
Learning T	44.8 (8.79)	44.5 [38.0, 48.8]	30.0 - 71.5	1 (2.5%)
Recall T	46.8 (8.37)	47.5 [40.0, 52.5]	32.0 - 64.0	1 (2.5%)
Working Memory T	50.4 (9.69)	51.0 [44.5, 57.2]	26.0 - 70.0	1 (2.5%)
Motor T	48.9 (12.0)	52.0 [43.0, 58.0]	7.0 - 65.5	1 (2.5%)
Total Cholesterol (mg/dL)	187 (43.6)	187 [155, 222]	102 - 286	1 (2.5%)
Triglycerides (mg/dL)	200 (117)	169 [126, 244]	41 - 507	1 (2.5%)

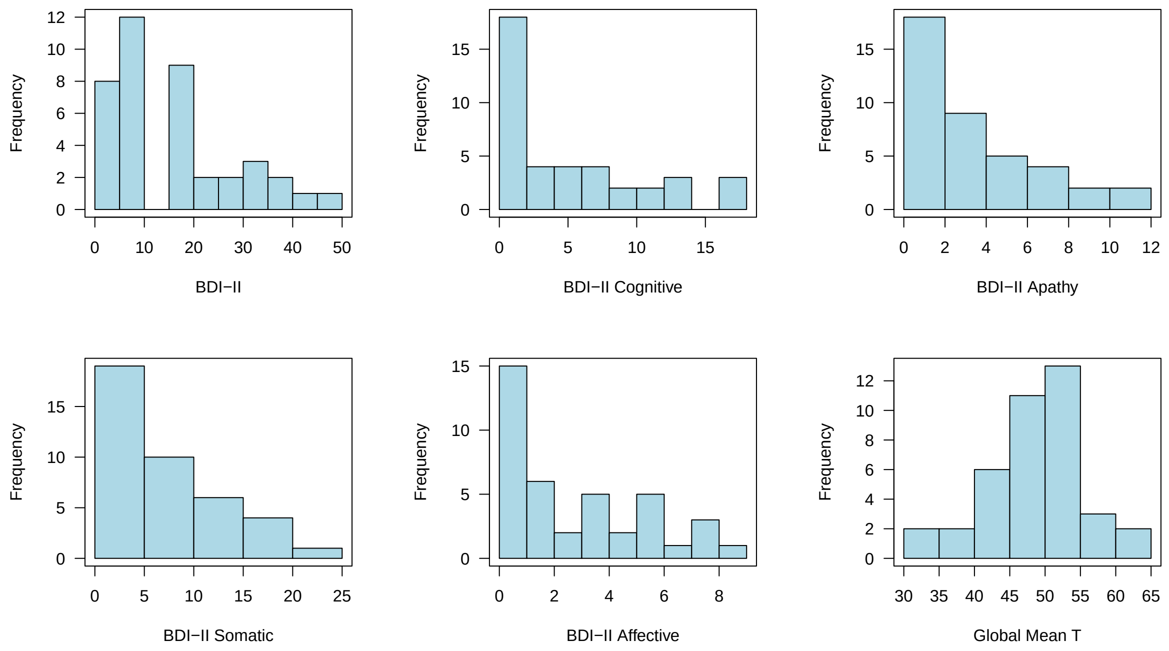

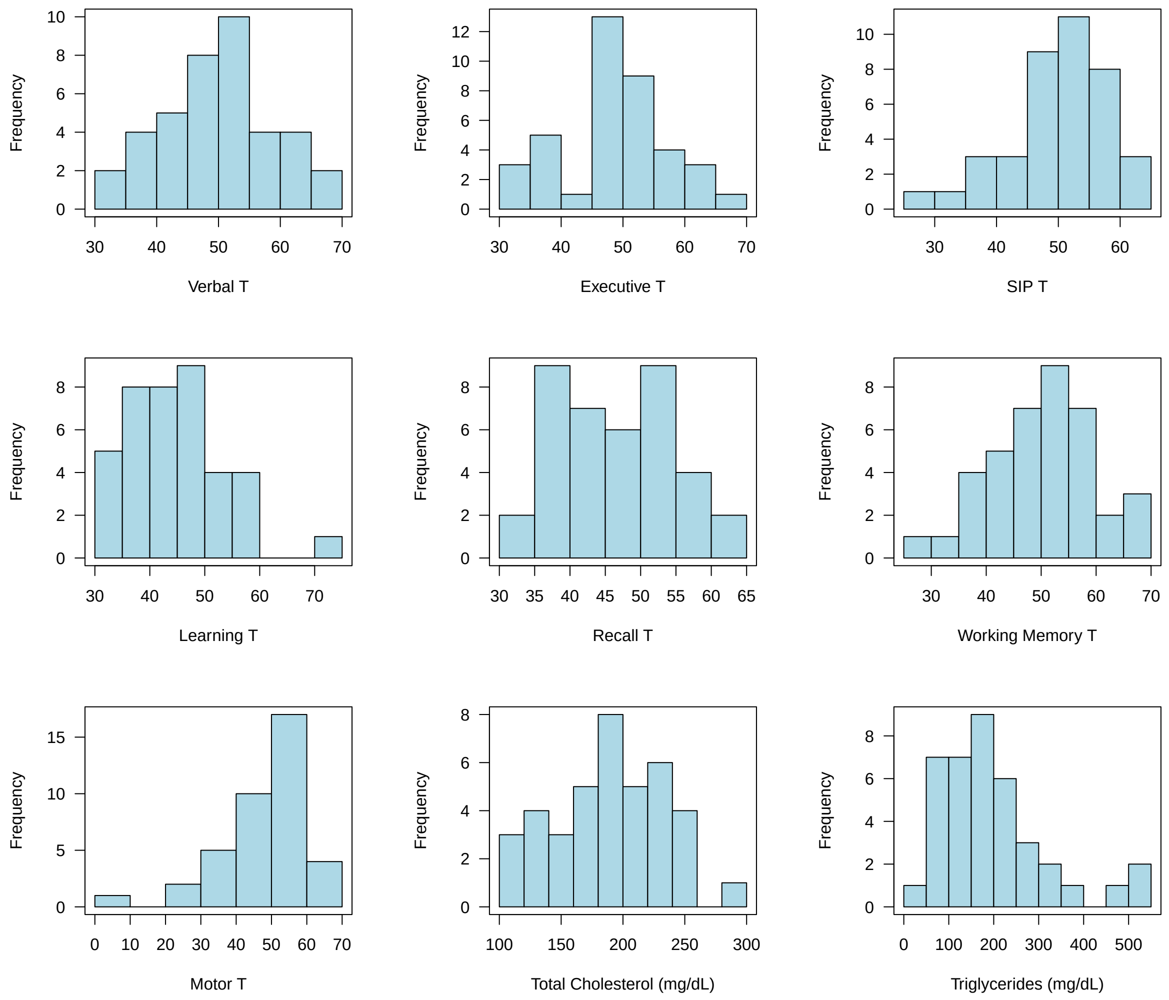
Frequency
Verbal T
Executive T
SIP T
Learning T
Recall T
Working Memory T
Motor T
Total Cholesterol (mg/dL)
Triglycerides (mg/dL)

1.2 Predictors

Table 2: Summary of the predictors.

Predictor	Mean (SD)	Median [IQR]	Min-Max	Missing n (%)
MT spot average area	1.13 (0.098)	1.12 [1.05, 1.19]	0.98 - 1.38	0 (0%)
MT spot average intensity	1.10 (0.084)	1.10 [1.05, 1.16]	0.91 - 1.26	0 (0%)
MT spot total area/object	1.56 (0.380)	1.65 [1.37, 1.81]	0.69 - 2.32	0 (0%)
MT target average	1.13 (0.127)	1.15 [1.07, 1.22]	0.86 - 1.34	0 (0%)
GFAP target average	1.02 (0.206)	1.06 [0.90, 1.18]	0.59 - 1.38	0 (0%)

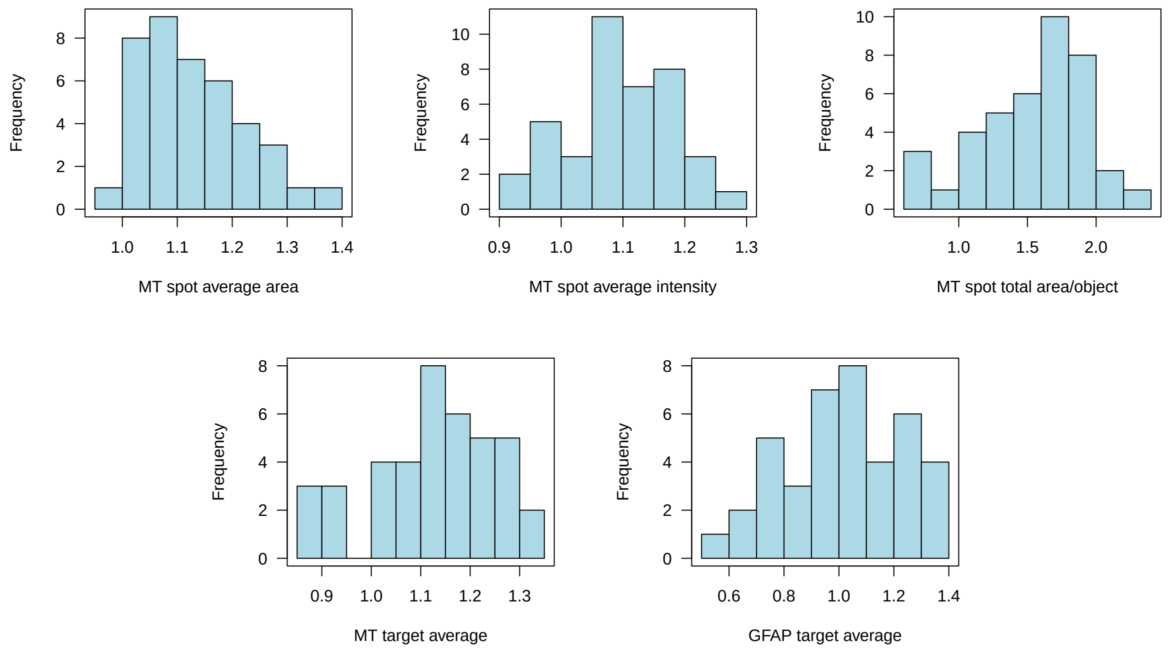

1.3 Covariates

Table 3: Summary of the covariates.

**Covariate**	**Mean (SD) or n (%)**	**Median [IQR]**	**Min-Max**	**Missing n (%)**
Age (years)	48.4 (11.8)	47.0 [40.0, 56.0]	23.0 - 76.0	0 (0%)
Male sex	35 (87.5%)	—	—	0 (0%)
Ethnicity	—	—	—	0 (0%)
– asn	1 (2.5%)	—	—	—
– blk	11 (27.5%)	—	—	—
– his	6 (15%)	—	—	—
– oth	1 (2.5%)	—	—	—
– wht	21 (52.5%)	—	—	—
Metabolic syndrome	17 (42.5%)	—	—	0 (0%)
Duration on ART (months)	138.2 (93.1)	132.4 [87.2, 167.5]	6.4 - 493.6	0 (0%)
Total d'drug exposure (months)	53.3 (125.6)	0.0 [0.0, 32.2]	0.0 - 554.9	0 (0%)
Height (inches)	68.5 (3.8)	69.0 [65.8, 71.2]	59.0 - 75.0	0 (0%)
D'drug exposure	15 (37.5%)	—	—	0 (0%)

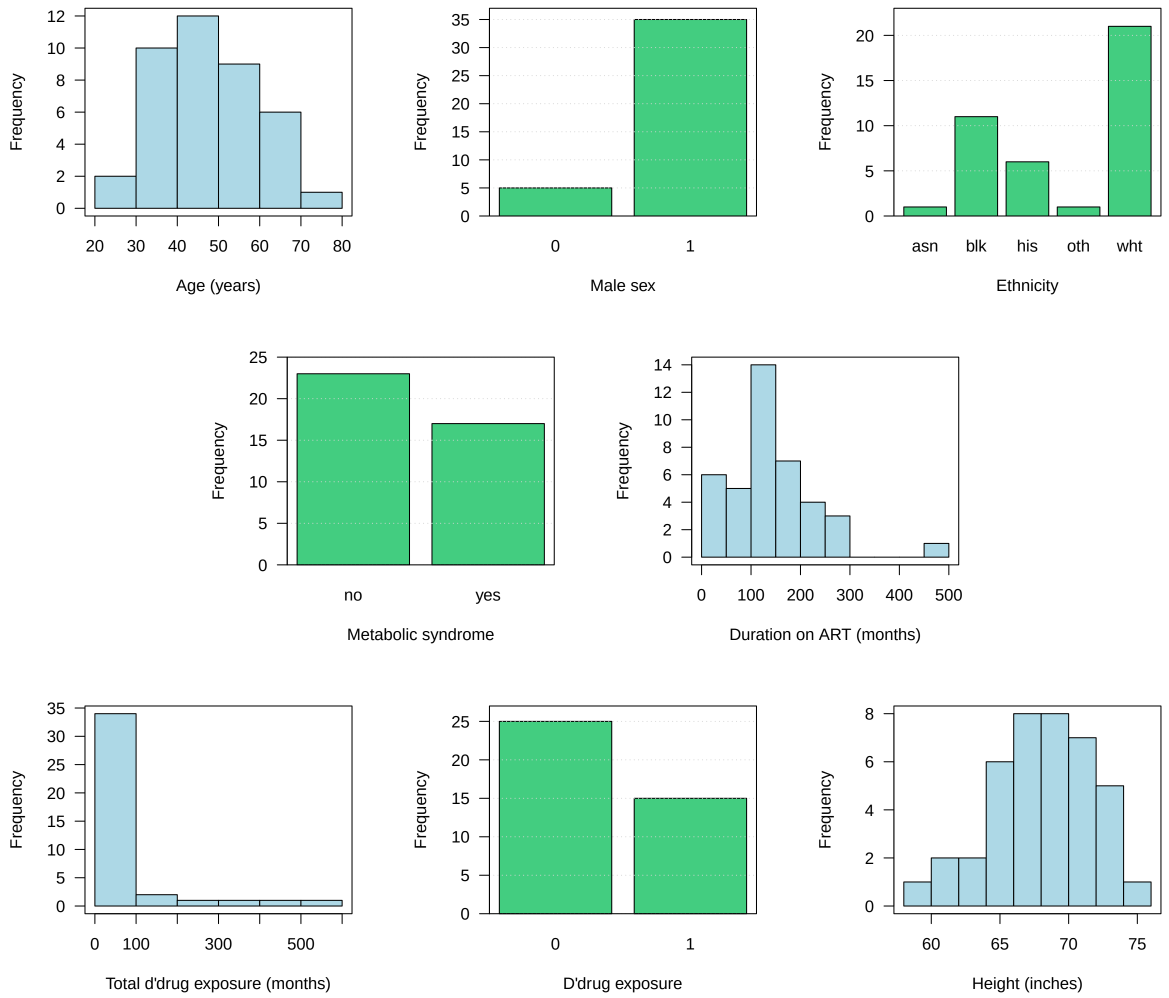

2 Simple associations

2.1 Correlation between outcomes & predictors

The tests shown below are not corrected for multiple testing. The figures are clustered by predictors.

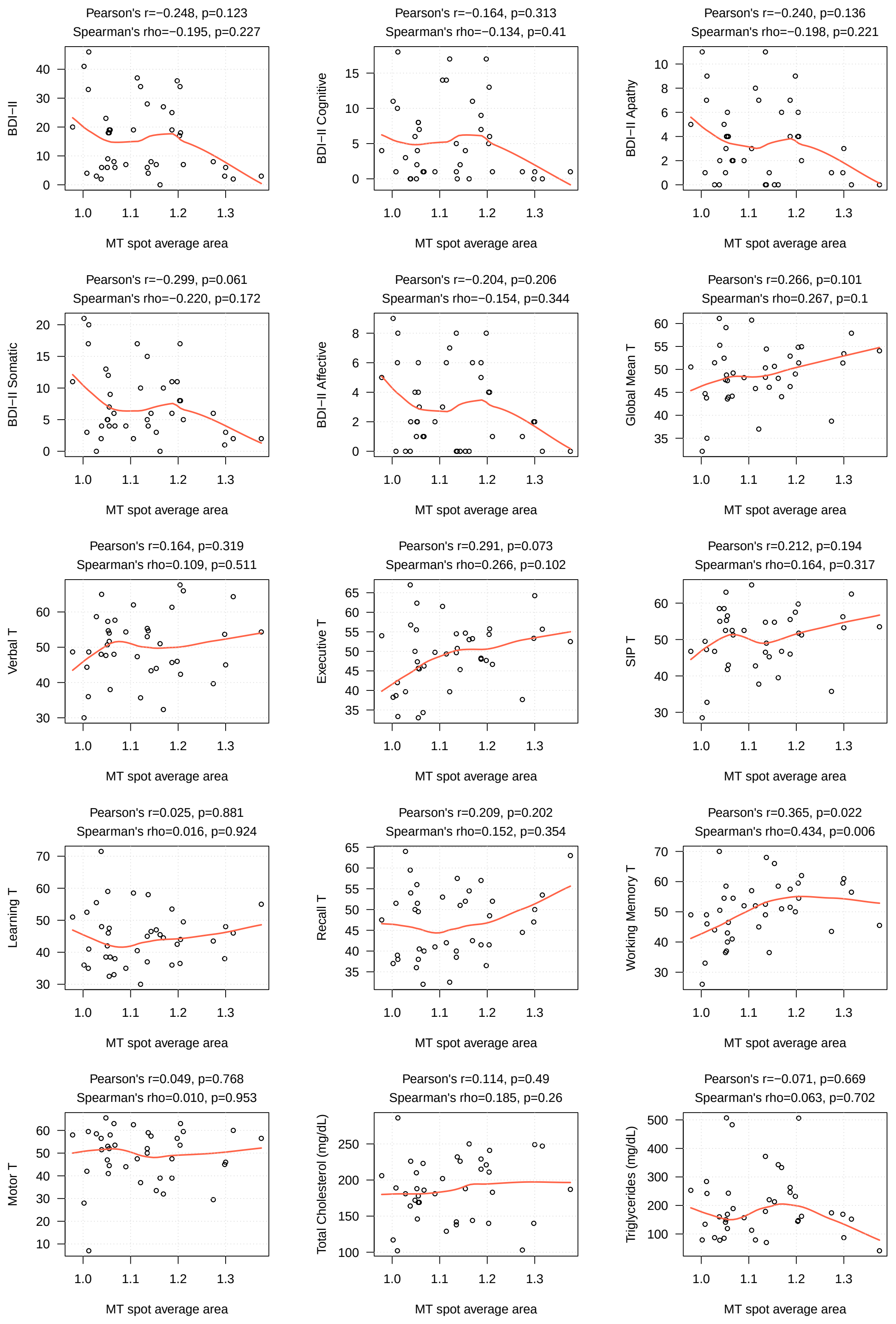

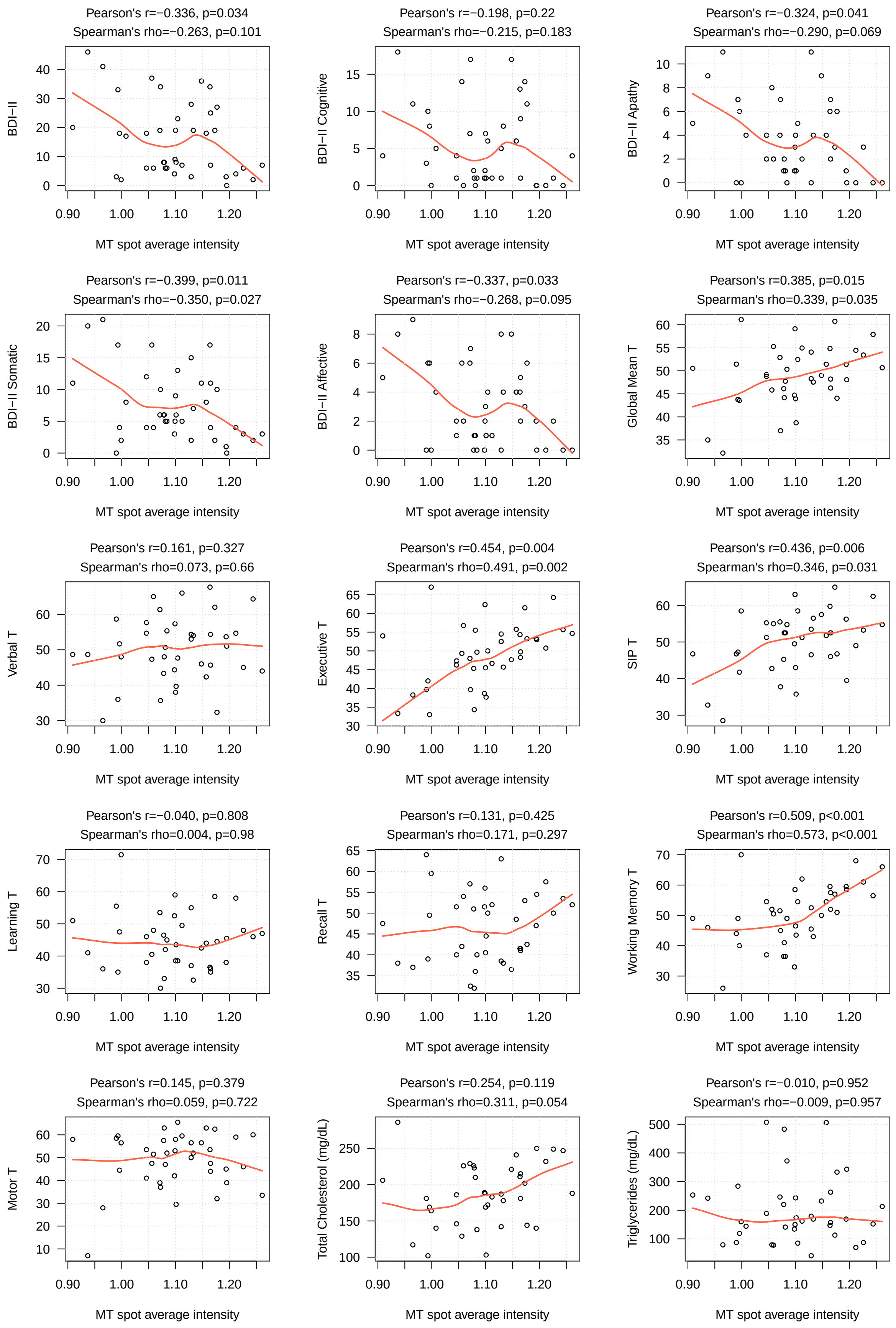
Pearson's r=−0.336, p=0.034
Spearman's rho=−0.263, p=0.101
BDI−II
MT spot average intensity
Pearson's r=−0.198, p=0.22
Spearman's rho=−0.215, p=0.183
BDI−II Cognitive
MT spot average intensity
Pearson's r=−0.324, p=0.041
Spearman's rho=−0.290, p=0.069
BDI−II Apathy
MT spot average intensity
Pearson's r=−0.399, p=0.011
Spearman's rho=−0.350, p=0.027
BDI−II Somatic
MT spot average intensity
Pearson's r=−0.337, p=0.033
Spearman's rho=−0.268, p=0.095
BDI−II Affective
MT spot average intensity
Pearson's r=0.385, p=0.015
Spearman's rho=0.339, p=0.035
Global Mean T
MT spot average intensity
Pearson's r=0.161, p=0.327
Spearman's rho=0.073, p=0.66
Verbal T
MT spot average intensity
Pearson's r=0.454, p=0.004
Spearman's rho=0.491, p=0.002
Executive T
MT spot average intensity
Pearson's r=0.436, p=0.006
Spearman's rho=0.346, p=0.031
SIP T
MT spot average intensity
Pearson's r=−0.040, p=0.808
Spearman's rho=0.004, p=0.98
Learning T
MT spot average intensity
Pearson's r=0.131, p=0.425
Spearman's rho=0.171, p=0.297
Recall T
MT spot average intensity
Pearson's r=0.509, p<0.001
Spearman's rho=0.573, p<0.001
Working Memory T
MT spot average intensity
Pearson's r=0.145, p=0.379
Spearman's rho=0.059, p=0.722
Motor T
MT spot average intensity
Pearson's r=0.254, p=0.119
Spearman's rho=0.311, p=0.054
Total Cholesterol (mg/dL)
MT spot average intensity
Pearson's r=−0.010, p=0.952
Spearman's rho=−0.009, p=0.957
Triglycerides (mg/dL)
MT spot average intensity

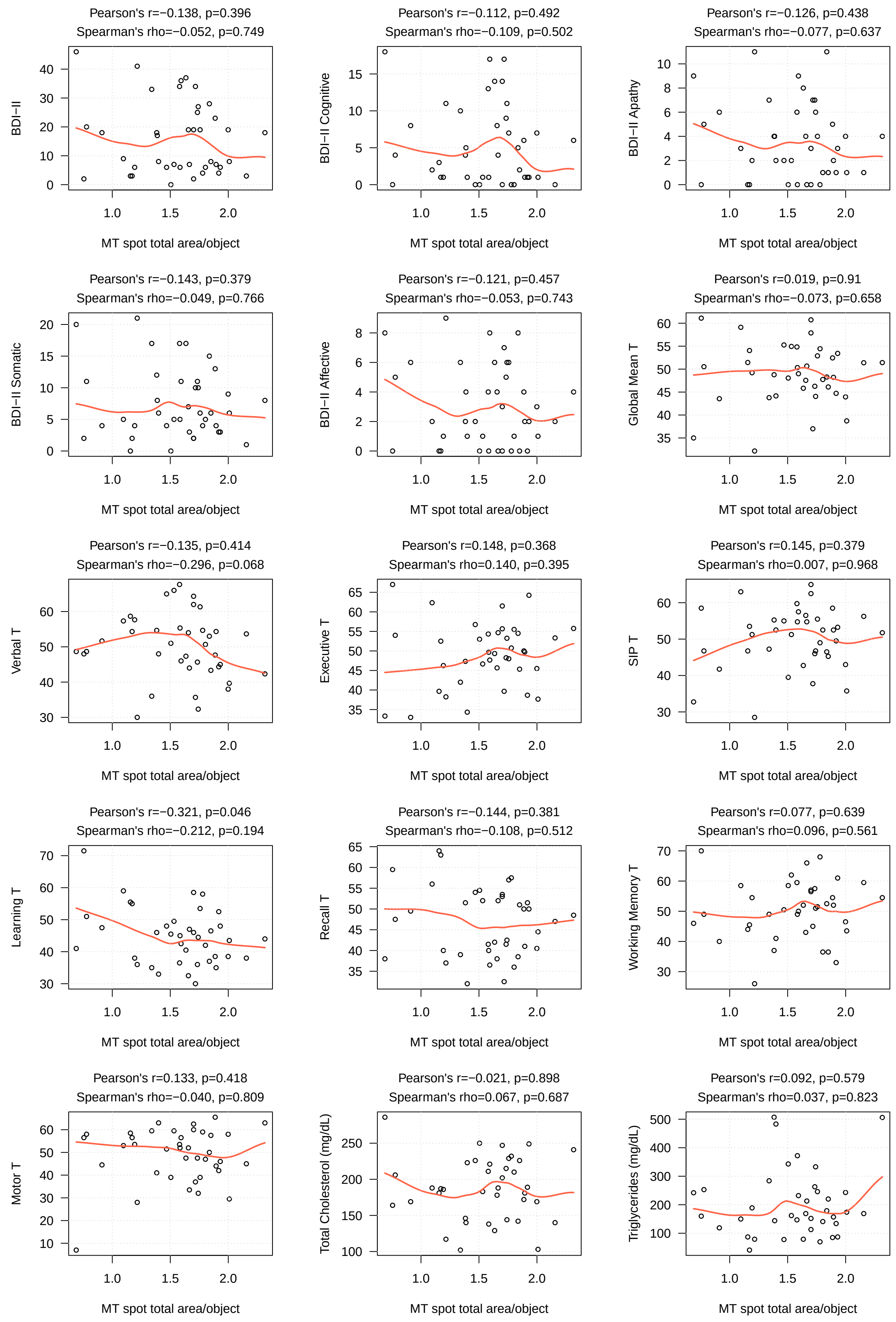
Pearson's r=−0.138, p=0.396
Spearman's rho=−0.052, p=0.749
BDI−II
MT spot total area/object
Pearson's r=−0.112, p=0.492
Spearman's rho=−0.109, p=0.502
BDI−II Cognitive
MT spot total area/object
Pearson's r=−0.126, p=0.438
Spearman's rho=−0.077, p=0.637
BDI−II Apathy
MT spot total area/object
Pearson's r=−0.143, p=0.379
Spearman's rho=−0.049, p=0.766
BDI−II Somatic
MT spot total area/object
Pearson's r=−0.121, p=0.457
Spearman's rho=−0.053, p=0.743
BDI−II Affective
MT spot total area/object
Pearson's r=0.019, p=0.91
Spearman's rho=−0.073, p=0.658
Global Mean T
MT spot total area/object
Pearson's r=−0.135, p=0.414
Spearman's rho=−0.296, p=0.068
Verbal T
MT spot total area/object
Pearson's r=0.148, p=0.368
Spearman's rho=0.140, p=0.395
Executive T
MT spot total area/object
Pearson's r=0.145, p=0.379
Spearman's rho=0.007, p=0.968
SIP T
MT spot total area/object
Pearson's r=−0.321, p=0.046
Spearman's rho=−0.212, p=0.194
Learning T
MT spot total area/object
Pearson's r=−0.144, p=0.381
Spearman's rho=−0.108, p=0.512
Recall T
MT spot total area/object
Pearson's r=0.077, p=0.639
Spearman's rho=0.096, p=0.561
Working Memory T
MT spot total area/object
Pearson's r=0.133, p=0.418
Spearman's rho=−0.040, p=0.809
Motor T
MT spot total area/object
Pearson's r=−0.021, p=0.898
Spearman's rho=0.067, p=0.687
Total Cholesterol (mg/dL)
MT spot total area/object
Pearson's r=0.092, p=0.579
Spearman's rho=0.037, p=0.823
Triglycerides (mg/dL)
MT spot total area/object

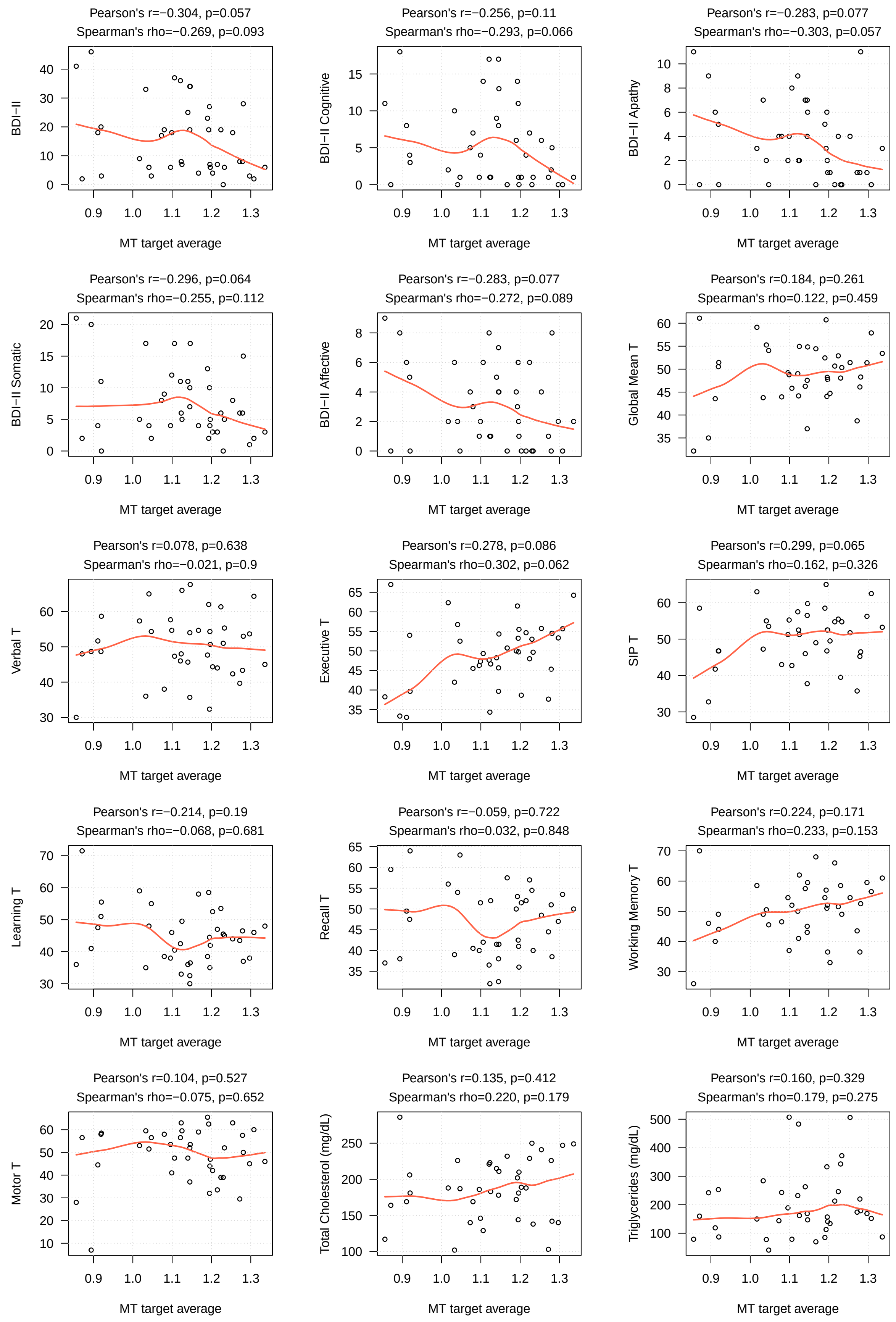
Pearson's r=−0.304, p=0.057
Spearman's rho=−0.269, p=0.093
BDI−II
MT target average
Pearson's r=−0.256, p=0.11
Spearman's rho=−0.293, p=0.066
BDI−II Cognitive
MT target average
Pearson's r=−0.283, p=0.077
Spearman's rho=−0.303, p=0.057
BDI−II Apathy
MT target average
Pearson's r=−0.296, p=0.064
Spearman's rho=−0.255, p=0.112
BDI−II Somatic
MT target average
Pearson's r=−0.283, p=0.077
Spearman's rho=−0.272, p=0.089
BDI−II Affective
MT target average
Pearson's r=0.184, p=0.261
Spearman's rho=0.122, p=0.459
Global Mean T
MT target average
Pearson's r=0.078, p=0.638
Spearman's rho=−0.021, p=0.9
Verbal T
MT target average
Pearson's r=0.278, p=0.086
Spearman's rho=0.302, p=0.062
Executive T
MT target average
Pearson's r=0.299, p=0.065
Spearman's rho=0.162, p=0.326
SIP T
MT target average
Pearson's r=−0.214, p=0.19
Spearman's rho=−0.068, p=0.681
Learning T
MT target average
Pearson's r=−0.059, p=0.722
Spearman's rho=0.032, p=0.848
Recall T
MT target average
Pearson's r=0.224, p=0.171
Spearman's rho=0.233, p=0.153
Working Memory T
MT target average
Pearson's r=0.104, p=0.527
Spearman's rho=−0.075, p=0.652
Motor T
MT target average
Pearson's r=0.135, p=0.412
Spearman's rho=0.220, p=0.179
Total Cholesterol (mg/dL)
MT target average
Pearson's r=0.160, p=0.329
Spearman's rho=0.179, p=0.275
Triglycerides (mg/dL)
MT target average

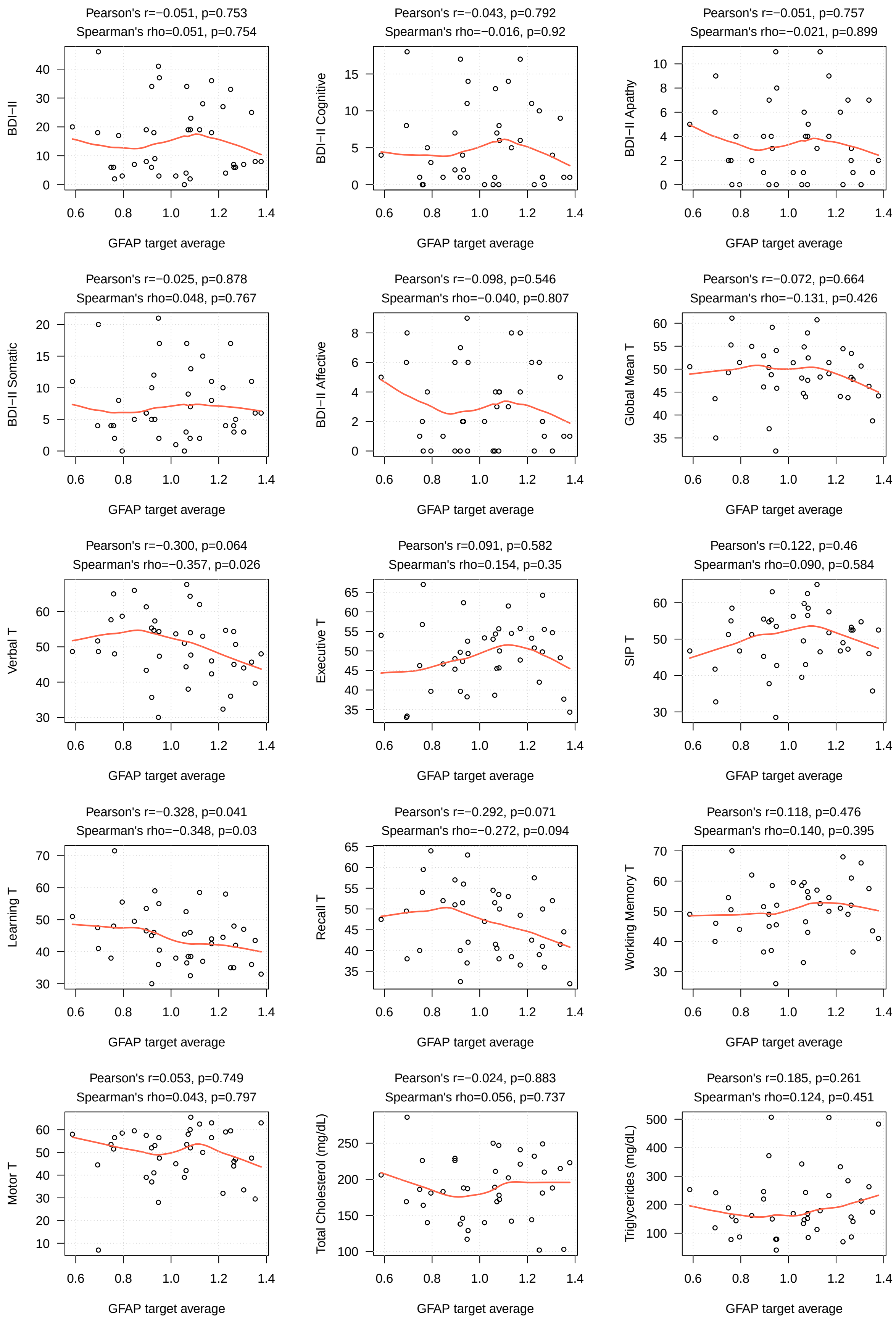
Pearson's r=−0.051, p=0.753
Spearman's rho=0.051, p=0.754
BDI−II
GFAP target average
Pearson's r=−0.043, p=0.792
Spearman's rho=−0.016, p=0.92
BDI−II Cognitive
GFAP target average
Pearson's r=−0.051, p=0.757
Spearman's rho=−0.021, p=0.899
BDI−II Apathy
GFAP target average
Pearson's r=−0.025, p=0.878
Spearman's rho=0.048, p=0.767
BDI−II Somatic
GFAP target average
Pearson's r=−0.098, p=0.546
Spearman's rho=−0.040, p=0.807
BDI−II Affective
GFAP target average
Pearson's r=−0.072, p=0.664
Spearman's rho=−0.131, p=0.426
Global Mean T
GFAP target average
Pearson's r=−0.300, p=0.064
Spearman's rho=−0.357, p=0.026
Verbal T
GFAP target average
Pearson's r=0.091, p=0.582
Spearman's rho=0.154, p=0.35
Executive T
GFAP target average
Pearson's r=0.122, p=0.46
Spearman's rho=0.090, p=0.584
SIP T
GFAP target average
Pearson's r=−0.328, p=0.041
Spearman's rho=−0.348, p=0.03
Learning T
GFAP target average
Pearson's r=−0.292, p=0.071
Spearman's rho=−0.272, p=0.094
Recall T
GFAP target average
Pearson's r=0.118, p=0.476
Spearman's rho=0.140, p=0.395
Working Memory T
GFAP target average
Pearson's r=0.053, p=0.749
Spearman's rho=0.043, p=0.797
Motor T
GFAP target average
Pearson's r=−0.024, p=0.883
Spearman's rho=0.056, p=0.737
Total Cholesterol (mg/dL)
GFAP target average
Pearson's r=0.185, p=0.261
Spearman's rho=0.124, p=0.451
Triglycerides (mg/dL)
GFAP target average

The following figure shows Pearson's correlations for associations between outcomes and predictors.

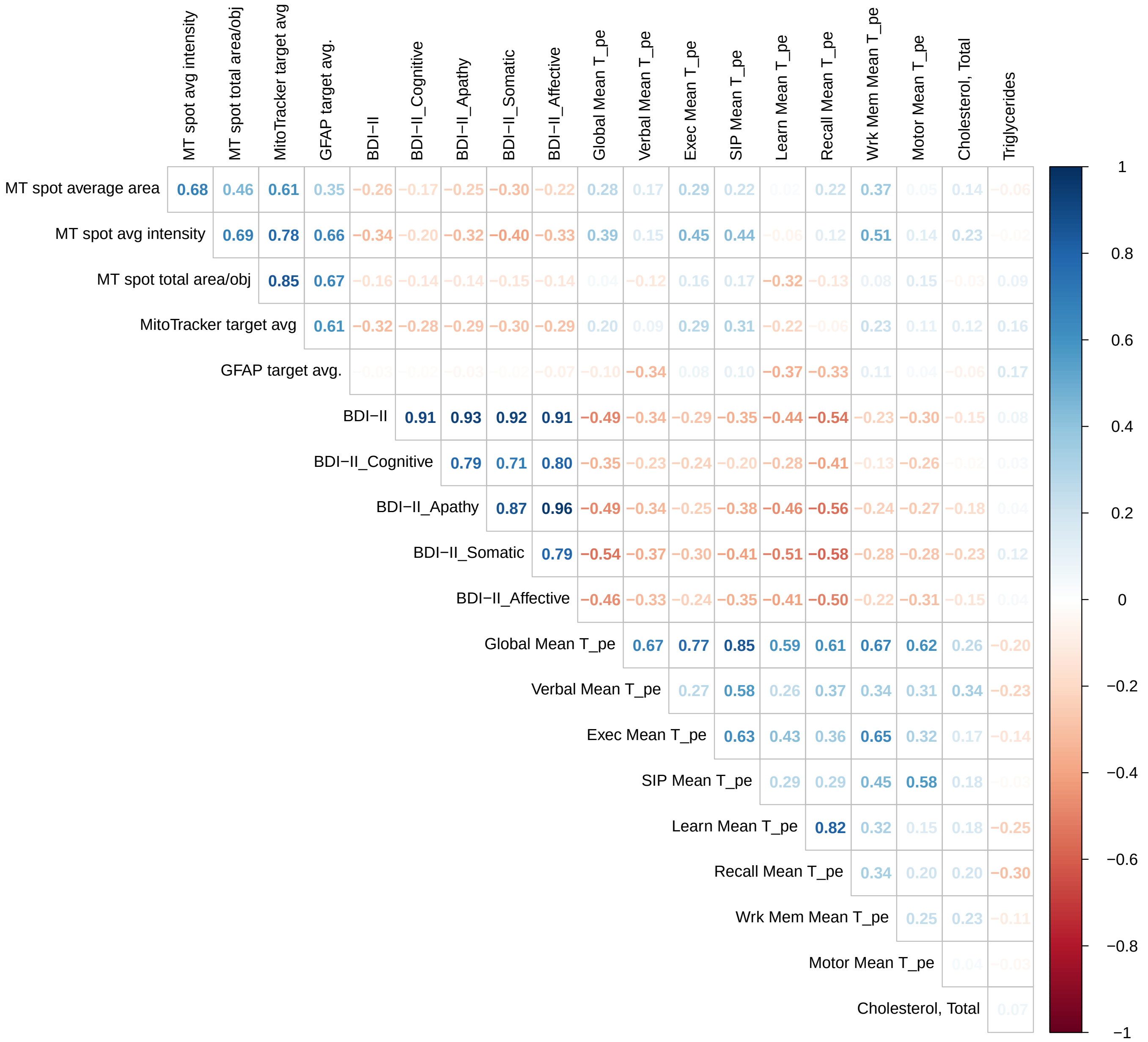

The above figure shows a general pattern of negative correlations between the MT predictors and BDI-II scores and mostly positive correlations between the predictors and cognitive T-scores, with exception of Learning domain. GFAP target average was negatively correlated with cognitive outcomes. A more detailed summary is given in section 2.3.

2.2 Simple associations with covariates

The tests shown below are not corrected for multiple testing. Due to small group sizes, ethnicity groups were combined into 2: white and non-white. Due to skewness and large number of observations tied at 0, the duration of D'drug exposure was dichotomized: exposed (> 0) and not exposed (0 months duration). Associations with both, numeric and binary, variables are shown, but only the binary variable is considered for inclusion into multivariable models.

Effect of the extreme outlier in ART duration was tested. Associations with numeric covariates were tested for non-linear patterns, where such were suspected. Results of these tests are summarized in section 2.3.

The plots below are clustered by outcomes.

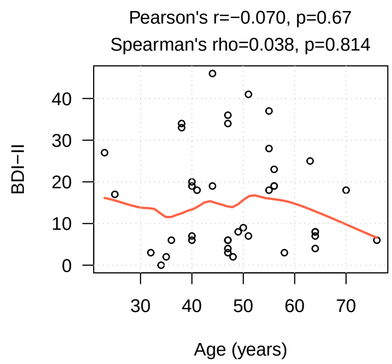

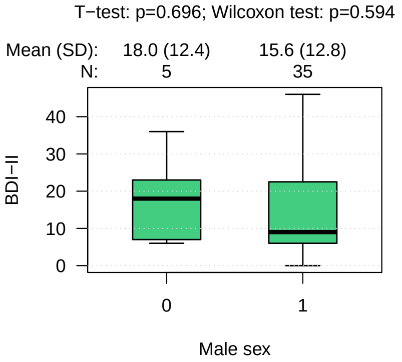

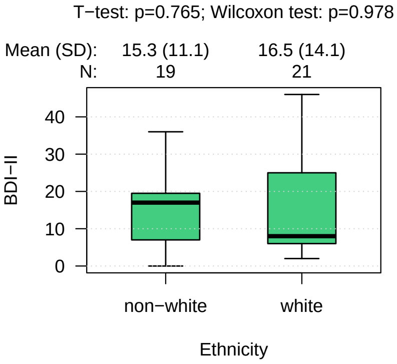

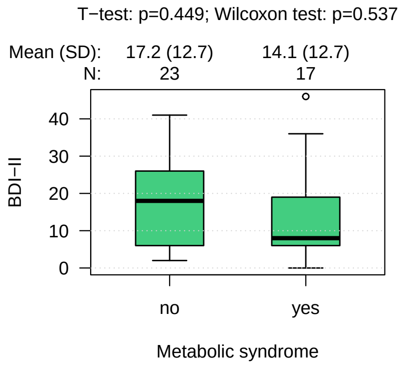

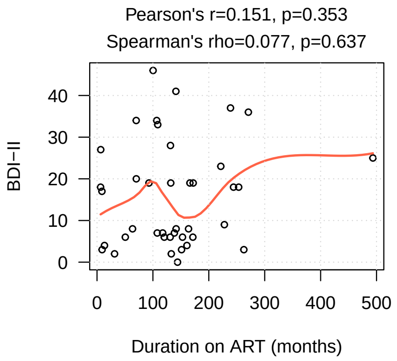

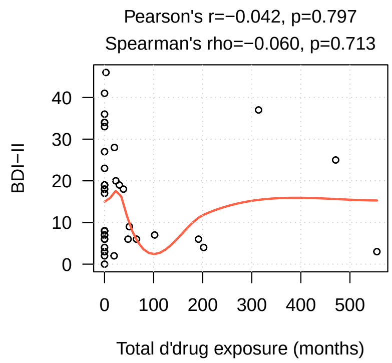

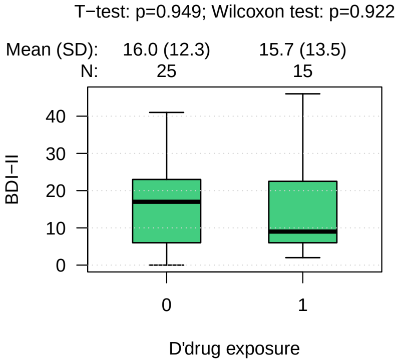

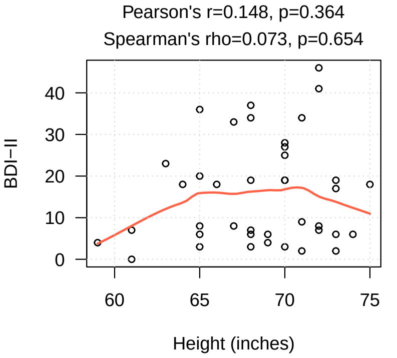

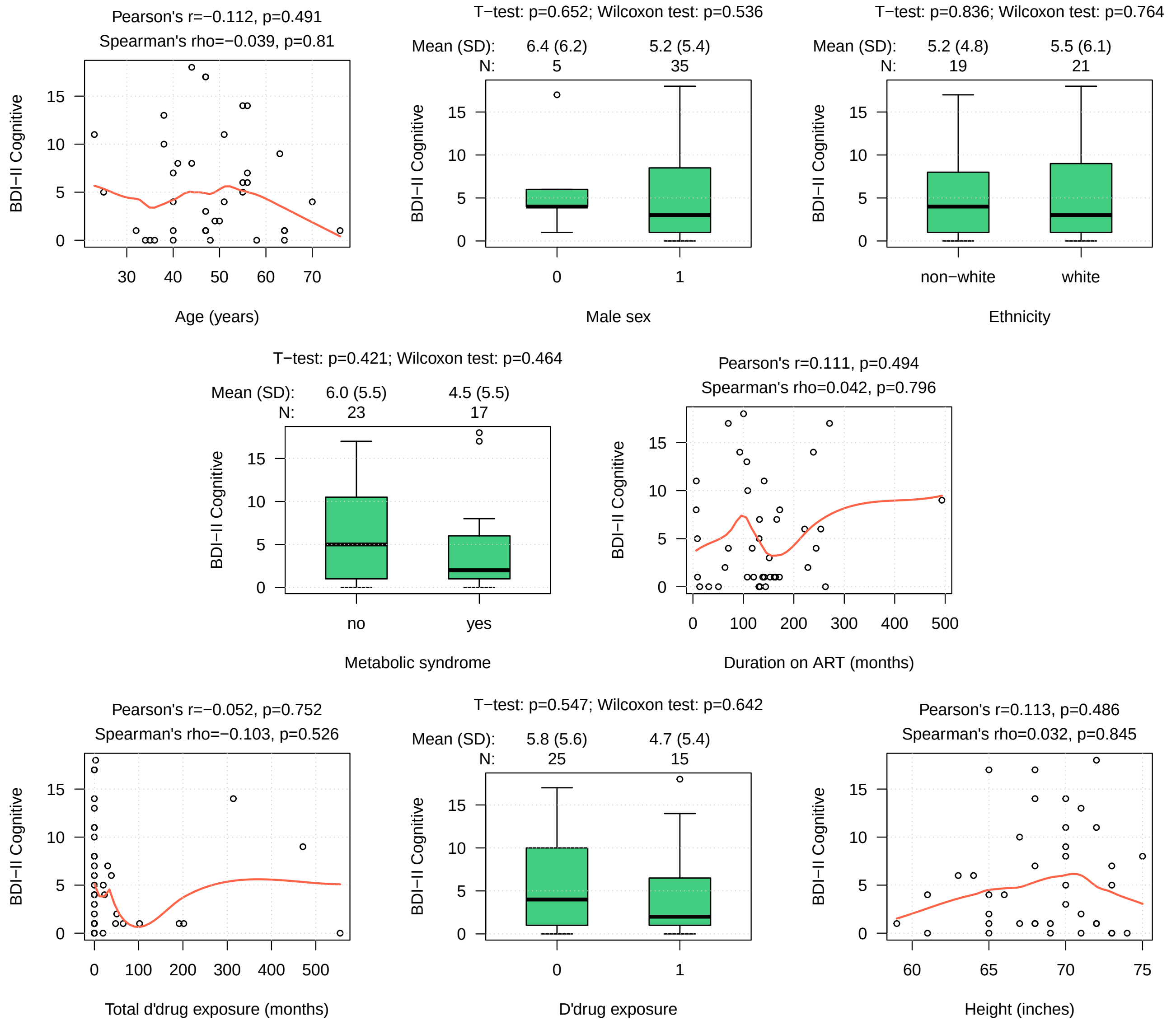

Pearson's r=−0.112, p=0.491
Spearman's rho=−0.039, p=0.81
BDI−II Cognitive
Age (years)
T−test: p=0.652; Wilcoxon test: p=0.536
Mean (SD): 6.4 (6.2) 5.2 (5.4)
N: 5 35
Male sex
T−test: p=0.836; Wilcoxon test: p=0.764
Mean (SD): 5.2 (4.8) 5.5 (6.1)
N: 19 21
non−white
white
Ethnicity
T−test: p=0.421; Wilcoxon test: p=0.464
Mean (SD): 6.0 (5.5) 4.5 (5.5)
N: 23 17
no
yes
Metabolic syndrome
Pearson's r=0.111, p=0.494
Spearman's rho=0.042, p=0.796
Duration on ART (months)
Pearson's r=−0.052, p=0.752
Spearman's rho=−0.103, p=0.526
Total d'drug exposure (months)
T−test: p=0.547; Wilcoxon test: p=0.642
Mean (SD): 5.8 (5.6) 4.7 (5.4)
N: 25 15
D'drug exposure
Pearson's r=0.113, p=0.486
Spearman's rho=0.032, p=0.845
Height (inches)

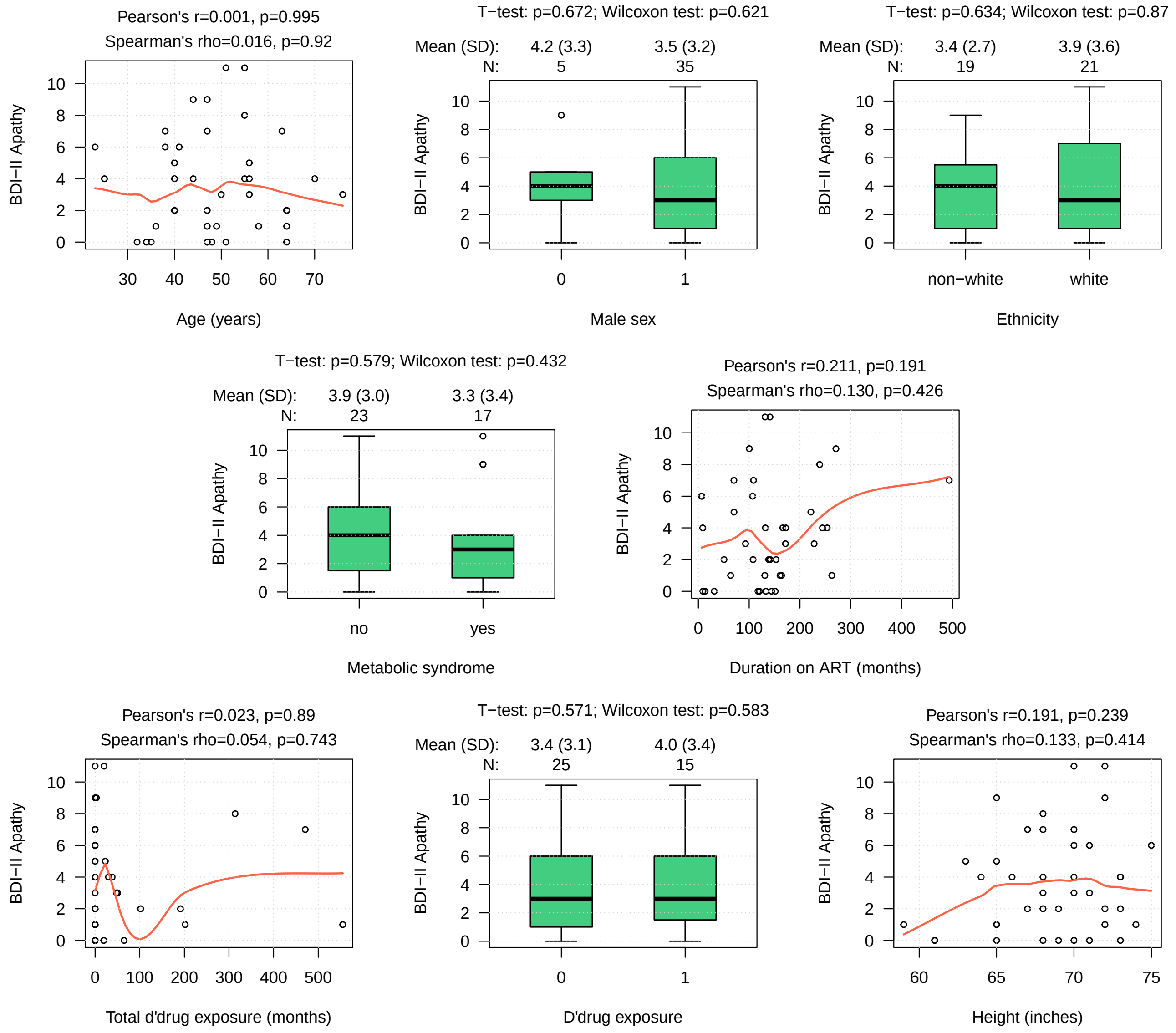
Pearson's r=0.001, p=0.995
Spearman's rho=0.016, p=0.92
BDI−II Apathy
Age (years)
T−test: p=0.672; Wilcoxon test: p=0.621
Mean (SD): 4.2 (3.3) 3.5 (3.2)
N: 5 35
BDI−II Apathy
Male sex
T−test: p=0.634; Wilcoxon test: p=0.87
Mean (SD): 3.4 (2.7) 3.9 (3.6)
N: 19 21
BDI−II Apathy
non−white
white
Ethnicity
T−test: p=0.579; Wilcoxon test: p=0.432
Mean (SD): 3.9 (3.0) 3.3 (3.4)
N: 23 17
BDI−II Apathy
no
yes
Metabolic syndrome
Pearson's r=0.211, p=0.191
Spearman's rho=0.130, p=0.426
BDI−II Apathy
Duration on ART (months)
Pearson's r=0.023, p=0.89
Spearman's rho=0.054, p=0.743
BDI−II Apathy
Total d'drug exposure (months)
T−test: p=0.571; Wilcoxon test: p=0.583
Mean (SD): 3.4 (3.1) 4.0 (3.4)
N: 25 15
BDI−II Apathy
D'drug exposure
Pearson's r=0.191, p=0.239
Spearman's rho=0.133, p=0.414
BDI−II Apathy
Height (inches)

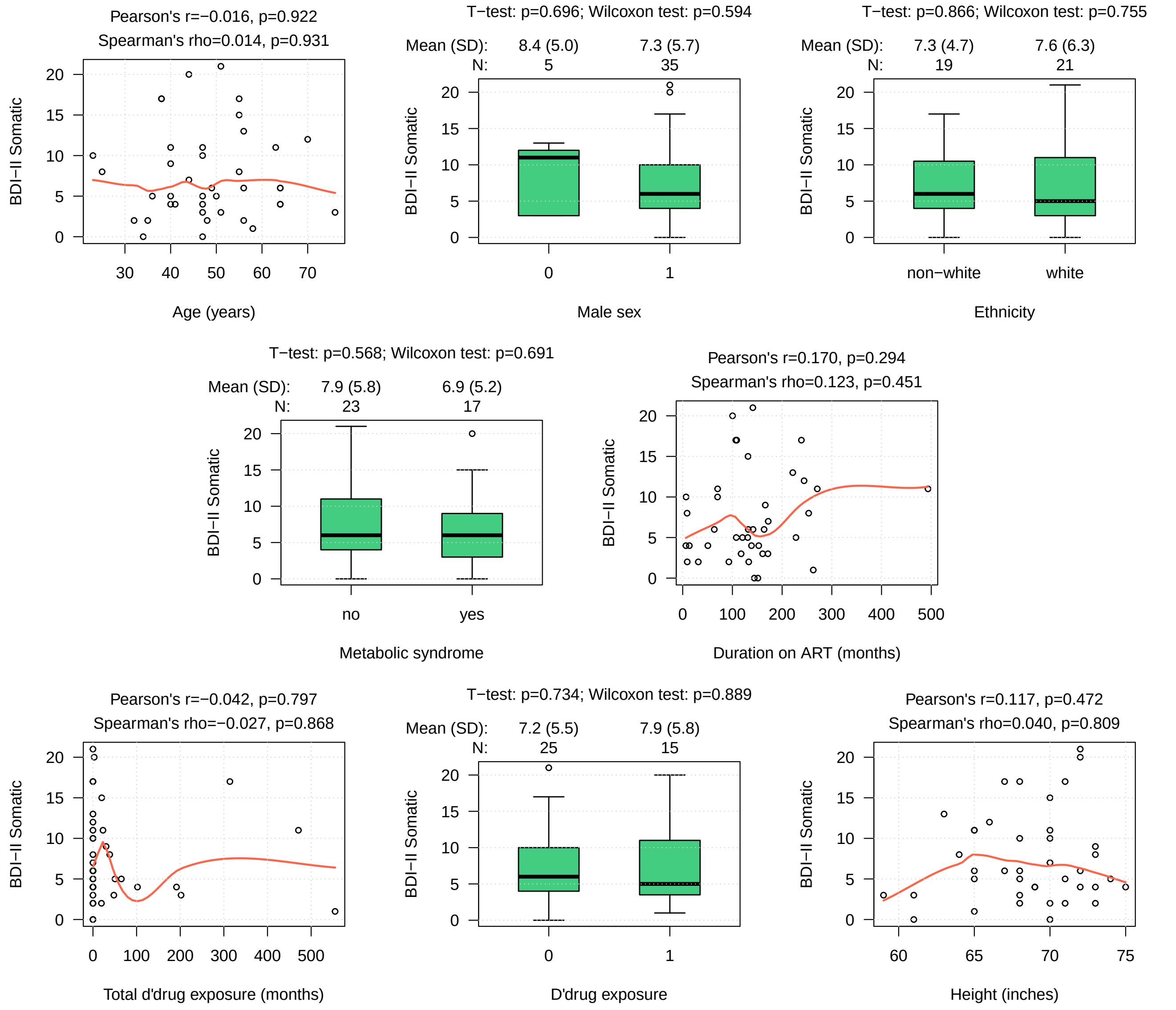
Pearson's r=−0.016, p=0.922
Spearman's rho=0.014, p=0.931
BDI−II Somatic
Age (years)
T−test: p=0.696; Wilcoxon test: p=0.594
Mean (SD): 8.4 (5.0) 7.3 (5.7)
N: 5 35
BDI−II Somatic
Male sex
T−test: p=0.866; Wilcoxon test: p=0.755
Mean (SD): 7.3 (4.7) 7.6 (6.3)
N: 19 21
BDI−II Somatic
non−white
white
Ethnicity
T−test: p=0.568; Wilcoxon test: p=0.691
Mean (SD): 7.9 (5.8) 6.9 (5.2)
N: 23 17
BDI−II Somatic
no
yes
Metabolic syndrome
Pearson's r=0.170, p=0.294
Spearman's rho=0.123, p=0.451
BDI−II Somatic
Duration on ART (months)
Pearson's r=−0.042, p=0.797
Spearman's rho=−0.027, p=0.868
BDI−II Somatic
Total d'drug exposure (months)
T−test: p=0.734; Wilcoxon test: p=0.889
Mean (SD): 7.2 (5.5) 7.9 (5.8)
N: 25 15
BDI−II Somatic
D'drug exposure
Pearson's r=0.117, p=0.472
Spearman's rho=0.040, p=0.809
BDI−II Somatic
Height (inches)

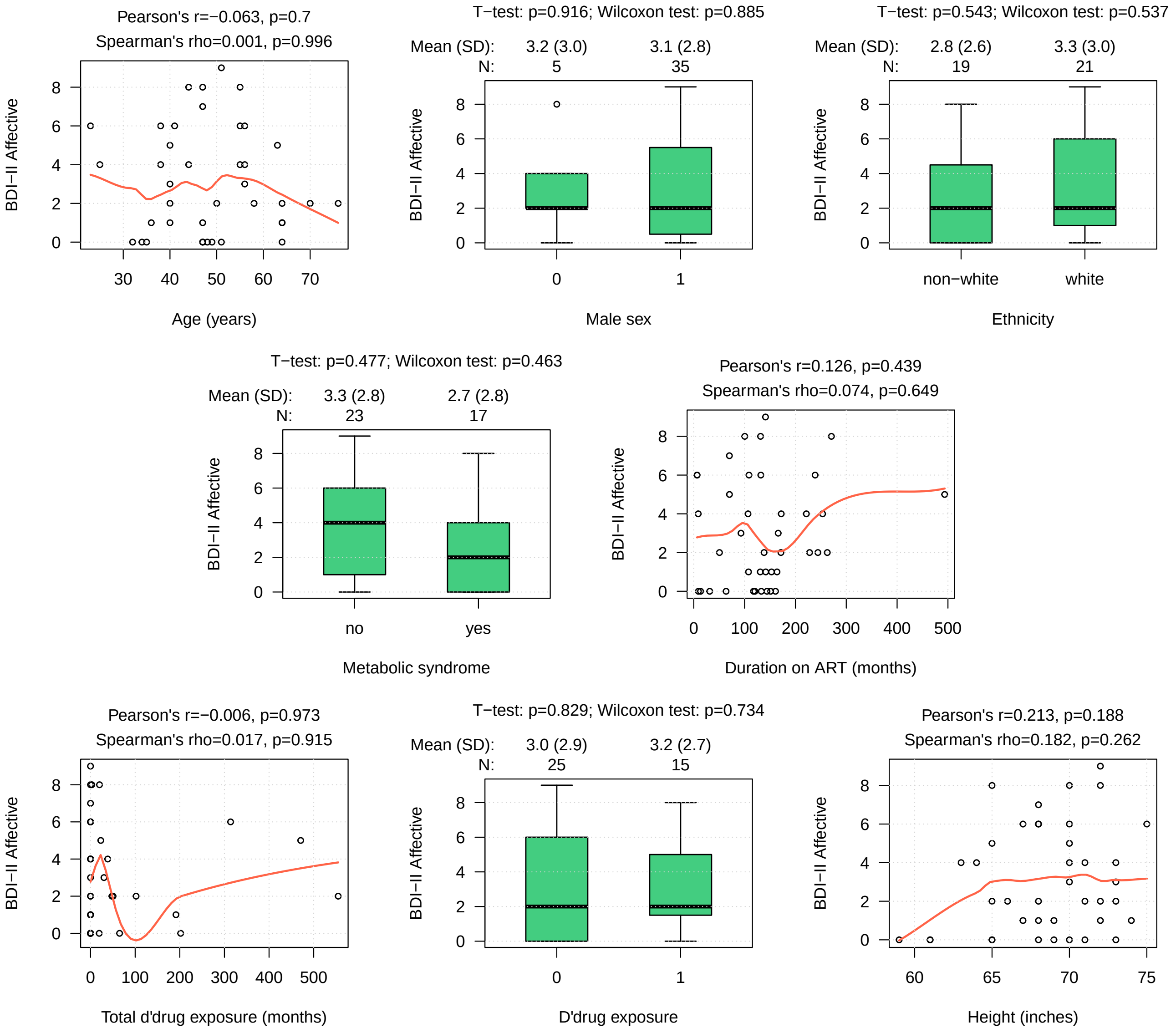
Pearson's r=−0.063, p=0.7
Spearman's rho=0.001, p=0.996
BDI−II Affective
Age (years)
T−test: p=0.916; Wilcoxon test: p=0.885
Mean (SD): 3.2 (3.0) 3.1 (2.8)
N: 5 35
BDI−II Affective
Male sex
T−test: p=0.543; Wilcoxon test: p=0.537
Mean (SD): 2.8 (2.6) 3.3 (3.0)
N: 19 21
BDI−II Affective
non−white
white
Ethnicity
T−test: p=0.477; Wilcoxon test: p=0.463
Mean (SD): 3.3 (2.8) 2.7 (2.8)
N: 23 17
BDI−II Affective
no
yes
Metabolic syndrome
Pearson's r=0.126, p=0.439
Spearman's rho=0.074, p=0.649
BDI−II Affective
Duration on ART (months)
Pearson's r=−0.006, p=0.973
Spearman's rho=0.017, p=0.915
BDI−II Affective
Total d'drug exposure (months)
T−test: p=0.829; Wilcoxon test: p=0.734
Mean (SD): 3.0 (2.9) 3.2 (2.7)
N: 25 15
BDI−II Affective
D'drug exposure
Pearson's r=0.213, p=0.188
Spearman's rho=0.182, p=0.262
BDI−II Affective
Height (inches)

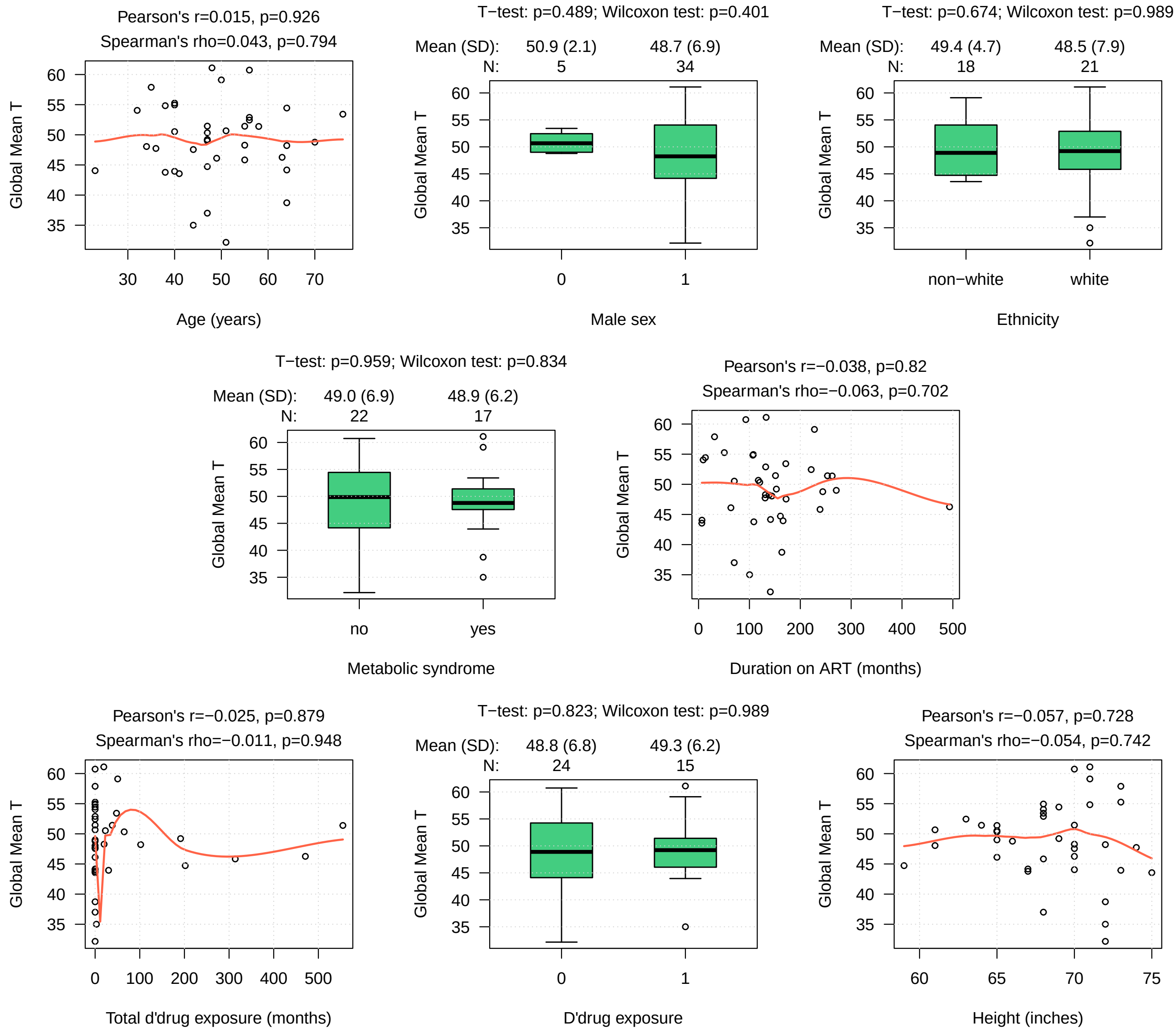
Pearson's r=0.015, p=0.926
Spearman's rho=0.043, p=0.794
Global Mean T
Age (years)
T−test: p=0.489; Wilcoxon test: p=0.401
Mean (SD): 50.9 (2.1) 48.7 (6.9)
N: 5 34
Male sex
T−test: p=0.674; Wilcoxon test: p=0.989
Mean (SD): 49.4 (4.7) 48.5 (7.9)
N: 18 21
non−white
white
Ethnicity
T−test: p=0.959; Wilcoxon test: p=0.834
Mean (SD): 49.0 (6.9) 48.9 (6.2)
N: 22 17
no
yes
Metabolic syndrome
Pearson's r=−0.038, p=0.82
Spearman's rho=−0.063, p=0.702
Duration on ART (months)
Pearson's r=−0.025, p=0.879
Spearman's rho=−0.011, p=0.948
Total d'drug exposure (months)
T−test: p=0.823; Wilcoxon test: p=0.989
Mean (SD): 48.8 (6.8) 49.3 (6.2)
N: 24 15
D'drug exposure
Pearson's r=−0.057, p=0.728
Spearman's rho=−0.054, p=0.742
Height (inches)

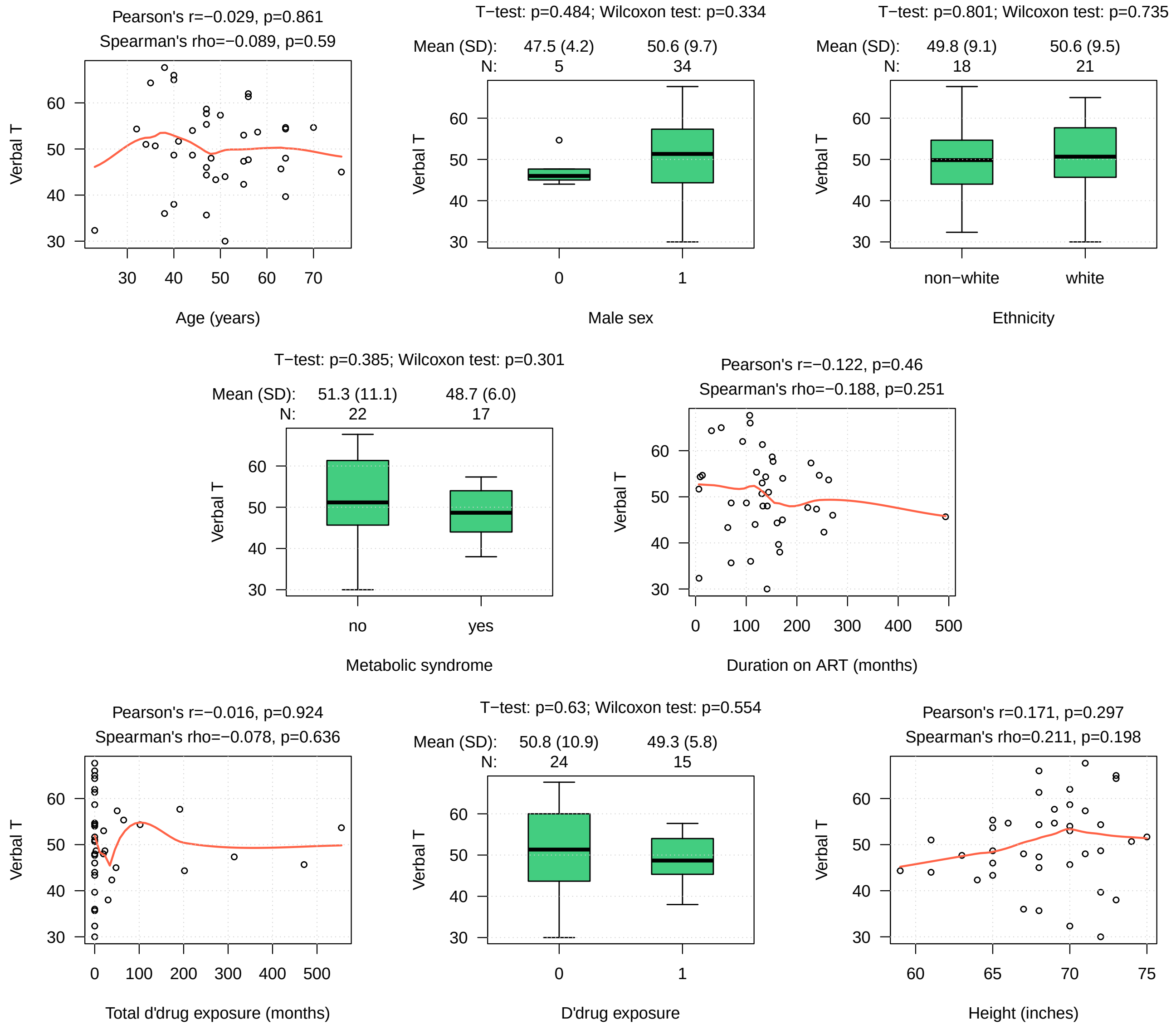
Pearson's r=−0.029, p=0.861
Spearman's rho=−0.089, p=0.59
Verbal T
Age (years)
T−test: p=0.484; Wilcoxon test: p=0.334
Mean (SD): 47.5 (4.2) 50.6 (9.7)
N: 5 34
Verbal T
0 1
Male sex
T−test: p=0.801; Wilcoxon test: p=0.735
Mean (SD): 49.8 (9.1) 50.6 (9.5)
N: 18 21
Verbal T
non−white white
Ethnicity
T−test: p=0.385; Wilcoxon test: p=0.301
Mean (SD): 51.3 (11.1) 48.7 (6.0)
N: 22 17
Verbal T
no yes
Metabolic syndrome
Pearson's r=−0.122, p=0.46
Spearman's rho=−0.188, p=0.251
Verbal T
Duration on ART (months)
Pearson's r=−0.016, p=0.924
Spearman's rho=−0.078, p=0.636
Verbal T
Total d'drug exposure (months)
T−test: p=0.63; Wilcoxon test: p=0.554
Mean (SD): 50.8 (10.9) 49.3 (5.8)
N: 24 15
Verbal T
0 1
D'drug exposure
Pearson's r=0.171, p=0.297
Spearman's rho=0.211, p=0.198
Verbal T
Height (inches)

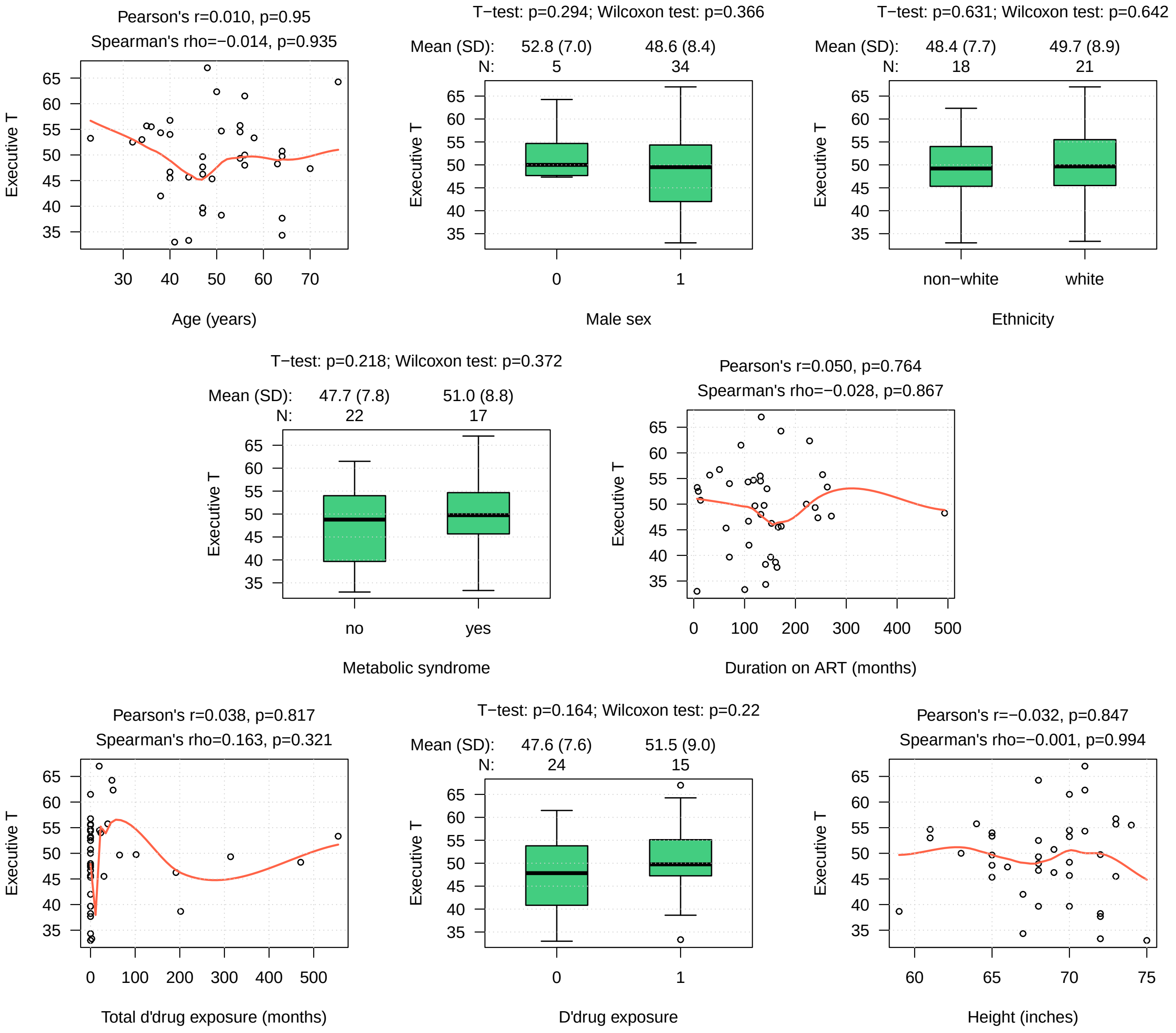
Pearson's r=0.010, p=0.95
Spearman's rho=−0.014, p=0.935
Executive T
Age (years)
T−test: p=0.294; Wilcoxon test: p=0.366
Mean (SD): 52.8 (7.0) 48.6 (8.4)
N: 5 34
Executive T
0 1
Male sex
T−test: p=0.631; Wilcoxon test: p=0.642
Mean (SD): 48.4 (7.7) 49.7 (8.9)
N: 18 21
Executive T
non−white white
Ethnicity
T−test: p=0.218; Wilcoxon test: p=0.372
Mean (SD): 47.7 (7.8) 51.0 (8.8)
N: 22 17
Executive T
no yes
Metabolic syndrome
Pearson's r=0.050, p=0.764
Spearman's rho=−0.028, p=0.867
Executive T
Duration on ART (months)
Pearson's r=0.038, p=0.817
Spearman's rho=0.163, p=0.321
Executive T
Total d'drug exposure (months)
T−test: p=0.164; Wilcoxon test: p=0.22
Mean (SD): 47.6 (7.6) 51.5 (9.0)
N: 24 15
Executive T
0 1
D'drug exposure
Pearson's r=−0.032, p=0.847
Spearman's rho=−0.001, p=0.994
Executive T
Height (inches)

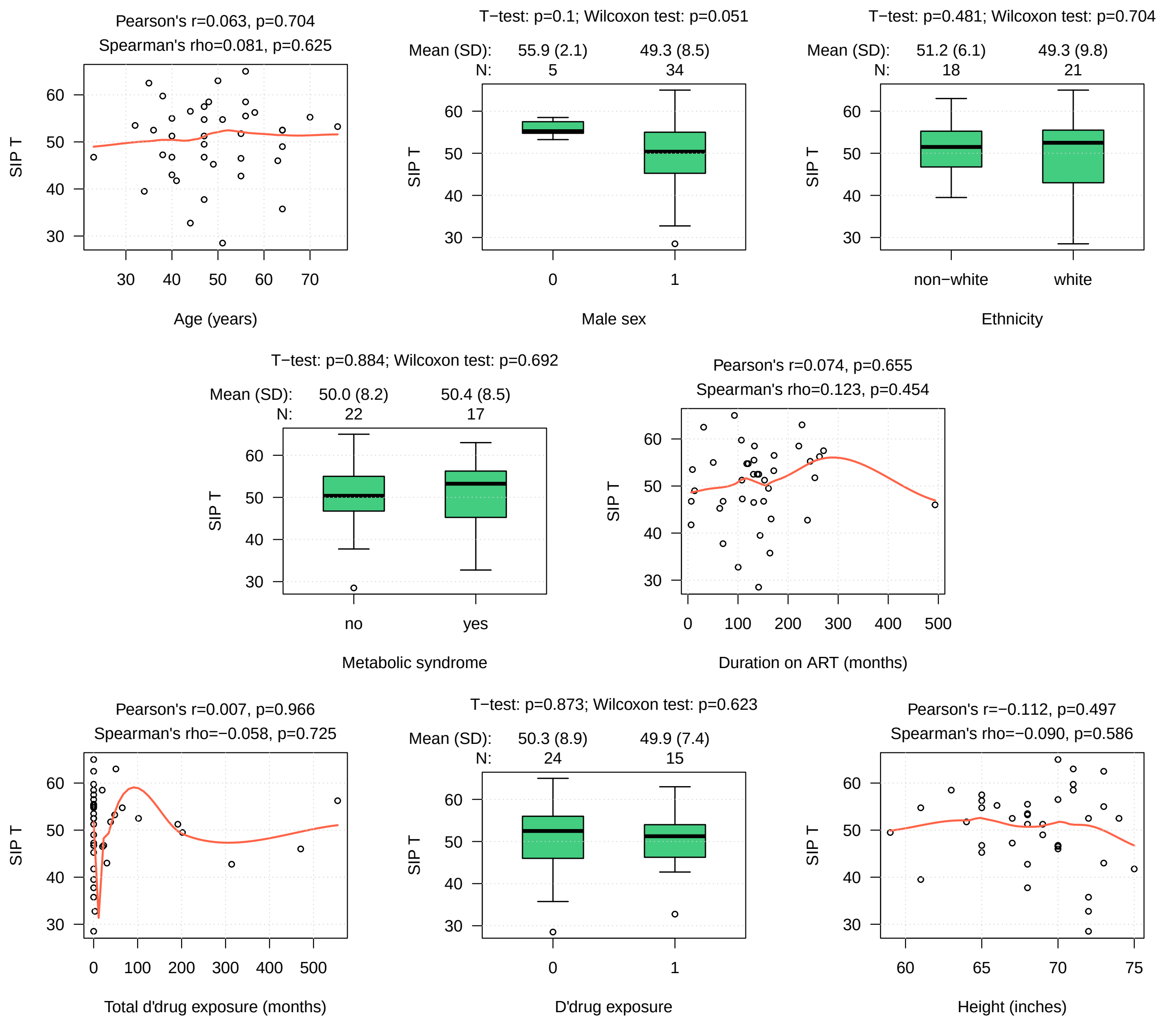
Pearson's r=0.063, p=0.704
Spearman's rho=0.081, p=0.625
SIP T
Age (years)
T−test: p=0.1; Wilcoxon test: p=0.051
Mean (SD): 55.9 (2.1) 49.3 (8.5)
N: 5 34
Male sex
T−test: p=0.481; Wilcoxon test: p=0.704
Mean (SD): 51.2 (6.1) 49.3 (9.8)
N: 18 21
non−white
white
Ethnicity
T−test: p=0.884; Wilcoxon test: p=0.692
Mean (SD): 50.0 (8.2) 50.4 (8.5)
N: 22 17
no
yes
Metabolic syndrome
Pearson's r=0.074, p=0.655
Spearman's rho=0.123, p=0.454
Duration on ART (months)
Pearson's r=0.007, p=0.966
Spearman's rho=−0.058, p=0.725
Total d'drug exposure (months)
T−test: p=0.873; Wilcoxon test: p=0.623
Mean (SD): 50.3 (8.9) 49.9 (7.4)
N: 24 15
D'drug exposure
Pearson's r=−0.112, p=0.497
Spearman's rho=−0.090, p=0.586
Height (inches)

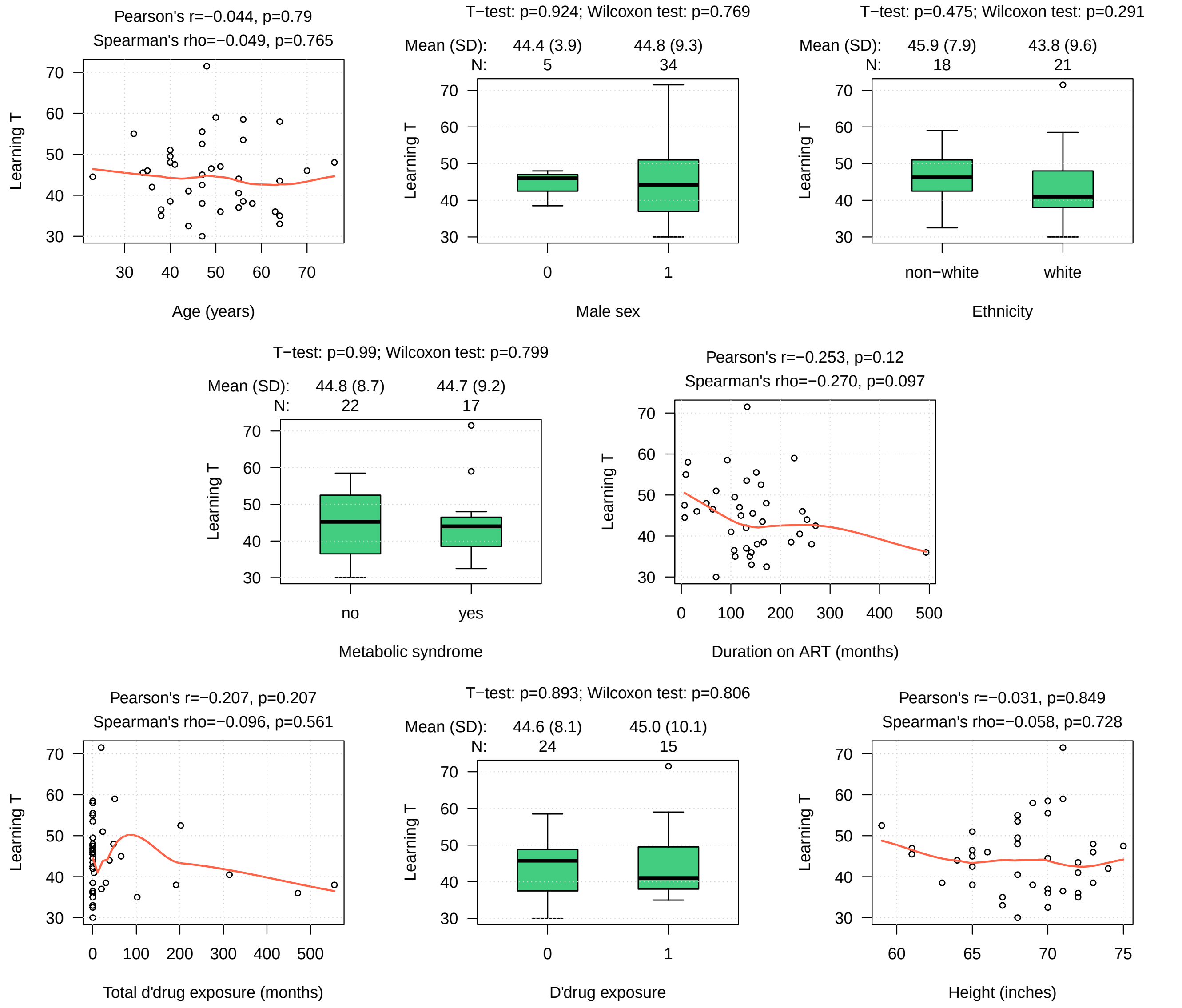
Pearson's r=−0.044, p=0.79
Spearman's rho=−0.049, p=0.765
Learning T
Age (years)
T−test: p=0.924; Wilcoxon test: p=0.769
Mean (SD): 44.4 (3.9) 44.8 (9.3)
N: 5 34
Learning T
0
1
Male sex
T−test: p=0.475; Wilcoxon test: p=0.291
Mean (SD): 45.9 (7.9) 43.8 (9.6)
N: 18 21
Learning T
non−white
white
Ethnicity
T−test: p=0.99; Wilcoxon test: p=0.799
Mean (SD): 44.8 (8.7) 44.7 (9.2)
N: 22 17
Learning T
no
yes
Metabolic syndrome
Pearson's r=−0.253, p=0.12
Spearman's rho=−0.270, p=0.097
Learning T
Duration on ART (months)
Pearson's r=−0.207, p=0.207
Spearman's rho=−0.096, p=0.561
Learning T
Total d'drug exposure (months)
T−test: p=0.893; Wilcoxon test: p=0.806
Mean (SD): 44.6 (8.1) 45.0 (10.1)
N: 24 15
Learning T
0
1
D'drug exposure
Pearson's r=−0.031, p=0.849
Spearman's rho=−0.058, p=0.728
Learning T
Height (inches)

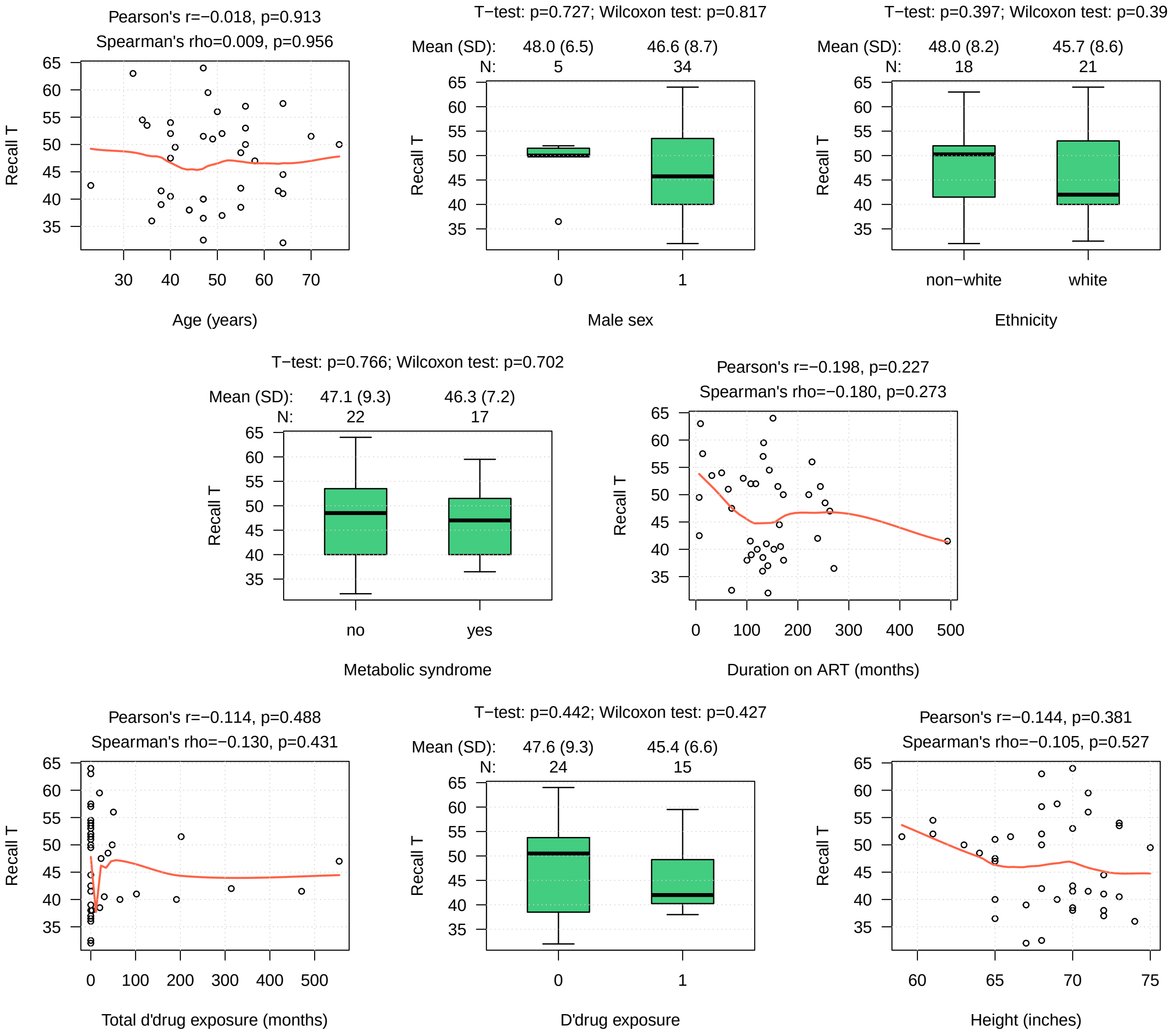
Pearson's r=−0.018, p=0.913
Spearman's rho=0.009, p=0.956
Recall T
Age (years)
T−test: p=0.727; Wilcoxon test: p=0.817
Mean (SD): 48.0 (6.5) 46.6 (8.7)
N: 5 34
Recall T
Male sex
T−test: p=0.397; Wilcoxon test: p=0.39
Mean (SD): 48.0 (8.2) 45.7 (8.6)
N: 18 21
Recall T
non−white
white
Ethnicity
T−test: p=0.766; Wilcoxon test: p=0.702
Mean (SD): 47.1 (9.3) 46.3 (7.2)
N: 22 17
Recall T
no
yes
Metabolic syndrome
Pearson's r=−0.198, p=0.227
Spearman's rho=−0.180, p=0.273
Recall T
Duration on ART (months)
Pearson's r=−0.114, p=0.488
Spearman's rho=−0.130, p=0.431
Recall T
Total d'drug exposure (months)
T−test: p=0.442; Wilcoxon test: p=0.427
Mean (SD): 47.6 (9.3) 45.4 (6.6)
N: 24 15
Recall T
D'drug exposure
Pearson's r=−0.144, p=0.381
Spearman's rho=−0.105, p=0.527
Recall T
Height (inches)

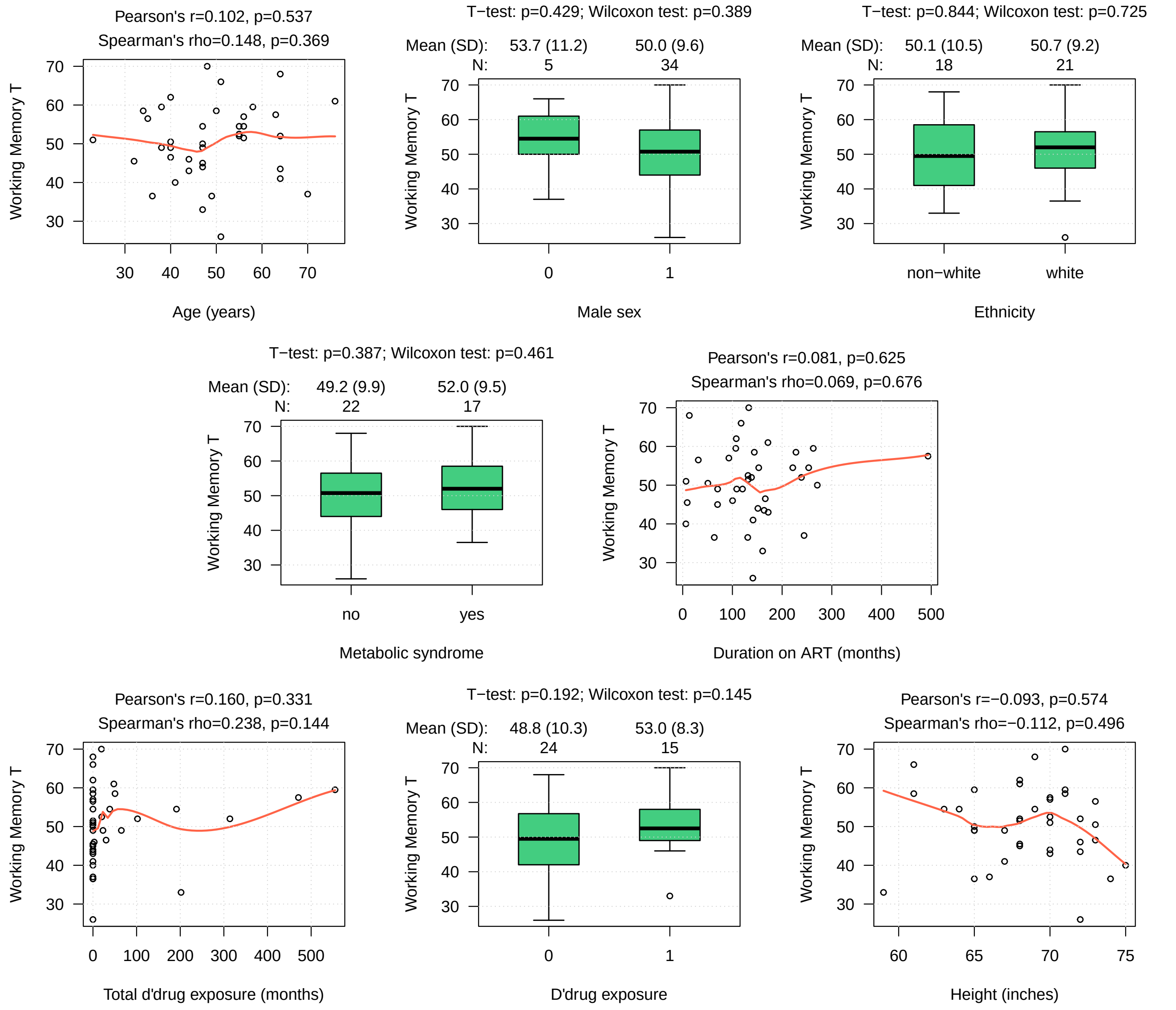
Pearson's r=0.102, p=0.537
Spearman's rho=0.148, p=0.369
Working Memory T
Age (years)
T−test: p=0.429; Wilcoxon test: p=0.389
Mean (SD): 53.7 (11.2) 50.0 (9.6)
N: 5 34
Working Memory T
Male sex
T−test: p=0.844; Wilcoxon test: p=0.725
Mean (SD): 50.1 (10.5) 50.7 (9.2)
N: 18 21
non−white
white
Working Memory T
Ethnicity
T−test: p=0.387; Wilcoxon test: p=0.461
Mean (SD): 49.2 (9.9) 52.0 (9.5)
N: 22 17
no
yes
Working Memory T
Metabolic syndrome
Pearson's r=0.081, p=0.625
Spearman's rho=0.069, p=0.676
Working Memory T
Duration on ART (months)
Pearson's r=0.160, p=0.331
Spearman's rho=0.238, p=0.144
Working Memory T
Total d'drug exposure (months)
T−test: p=0.192; Wilcoxon test: p=0.145
Mean (SD): 48.8 (10.3) 53.0 (8.3)
N: 24 15
Working Memory T
D'drug exposure
Pearson's r=−0.093, p=0.574
Spearman's rho=−0.112, p=0.496
Working Memory T
Height (inches)

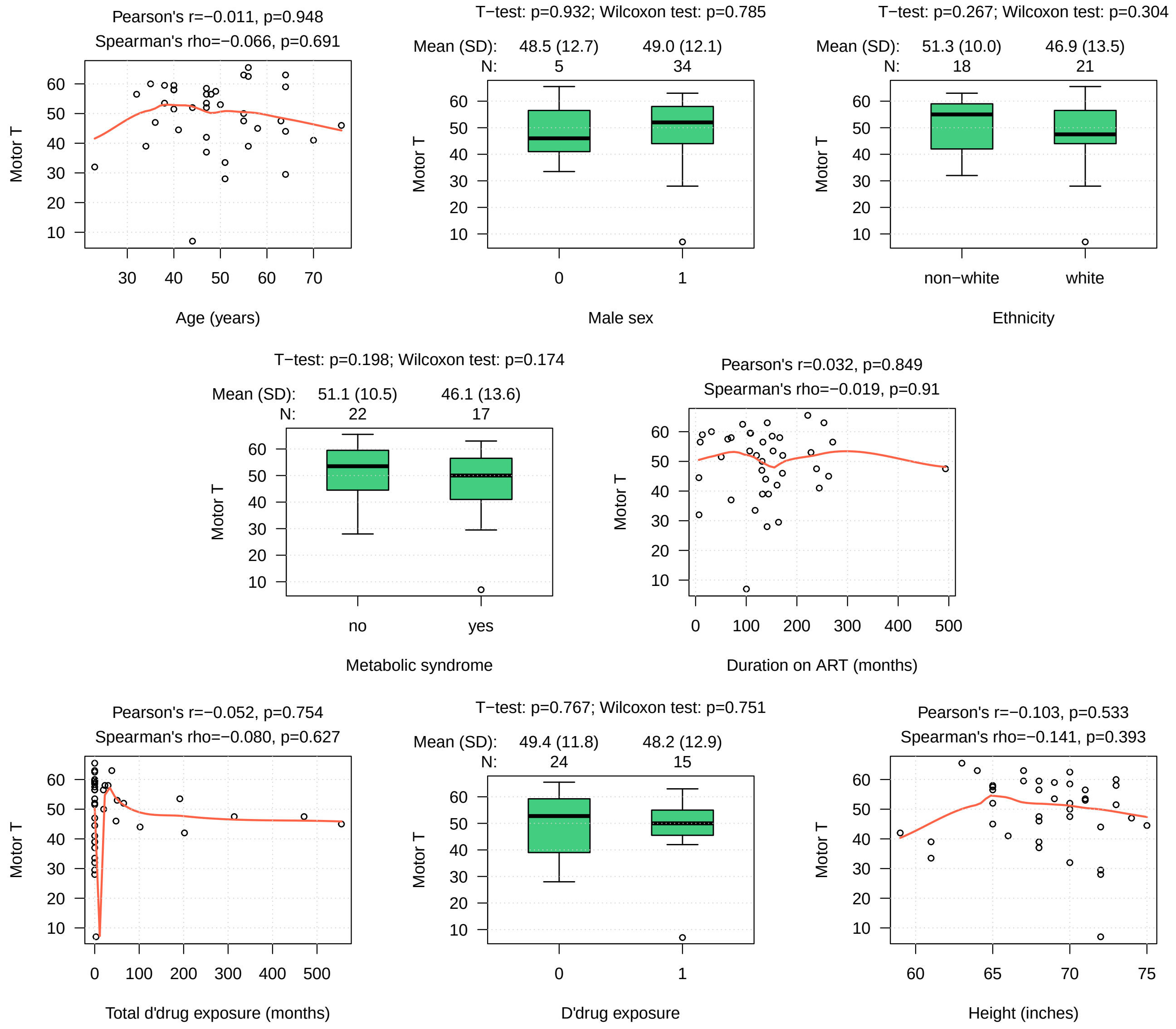
Pearson's r=−0.011, p=0.948
Spearman's rho=−0.066, p=0.691
Motor T
Age (years)
T−test: p=0.932; Wilcoxon test: p=0.785
Mean (SD): 48.5 (12.7) 49.0 (12.1)
N: 5 34
Motor T
Male sex
T−test: p=0.267; Wilcoxon test: p=0.304
Mean (SD): 51.3 (10.0) 46.9 (13.5)
N: 18 21
Motor T
non−white
white
Ethnicity
T−test: p=0.198; Wilcoxon test: p=0.174
Mean (SD): 51.1 (10.5) 46.1 (13.6)
N: 22 17
Motor T
no
yes
Metabolic syndrome
Pearson's r=0.032, p=0.849
Spearman's rho=−0.019, p=0.91
Motor T
Duration on ART (months)
Pearson's r=−0.052, p=0.754
Spearman's rho=−0.080, p=0.627
Motor T
Total d'drug exposure (months)
T−test: p=0.767; Wilcoxon test: p=0.751
Mean (SD): 49.4 (11.8) 48.2 (12.9)
N: 24 15
Motor T
D'drug exposure
Pearson's r=−0.103, p=0.533
Spearman's rho=−0.141, p=0.393
Motor T
Height (inches)

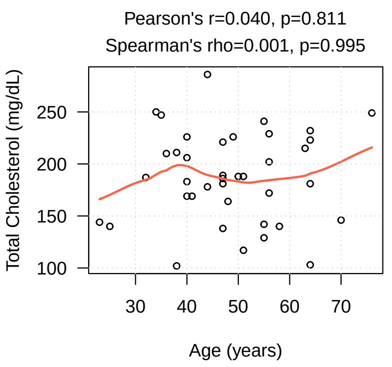
Pearson's r=0.040, p=0.811
Spearman's rho=0.001, p=0.995
Total Cholesterol (mg/dL)
Age (years)

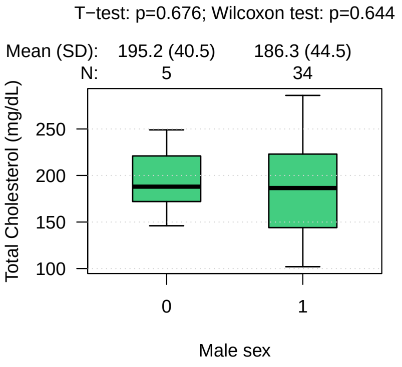
T−test: p=0.676; Wilcoxon test: p=0.644
Mean (SD): 195.2 (40.5) 186.3 (44.5)
N: 5 34
Total Cholesterol (mg/dL)
0 1
Male sex

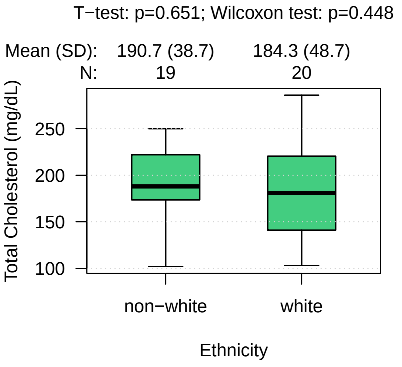
T−test: p=0.651; Wilcoxon test: p=0.448
Mean (SD): 190.7 (38.7) 184.3 (48.7)
N: 19 20
Total Cholesterol (mg/dL)
non−white white
Ethnicity

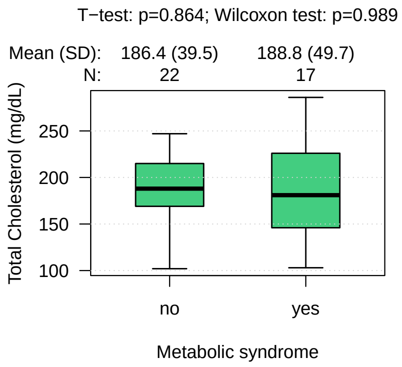
T−test: p=0.864; Wilcoxon test: p=0.989
Mean (SD): 186.4 (39.5) 188.8 (49.7)
N: 22 17
Total Cholesterol (mg/dL)
no yes
Metabolic syndrome

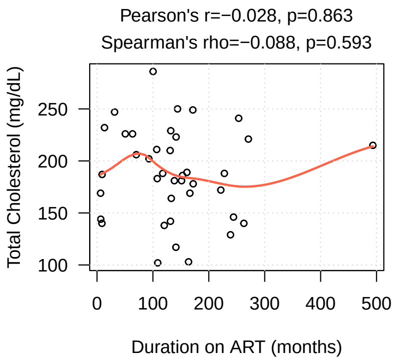
Pearson's r=−0.028, p=0.863
Spearman's rho=−0.088, p=0.593
Total Cholesterol (mg/dL)
Duration on ART (months)

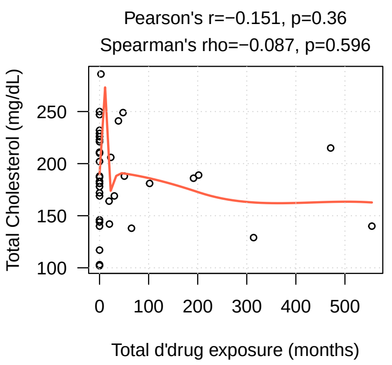
Pearson's r=−0.151, p=0.36
Spearman's rho=−0.087, p=0.596
Total Cholesterol (mg/dL)
Total d'drug exposure (months)

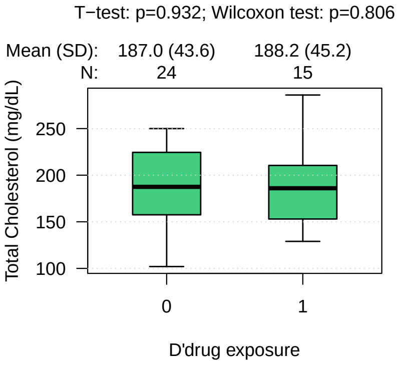
T−test: p=0.932; Wilcoxon test: p=0.806
Mean (SD): 187.0 (43.6) 188.2 (45.2)
N: 24 15
Total Cholesterol (mg/dL)
0 1
D'drug exposure

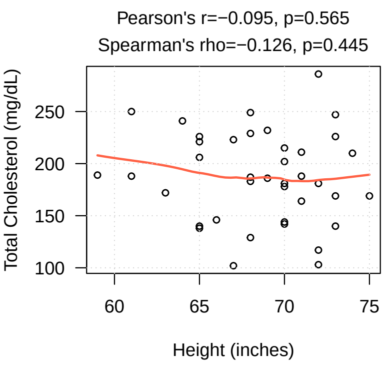
Pearson's r=−0.095, p=0.565
Spearman's rho=−0.126, p=0.445
Total Cholesterol (mg/dL)
Height (inches)

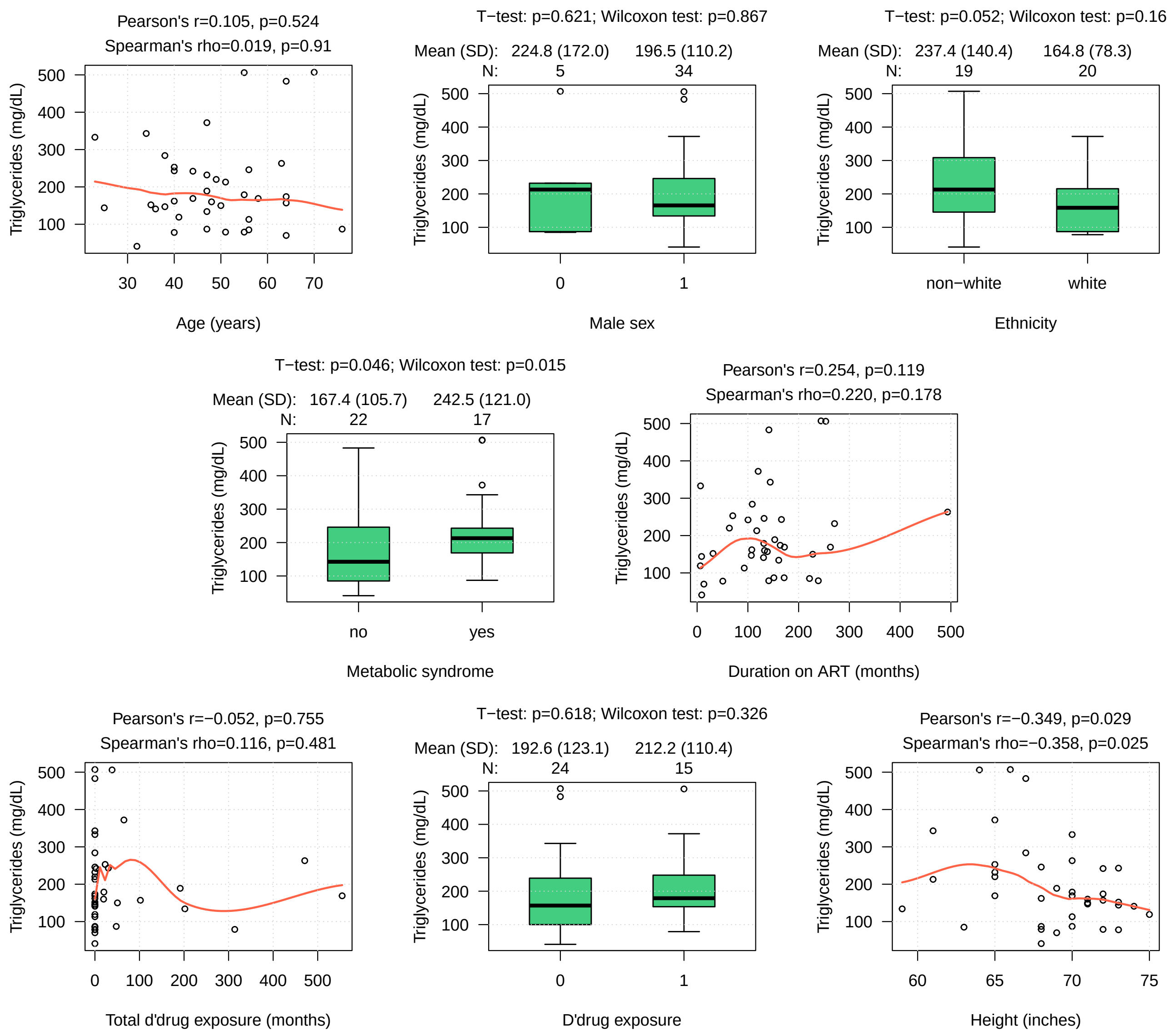
Pearson's r=0.105, p=0.524
Spearman's rho=0.019, p=0.91
Triglycerides (mg/dL)
Age (years)
T−test: p=0.621; Wilcoxon test: p=0.867
Mean (SD): 224.8 (172.0) 196.5 (110.2)
N: 5 34
Male sex
T−test: p=0.052; Wilcoxon test: p=0.16
Mean (SD): 237.4 (140.4) 164.8 (78.3)
N: 19 20
non−white
white
Ethnicity
T−test: p=0.046; Wilcoxon test: p=0.015
Mean (SD): 167.4 (105.7) 242.5 (121.0)
N: 22 17
no
yes
Metabolic syndrome
Pearson's r=0.254, p=0.119
Spearman's rho=0.220, p=0.178
Duration on ART (months)
Pearson's r=−0.052, p=0.755
Spearman's rho=0.116, p=0.481
Total d'drug exposure (months)
T−test: p=0.618; Wilcoxon test: p=0.326
Mean (SD): 192.6 (123.1) 212.2 (110.4)
N: 24 15
D'drug exposure
Pearson's r=−0.349, p=0.029
Spearman's rho=−0.358, p=0.025
Height (inches)

The plots below are clustered by predictors.

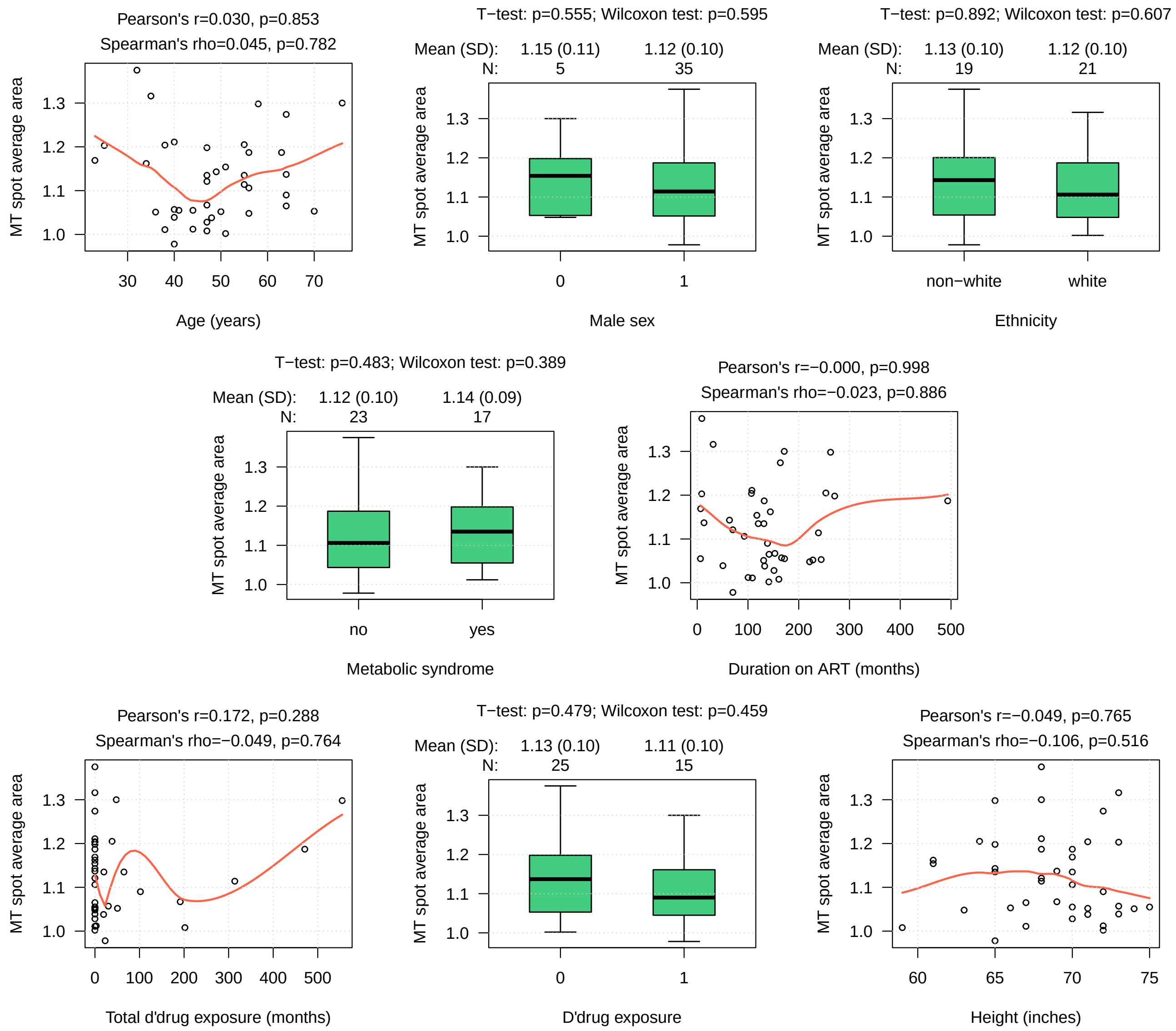

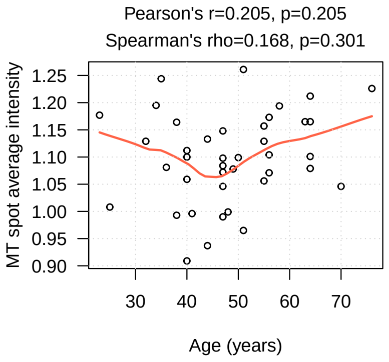
Pearson's r=0.205, p=0.205
Spearman's rho=0.168, p=0.301
MT spot average intensity
Age (years)

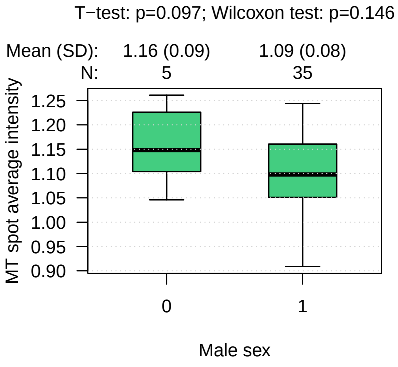
T−test: p=0.097; Wilcoxon test: p=0.146
Mean (SD): 1.16 (0.09) 1.09 (0.08)
N: 5 35
MT spot average intensity
0
1
Male sex

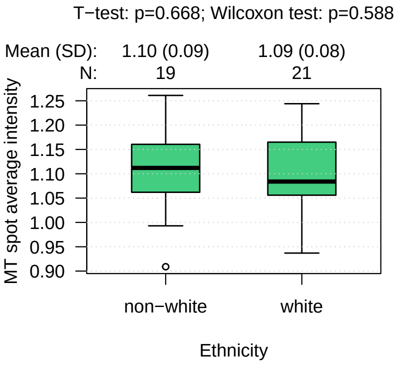
T−test: p=0.668; Wilcoxon test: p=0.588
Mean (SD): 1.10 (0.09) 1.09 (0.08)
N: 19 21
MT spot average intensity
non−white
white
Ethnicity

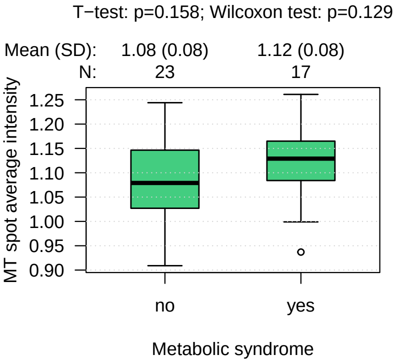
T−test: p=0.158; Wilcoxon test: p=0.129
Mean (SD): 1.08 (0.08) 1.12 (0.08)
N: 23 17
MT spot average intensity
no
yes
Metabolic syndrome

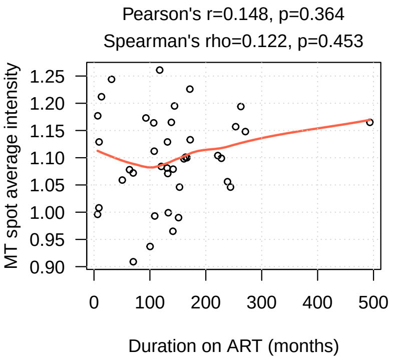
Pearson's r=0.148, p=0.364
Spearman's rho=0.122, p=0.453
MT spot average intensity
Duration on ART (months)

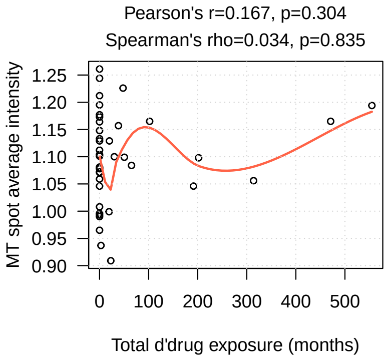
Pearson's r=0.167, p=0.304
Spearman's rho=0.034, p=0.835
MT spot average intensity
Total d'drug exposure (months)

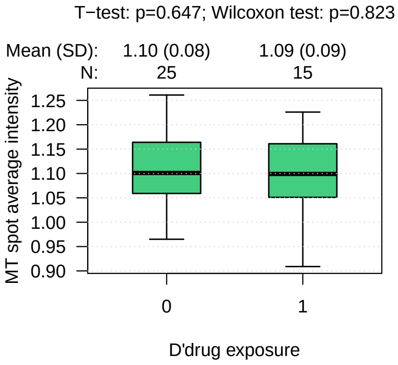
T−test: p=0.647; Wilcoxon test: p=0.823
Mean (SD): 1.10 (0.08) 1.09 (0.09)
N: 25 15
MT spot average intensity
0
1
D'drug exposure

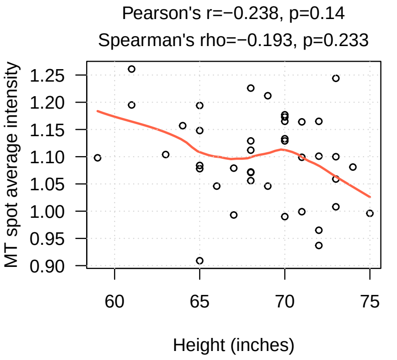
Pearson's r=−0.238, p=0.14
Spearman's rho=−0.193, p=0.233
MT spot average intensity
Height (inches)

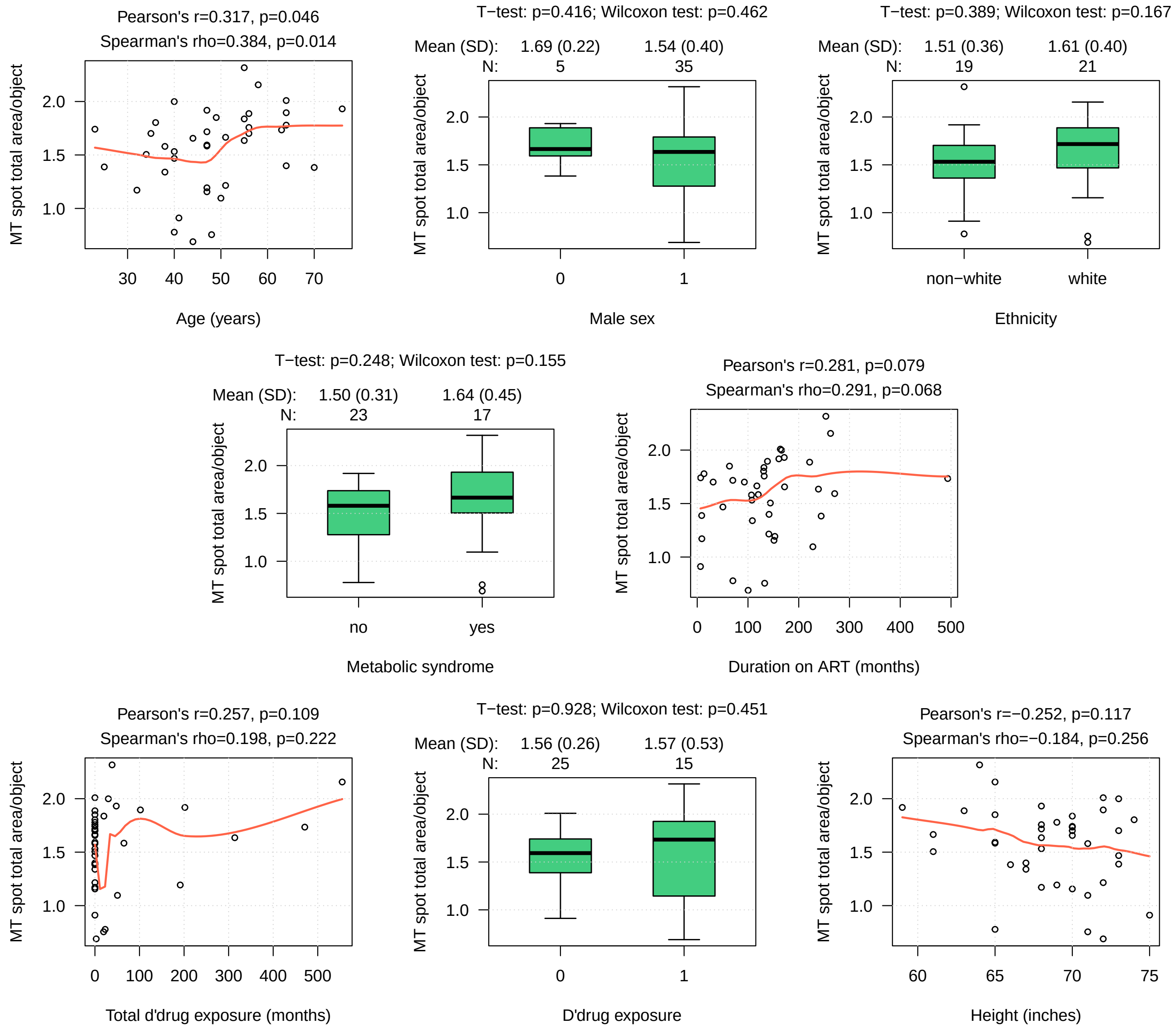
Pearson's r=0.317, p=0.046
Spearman's rho=0.384, p=0.014
MT spot total area/object
Age (years)
T−test: p=0.416; Wilcoxon test: p=0.462
Mean (SD): 1.69 (0.22) 1.54 (0.40)
N: 5 35
Male sex
T−test: p=0.389; Wilcoxon test: p=0.167
Mean (SD): 1.51 (0.36) 1.61 (0.40)
N: 19 21
non−white
white
Ethnicity
T−test: p=0.248; Wilcoxon test: p=0.155
Mean (SD): 1.50 (0.31) 1.64 (0.45)
N: 23 17
no
yes
Metabolic syndrome
Pearson's r=0.281, p=0.079
Spearman's rho=0.291, p=0.068
Duration on ART (months)
Pearson's r=0.257, p=0.109
Spearman's rho=0.198, p=0.222
Total d'drug exposure (months)
T−test: p=0.928; Wilcoxon test: p=0.451
Mean (SD): 1.56 (0.26) 1.57 (0.53)
N: 25 15
D'drug exposure
Pearson's r=−0.252, p=0.117
Spearman's rho=−0.184, p=0.256
Height (inches)

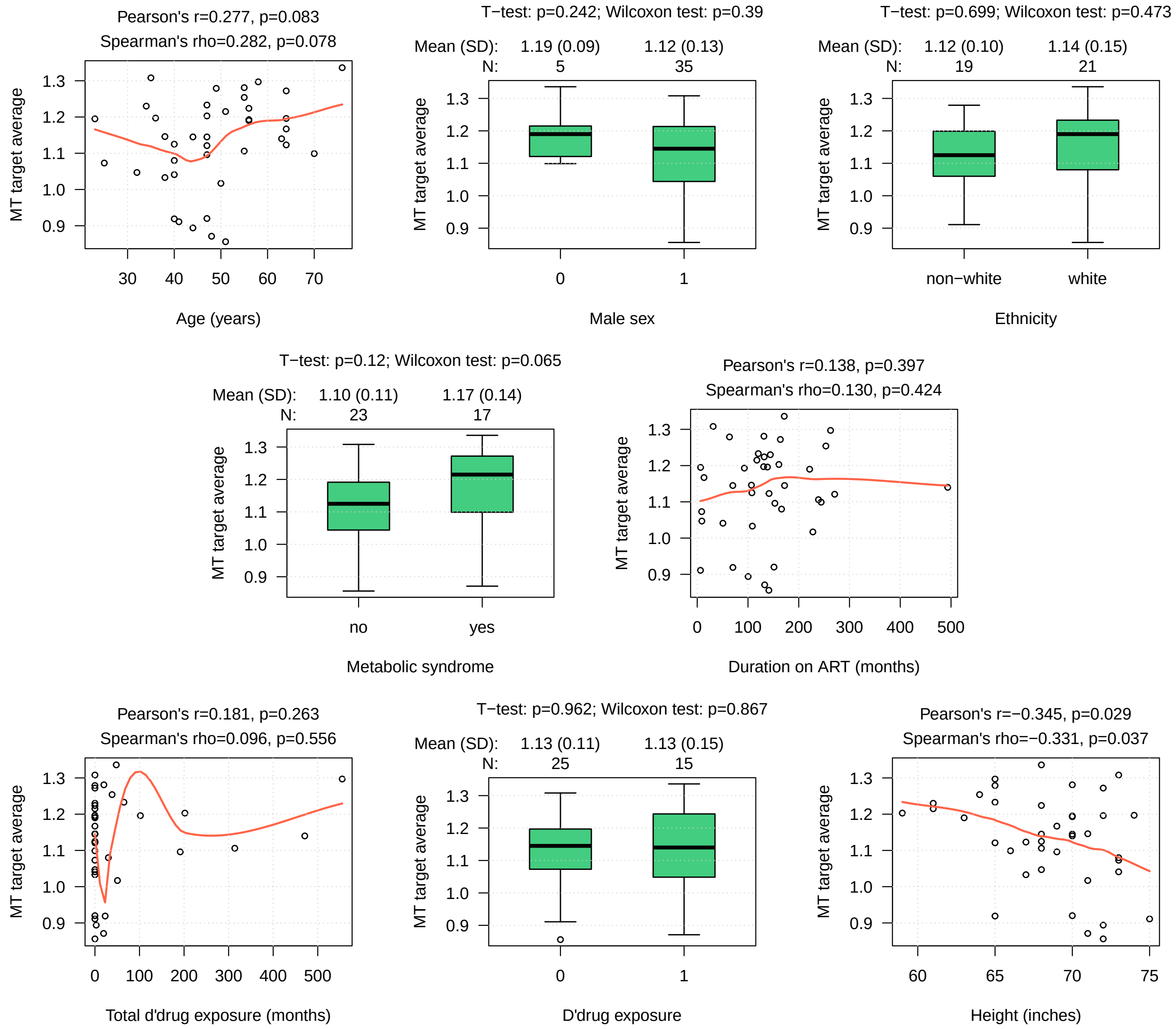
Pearson's r=0.277, p=0.083
Spearman's rho=0.282, p=0.078
MT target average
Age (years)
T−test: p=0.242; Wilcoxon test: p=0.39
Mean (SD): 1.19 (0.09) 1.12 (0.13)
N: 5 35
Male sex
T−test: p=0.699; Wilcoxon test: p=0.473
Mean (SD): 1.12 (0.10) 1.14 (0.15)
N: 19 21
non−white
white
Ethnicity
T−test: p=0.12; Wilcoxon test: p=0.065
Mean (SD): 1.10 (0.11) 1.17 (0.14)
N: 23 17
no
yes
Metabolic syndrome
Pearson's r=0.138, p=0.397
Spearman's rho=0.130, p=0.424
Duration on ART (months)
Pearson's r=0.181, p=0.263
Spearman's rho=0.096, p=0.556
Total d'drug exposure (months)
T−test: p=0.962; Wilcoxon test: p=0.867
Mean (SD): 1.13 (0.11) 1.13 (0.15)
N: 25 15
D'drug exposure
Pearson's r=−0.345, p=0.029
Spearman's rho=−0.331, p=0.037
Height (inches)

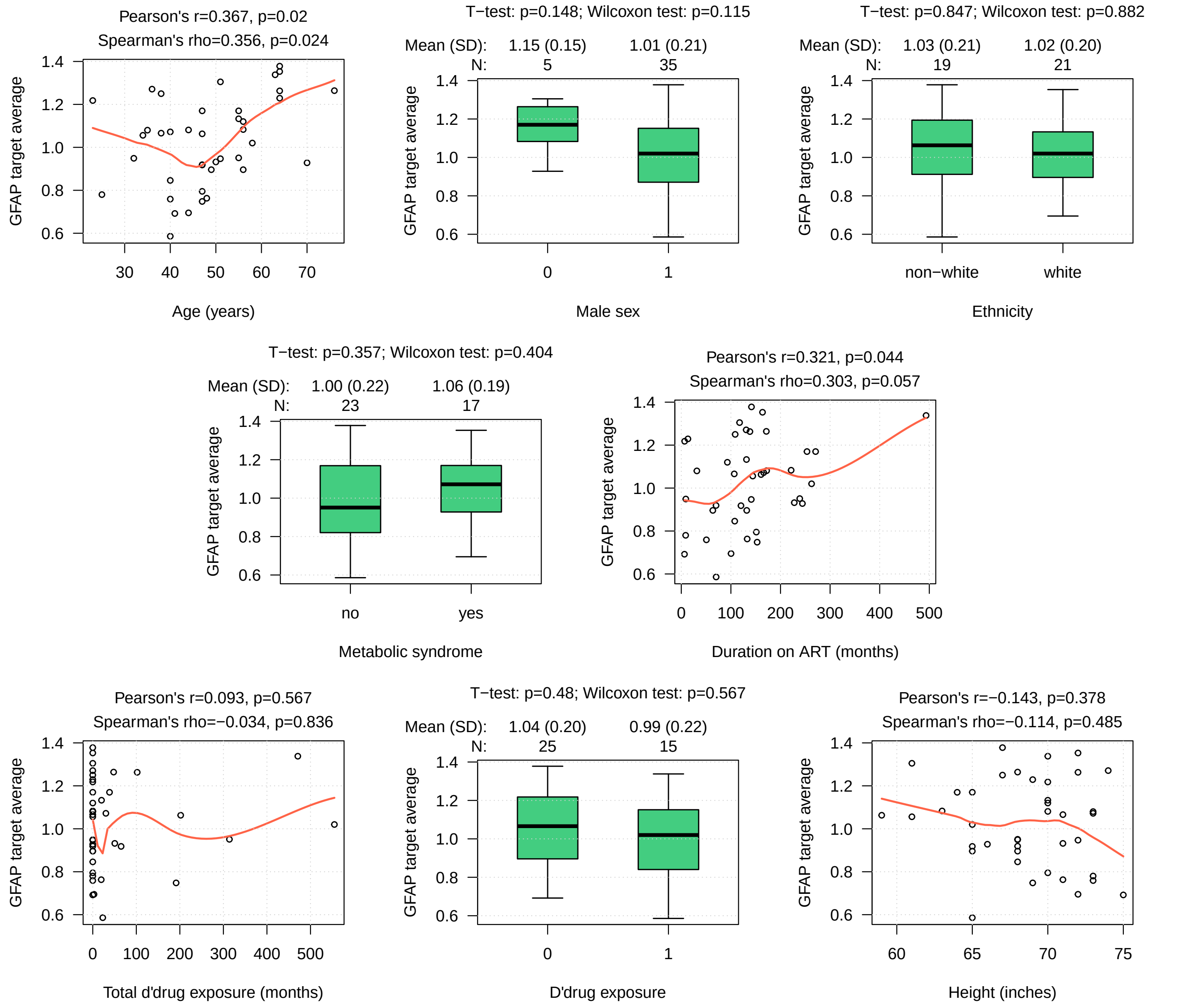
Pearson's r=0.367, p=0.02
Spearman's rho=0.356, p=0.024
GFAP target average
Age (years)
T−test: p=0.148; Wilcoxon test: p=0.115
Mean (SD): 1.15 (0.15) 1.01 (0.21)
N: 5 35
Male sex
T−test: p=0.847; Wilcoxon test: p=0.882
Mean (SD): 1.03 (0.21) 1.02 (0.20)
N: 19 21
non−white
white
Ethnicity
T−test: p=0.357; Wilcoxon test: p=0.404
Mean (SD): 1.00 (0.22) 1.06 (0.19)
N: 23 17
no
yes
Metabolic syndrome
Pearson's r=0.321, p=0.044
Spearman's rho=0.303, p=0.057
Duration on ART (months)
Pearson's r=0.093, p=0.567
Spearman's rho=−0.034, p=0.836
Total d'drug exposure (months)
T−test: p=0.48; Wilcoxon test: p=0.567
Mean (SD): 1.04 (0.20) 0.99 (0.22)
N: 25 15
D'drug exposure
Pearson's r=−0.143, p=0.378
Spearman's rho=−0.114, p=0.485
Height (inches)

2.3 Summary of univariable analyses

2.3.1 Simple correlations between outcomes and predictors

Learning and Motor T-scores were investigated for effects of outliers (71.5 & 7, respectively). Learning T-score did not have significant associations with predictors when the outlier was removed. Motor T-score did not have significant associations with predictors with or without the outlier.

MT spot average area has non-significant, but consistently negative correlation of moderate effect size with BDI-II total and subscores. It has positive associations with T-scores, approaching significant p-values for Global Mean T-score and Executive Functioning T-score and reaching significance for *Working Memory T-score.* Its associations with medical outcomes are not statistically significant.

MT spot average intensity has significant negative associations with BDI-II measures, specifically *BDI-II Total*, *Apathy*, *Somatic*, and *Affective* subscores. Statistically significant positive associations are found with *Global Mean T-score* and 3 domain scores: *Executive Function*, *SIP*, and *Working Memory.* Positive and marginally-significant association with cholesterol was also found.

MT spot total area/object shows no associations with any of the outcomes, including Learning T-score, which yielded statistically non-significant association after an outlier was removed ($r = -0.18$, $p = 0.274$).

MT target average has marginally significant negative correlations of moderate effect size with BDI-II total score and its subscores. Correlation tests with T-scores did not yield significant results, with most interesting association being positive correlation with Executive Function T-score. It does not correlate with medical outcomes.

GFAP target average does not correlate with BDI-II scores, but has negative association with cognitive T-scores, with marginal significance for Verbal and Recall domains. Statistical significance for Learning domain did not hold after removal of the outlier ($r = -0.261$, $p = 0.114$). There were no associations with medical outcomes.

2.3.2 Associations between outcomes and covariates

Covariates with p-values below 0.20 level are considered for inclusion into multivariable models. The following summary focuses on results with p-values below 0.20.

Tests for non-linear effects of numeric covariates on outcomes did not yield significant results.

The effect of a large outlier (493 months) in ART duration was tested. Its removal did not lead to any significant associations between ART duration and outcomes. Only one p-value remains below 0.20 level: for association with triglycerides.

There is no association between covariates and ***BDI-II*** total score or its subscores. Affective domain score's parametric association with height is driven by the data minimum in height. Its removal resulted in p-value above 0.20 ($r = 0.16$, $p = 0.345$), making parametric and nonparametric tests more consistent with each other.

For ***cognitive scores***, there is no covariates' effect on Global Mean T-score, as well as on domain scores in Verbal, Learning, and Recall. At 0.20 level, Executive Function and Working Memory T-scores are higher for those exposed to D'drugs; SIP T-score is, on average, higher for females; Motor T-score is, on average, lower for those with metabolic syndrome.

There is no statistical association between total cholesterol and any of the covariates at 0.20 level. Higher triglycerides levels were associated with non-white ethnicity, metabolic syndrome, longer duration of ART, and shorter height.

2.3.3 Associations between predictors and covariates

Covariates with p-values below 0.20 level are considered for inclusion into multivariable models. The following summary focuses on results with p-values below 0.20.

Tests for non-linear effects of numeric covariates on predictors did not yield significant results.

The effect of a large outlier (493 months) in ART duration was tested. Its removal did not change significance of associations between ART duration and predictors, except *GFAP target average*, where $r = 0.221$ ($p = 0.177$) without the outlier. Correlation with *MT spot total area/object* is $r = 0.300$ ($p = 0.064$) after the outlier removal.

Covariates are not statistically associated with *MT spot average area.*

At 0.20 significance level, higher *MT spot average intensity* is associated with female sex, metabolic syndrome, and shorter height.

At 0.20 significance level, higher levels of *MT spot total area/object* are associated with older age, longer duration on ART, and shorter height. Non-parametric tests also show association with ethnicity and metabolic syndrome. Although the multivariable model uses parametric tests, these 2 predictors will be considered for inclusion into multivariable models.

At 0.20 significance level, higher levels of *MT target average* are associated with older age, metabolic syndrome, and shorter height.

At 0.20 significance level, higher levels of *GFAP target average* are associated with older age, female sex, and longer ART duration.

3 Multivariable models

For multivariable models, two approaches are taken, because the sample size is small and a model with at most 4 predictors is recommended for n=40.

First approach investigates effect of a single covariate, regardless of its significance from univariable analyses, on each outcome-predictor association. This approach shows which, if any, of the covariates change either the significance, or the effect's magnitude, or the direction of the effect of the predictor on the outcome.

The second approach uses a backward model selection. For each outcome-predictor pair, the starting model regresses outcome on the predictor and those covariates that are significant at 0.20 level with either the outcome or the predictor. Stepwise backward model selection is implemented to reduce each model to those covariates that remain statistically significant at $\alpha = 0.20$ level. This could result in a model with no covariates or a model already shown in the first approach.

Outliers that were found to be influential (e.g., longest ART duration) in univariable analyses were removed for multivariable analyses. This leads to sample sizes below n=40 for some models.

As shown in the results below, the effects of the predictors on triglycerides seems to be influenced by covariates as noted by large changes in the predictors' coefficients and R-squared values. However, none of the models for predicting triglycerides yield a significant p-value for any predictor.

3.1 Controling for one covariate.

The following tables show results of analyses, where each outcome was regressed on predictor with adjustment for one covariate. Results are grouped by predictor. For each predictor, 2 tables are shown.

The first table shows model coefficients (slopes) and p-values for the effect of the predictor on the outcomes. The first column of such table shows unadjusted results for comparison.

The second table shows effect size for each model, in a form of partial R-squared. The first column shows unadjusted R-squared for comparison. R-squared measures percent of variability in the outcome explained by the predictor. Partial R-squared estimates association between the outcome and predictor with the effect of covariate removed.

Table 4: Coefficients (p-values) for effect of **MT spot average area** on outcomes (rows) alone and with control for a covariate (columns). Covariates' effects are not shown.

	No covariates	Age (years)	Male sex	Ethnicity	Metabolic syndrome	Duration on ART (months)	Height (inches)	D'drug exposure
BDI-II	-32.0 (0.123)	-31.7 (0.131)	-33.1 (0.116)	-31.9 (0.129)	-30.6 (0.146)	-33.0 (0.120)	-31.1 (0.135)	-32.6 (0.124)
BDI-II Cognitive	-9.2 (0.313)	-9.0 (0.327)	-9.6 (0.295)	-9.1 (0.321)	-8.4 (0.359)	-9.7 (0.299)	-8.9 (0.333)	-9.9 (0.281)
BDI-II Apathy	-7.8 (0.136)	-7.8 (0.141)	-8.1 (0.127)	-7.8 (0.143)	-7.6 (0.155)	-8.2 (0.122)	-7.5 (0.149)	-7.6 (0.155)
BDI-II Somatic	-17.0 (0.061)	-17.0 (0.064)	-17.5 (0.057)	-17.0 (0.065)	-16.6 (0.071)	-17.3 (0.062)	-16.7 (0.067)	-16.9 (0.068)
BDI-II Affective	-5.8 (0.206)	-5.8 (0.215)	-5.9 (0.206)	-5.8 (0.215)	-5.5 (0.237)	-6.1 (0.198)	-5.5 (0.225)	-5.8 (0.218)
Global Mean T	17.7 (0.101)	17.7 (0.106)	17.1 (0.118)	17.7 (0.105)	18.1 (0.101)	18.4 (0.098)	17.5 (0.110)	18.1 (0.099)
Verbal T	15.4 (0.319)	15.7 (0.318)	16.7 (0.285)	15.4 (0.325)	17.5 (0.262)	16.0 (0.313)	16.7 (0.280)	14.8 (0.347)
Executive T	24.6 (0.073)	24.7 (0.077)	23.3 (0.091)	24.6 (0.076)	22.7 (0.099)	25.4 (0.072)	24.5 (0.078)	26.8 (0.047)
SIP T	17.9 (0.194)	17.6 (0.209)	15.7 (0.247)	17.9 (0.197)	17.9 (0.203)	19.5 (0.162)	17.3 (0.215)	17.8 (0.204)
Learning T	8.9 (0.499)	9.2 (0.492)	8.8 (0.510)	9.3 (0.475)	10.6 (0.429)	9.6 (0.463)	8.4 (0.526)	8.4 (0.529)
Recall T	17.8 (0.202)	18.1 (0.203)	17.5 (0.218)	17.8 (0.203)	18.7 (0.189)	18.3 (0.196)	17.0 (0.227)	16.9 (0.231)
Working Memory T	36.1 (0.022)	35.5 (0.027)	35.1 (0.028)	36.1 (0.024)	34.8 (0.030)	35.3 (0.030)	35.6 (0.026)	38.6 (0.014)
Motor T	-7.5 (0.663)	-7.1 (0.684)	-7.0 (0.691)	-7.2 (0.680)	-5.3 (0.762)	-7.0 (0.693)	-7.6 (0.664)	-7.0 (0.690)
Total Cholesterol (mg/dL)	50.3 (0.490)	49.8 (0.500)	47.8 (0.519)	49.6 (0.501)	49.5 (0.506)	42.1 (0.573)	48.3 (0.512)	51.7 (0.487)
Triglycerides (mg/dL)	-83.8 (0.669)	-87.6 (0.658)	-93.9 (0.637)	-91.7 (0.627)	-129.1 (0.495)	-70.9 (0.718)	-104.3 (0.576)	-73.3 (0.713)

Table 5: R-squared (first column) and partial R-squared (remaining columns) for effect of **MT spot average area** on outcomes (rows) alone and with control for a covariate (columns). Covariates' effects are not shown.

	No covariates	Age (years)	Male sex	Ethnicity	Metabolic syndrome	Duration on ART (months)	Height (inches)	D'drug exposure
BDI-II	0.061	0.061	0.065	0.061	0.056	0.066	0.059	0.063
BDI-II Cognitive	0.027	0.026	0.030	0.027	0.023	0.030	0.025	0.031
BDI-II Apathy	0.058	0.058	0.062	0.057	0.054	0.065	0.055	0.054
BDI-II Somatic	0.090	0.089	0.095	0.089	0.085	0.094	0.088	0.087
BDI-II Affective	0.042	0.041	0.043	0.041	0.038	0.046	0.040	0.041
Global Mean T	0.071	0.071	0.066	0.071	0.073	0.076	0.069	0.074
Verbal T	0.027	0.028	0.032	0.027	0.035	0.029	0.032	0.025
Executive T	0.084	0.084	0.077	0.085	0.074	0.089	0.084	0.105
SIP T	0.045	0.044	0.037	0.046	0.045	0.055	0.042	0.045
Learning T	0.013	0.014	0.013	0.015	0.018	0.016	0.012	0.011
Recall T	0.044	0.045	0.042	0.045	0.047	0.047	0.040	0.040
Working Memory T	0.133	0.129	0.127	0.133	0.124	0.128	0.130	0.158
Motor T	0.005	0.005	0.005	0.005	0.003	0.005	0.005	0.005
Total Cholesterol (mg/dL)	0.013	0.013	0.012	0.013	0.012	0.009	0.012	0.014
Triglycerides (mg/dL)	0.005	0.006	0.006	0.007	0.013	0.004	0.009	0.004

In general, controlling for covariates does not change effect of MT spot average area on the considered outcomes.

The effect on Executive Functioning T-score strengthens and becomes statistically significant at 0.05 level when controlling for D'drug exposure.

Table 6: Coefficients (p-values) for effect of **MT spot average intensity** on outcomes (rows) alone and with control for a covariate (columns). Covariates' effects are not shown.

	No covariates	Age (years)	Male sex	Ethnicity	Metabolic syndrome	Duration on ART (months)	Height (inches)	D'drug exposure
BDI-II	-50.7 (0.034)	-50.7 (0.040)	-57.3 (0.022)	-50.4 (0.038)	-49.0 (0.048)	-55.6 (0.023)	-48.1 (0.052)	-51.1 (0.035)
BDI-II Cognitive	-13.0 (0.220)	-12.0 (0.272)	-15.3 (0.165)	-12.9 (0.230)	-11.6 (0.287)	-14.6 (0.181)	-11.9 (0.279)	-13.5 (0.205)
BDI-II Apathy	-12.4 (0.041)	-12.9 (0.039)	-14.1 (0.026)	-12.2 (0.047)	-12.2 (0.052)	-14.0 (0.022)	-11.3 (0.071)	-12.2 (0.047)
BDI-II Somatic	-26.5 (0.011)	-27.4 (0.011)	-29.7 (0.006)	-26.5 (0.012)	-26.5 (0.014)	-28.8 (0.006)	-26.1 (0.016)	-26.4 (0.012)
BDI-II Affective	-11.2 (0.033)	-11.3 (0.039)	-12.2 (0.027)	-11.0 (0.038)	-10.9 (0.046)	-12.2 (0.024)	-10.1 (0.061)	-11.2 (0.037)
Global Mean T	30.1 (0.015)	30.7 (0.016)	29.8 (0.022)	29.8 (0.018)	31.6 (0.014)	31.3 (0.015)	30.5 (0.018)	30.7 (0.015)
Verbal T	17.8 (0.327)	18.8 (0.313)	22.7 (0.230)	18.5 (0.318)	22.1 (0.235)	19.7 (0.291)	22.9 (0.217)	17.1 (0.354)
Executive T	45.2 (0.004)	46.2 (0.004)	43.7 (0.007)	46.5 (0.003)	42.9 (0.007)	45.9 (0.004)	46.6 (0.004)	47.9 (0.002)
SIP T	43.1 (0.006)	43.2 (0.007)	38.9 (0.015)	42.4 (0.007)	44.6 (0.006)	44.3 (0.005)	42.7 (0.008)	43.3 (0.006)
Learning T	6.7 (0.670)	7.5 (0.641)	6.7 (0.684)	5.1 (0.741)	10.0 (0.541)	9.8 (0.530)	5.0 (0.755)	6.2 (0.697)
Recall T	13.2 (0.425)	13.8 (0.415)	12.5 (0.470)	11.9 (0.477)	14.9 (0.385)	15.5 (0.356)	10.6 (0.533)	12.1 (0.471)
Working Memory T	59.3 (0.001)	58.9 (0.001)	59.4 (0.002)	60.3 (0.001)	58.4 (0.002)	58.5 (0.002)	59.7 (0.001)	62.4 (0.000)
Motor T	-6.5 (0.756)	-5.5 (0.799)	-4.9 (0.823)	-7.4 (0.727)	-2.0 (0.928)	-6.0 (0.783)	-7.1 (0.743)	-6.2 (0.772)
Total Cholesterol (mg/dL)	131.1 (0.119)	132.5 (0.128)	130.8 (0.138)	129.2 (0.129)	134.5 (0.124)	132.3 (0.126)	126.6 (0.148)	132.6 (0.121)
Triglycerides (mg/dL)	-13.8 (0.952)	-45.5 (0.847)	-47.1 (0.845)	-41.4 (0.852)	-119.3 (0.597)	-59.4 (0.796)	-137.5 (0.540)	-4.5 (0.985)

Table 7: R-squared (first column) and partial R-squared (remaining columns) for effect of **MT spot average intensity** on outcomes (rows) alone and with control for a covariate (columns). Covariates' effects are not shown.

	No covariates	Age (years)	Male sex	Ethnicity	Metabolic syndrome	Duration on ART (months)	Height (inches)	D'drug exposure
BDI-II	0.113	0.109	0.135	0.111	0.102	0.136	0.098	0.114
BDI-II Cognitive	0.039	0.033	0.051	0.039	0.031	0.049	0.032	0.043
BDI-II Apathy	0.105	0.110	0.127	0.103	0.098	0.138	0.086	0.102
BDI-II Somatic	0.159	0.164	0.187	0.159	0.152	0.190	0.148	0.157
BDI-II Affective	0.114	0.110	0.126	0.111	0.103	0.134	0.091	0.113
Global Mean T	0.148	0.150	0.137	0.145	0.156	0.158	0.146	0.153
Verbal T	0.026	0.028	0.040	0.028	0.039	0.032	0.042	0.024
Executive T	0.206	0.210	0.185	0.217	0.185	0.210	0.210	0.242
SIP T	0.190	0.187	0.155	0.184	0.194	0.203	0.180	0.189
Learning T	0.005	0.006	0.005	0.003	0.011	0.012	0.003	0.004
Recall T	0.017	0.019	0.015	0.014	0.021	0.024	0.011	0.015
Working Memory T	0.259	0.252	0.247	0.266	0.246	0.252	0.253	0.298
Motor T	0.003	0.002	0.001	0.004	0.000	0.002	0.003	0.002
Total Cholesterol (mg/dL)	0.064	0.063	0.060	0.063	0.065	0.066	0.057	0.065
Triglycerides (mg/dL)	0.000	0.001	0.001	0.001	0.008	0.002	0.011	0.000

In general, controlling for covariates does not change effect of MT spot average intensity on the considered outcomes.

Table 8: Coefficients (p-values) for effect of **MT spot total area/object** on outcomes (rows) alone and with control for a covariate (columns). Covariates' effects are not shown.

	No covariates	Age (years)	Male sex	Ethnicity	Metabolic syndrome	Duration on ART (months)	Height (inches)	D'drug exposure
BDI-II	-4.6 (0.396)	-4.3 (0.457)	-4.9 (0.368)	-4.9 (0.374)	-3.9 (0.475)	-6.5 (0.259)	-3.6 (0.523)	-4.6 (0.403)
BDI-II Cognitive	-1.6 (0.492)	-1.2 (0.624)	-1.8 (0.456)	-1.7 (0.475)	-1.3 (0.587)	-2.2 (0.386)	-1.3 (0.600)	-1.6 (0.501)
BDI-II Apathy	-1.1 (0.438)	-1.2 (0.419)	-1.2 (0.407)	-1.2 (0.400)	-0.9 (0.499)	-1.7 (0.247)	-0.7 (0.619)	-1.1 (0.438)
BDI-II Somatic	-2.1 (0.379)	-2.2 (0.378)	-2.2 (0.353)	-2.2 (0.368)	-1.9 (0.437)	-3.1 (0.220)	-1.8 (0.475)	-2.1 (0.382)
BDI-II Affective	-0.9 (0.457)	-0.8 (0.518)	-0.9 (0.451)	-1.0 (0.406)	-0.8 (0.538)	-1.2 (0.338)	-0.5 (0.667)	-0.9 (0.461)
Global Mean T	0.3 (0.910)	0.3 (0.931)	0.1 (0.981)	0.5 (0.868)	0.4 (0.903)	0.4 (0.894)	0.1 (0.977)	0.3 (0.913)
Verbal T	-3.2 (0.414)	-3.3 (0.429)	-2.9 (0.467)	-3.4 (0.398)	-2.7 (0.504)	-2.7 (0.525)	-2.4 (0.562)	-3.2 (0.421)
Executive T	3.2 (0.368)	3.5 (0.360)	2.8 (0.440)	3.0 (0.403)	2.5 (0.487)	3.0 (0.431)	3.2 (0.385)	3.2 (0.366)
SIP T	3.1 (0.379)	3.0 (0.430)	2.4 (0.490)	3.5 (0.330)	3.1 (0.393)	2.4 (0.516)	2.7 (0.466)	3.1 (0.385)
Learning T	-3.8 (0.274)	-4.0 (0.291)	-3.9 (0.272)	-3.0 (0.394)	-3.5 (0.346)	-2.4 (0.519)	-4.5 (0.211)	-3.7 (0.299)
Recall T	-3.1 (0.381)	-3.3 (0.382)	-3.4 (0.358)	-2.8 (0.442)	-3.0 (0.409)	-2.1 (0.584)	-4.2 (0.261)	-3.1 (0.386)
Working Memory T	2.0 (0.639)	1.3 (0.773)	1.6 (0.713)	1.9 (0.660)	1.4 (0.751)	1.8 (0.679)	1.5 (0.735)	1.9 (0.642)
Motor T	-3.0 (0.524)	-2.7 (0.591)	-2.8 (0.554)	-2.4 (0.618)	-2.1 (0.661)	-3.3 (0.509)	-3.2 (0.508)	-3.2 (0.497)
Total Cholesterol (mg/dL)	-2.4 (0.898)	-4.3 (0.831)	-3.5 (0.853)	-1.3 (0.945)	-3.2 (0.870)	1.0 (0.961)	-5.5 (0.781)	-2.4 (0.898)
Triglycerides (mg/dL)	28.0 (0.579)	19.7 (0.713)	25.0 (0.626)	41.3 (0.398)	8.8 (0.859)	2.0 (0.970)	1.3 (0.979)	27.4 (0.591)

Table 9: R-squared (first column) and partial R-squared (remaining columns) for effect of **MT spot total area/object** on outcomes (rows) alone and with control for a covariate (columns). Covariates' effects are not shown.

	No covariates	Age (years)	Male sex	Ethnicity	Metabolic syndrome	Duration on ART (months)	Height (inches)	D'drug exposure
BDI-II	0.019	0.015	0.022	0.021	0.014	0.035	0.011	0.019
BDI-II Cognitive	0.013	0.007	0.015	0.014	0.008	0.021	0.008	0.012
BDI-II Apathy	0.016	0.018	0.019	0.019	0.012	0.037	0.007	0.016
BDI-II Somatic	0.020	0.021	0.023	0.022	0.016	0.041	0.014	0.021
BDI-II Affective	0.015	0.011	0.015	0.019	0.010	0.026	0.005	0.015
Global Mean T	0.000	0.000	0.000	0.001	0.000	0.001	0.000	0.000
Verbal T	0.018	0.017	0.015	0.020	0.013	0.012	0.009	0.018
Executive T	0.022	0.023	0.017	0.019	0.014	0.018	0.021	0.023
SIP T	0.021	0.017	0.013	0.026	0.020	0.012	0.015	0.021
Learning T	0.033	0.032	0.034	0.021	0.025	0.012	0.044	0.031
Recall T	0.021	0.021	0.023	0.017	0.019	0.009	0.035	0.021
Working Memory T	0.006	0.002	0.004	0.005	0.003	0.005	0.003	0.006
Motor T	0.011	0.008	0.010	0.007	0.006	0.013	0.013	0.013
Total Cholesterol (mg/dL)	0.000	0.001	0.001	0.000	0.001	0.000	0.002	0.000
Triglycerides (mg/dL)	0.008	0.004	0.007	0.020	0.001	0.000	0.000	0.008

Overall, there were no statistically significant results for models with MT spot total area/object as a predictor. The size effect, in a form of R-squared, remained fairly small (below 0.045 or 4.5%) for all models.

Table 10: Coefficients (p-values) for effect of **MT target average** on outcomes (rows) alone and with control for a covariate (columns). Covariates' effects are not shown.

	No covariates	Age (years)	Male sex	Ethnicity	Metabolic syndrome	Duration on ART (months)	Height (inches)	D'drug exposure
BDI-II	-30.2 (0.057)	-30.6 (0.067)	-32.5 (0.045)	-30.6 (0.057)	-28.9 (0.080)	-32.9 (0.043)	-28.5 (0.093)	-30.2 (0.060)
BDI-II Cognitive	-11.0 (0.110)	-10.5 (0.148)	-12.1 (0.089)	-11.2 (0.111)	-10.3 (0.153)	-11.8 (0.097)	-10.6 (0.153)	-11.1 (0.112)
BDI-II Apathy	-7.1 (0.077)	-7.7 (0.069)	-7.7 (0.062)	-7.2 (0.075)	-7.0 (0.097)	-7.9 (0.051)	-6.2 (0.149)	-7.1 (0.081)
BDI-II Somatic	-12.9 (0.064)	-13.8 (0.061)	-14.0 (0.052)	-13.1 (0.065)	-12.7 (0.081)	-14.4 (0.044)	-12.7 (0.092)	-12.9 (0.068)
BDI-II Affective	-6.2 (0.077)	-6.3 (0.088)	-6.5 (0.071)	-6.4 (0.072)	-5.9 (0.104)	-6.7 (0.064)	-5.2 (0.161)	-6.2 (0.081)
Global Mean T	9.4 (0.261)	9.9 (0.260)	8.6 (0.316)	9.6 (0.256)	10.1 (0.247)	9.6 (0.268)	9.5 (0.291)	9.4 (0.266)
Verbal T	5.6 (0.638)	6.6 (0.596)	7.4 (0.544)	5.4 (0.652)	8.6 (0.484)	6.8 (0.579)	11.0 (0.381)	5.5 (0.648)
Executive T	18.0 (0.086)	19.2 (0.082)	16.5 (0.123)	17.8 (0.094)	15.8 (0.144)	17.7 (0.105)	19.5 (0.084)	18.3 (0.078)
SIP T	19.2 (0.065)	19.6 (0.074)	16.6 (0.110)	19.7 (0.060)	20.0 (0.065)	18.1 (0.089)	19.0 (0.090)	19.2 (0.069)
Learning T	-3.7 (0.729)	-3.2 (0.777)	-4.0 (0.714)	-2.1 (0.839)	-1.5 (0.892)	-1.4 (0.894)	-6.4 (0.570)	-3.3 (0.754)
Recall T	-3.8 (0.722)	-3.8 (0.738)	-4.7 (0.672)	-3.4 (0.756)	-3.2 (0.773)	-2.1 (0.848)	-7.9 (0.490)	-4.0 (0.714)
Working Memory T	16.9 (0.171)	16.0 (0.217)	15.6 (0.217)	16.8 (0.179)	15.2 (0.236)	17.1 (0.180)	16.4 (0.216)	17.2 (0.160)
Motor T	-7.2 (0.596)	-6.3 (0.660)	-6.5 (0.640)	-6.3 (0.648)	-4.3 (0.768)	-7.5 (0.594)	-8.4 (0.561)	-7.5 (0.582)
Total Cholesterol (mg/dL)	45.9 (0.412)	45.7 (0.439)	43.0 (0.456)	47.6 (0.401)	46.5 (0.428)	53.7 (0.351)	39.5 (0.512)	45.9 (0.418)
Triglycerides (mg/dL)	146.5 (0.329)	129.8 (0.411)	137.2 (0.375)	164.4 (0.255)	76.4 (0.610)	109.3 (0.470)	41.7 (0.785)	146.9 (0.333)

Table 11: R-squared (first column) and partial R-squared (remaining columns) for effect of **MT target average** on outcomes (rows) alone and with control for a covariate (columns). Covariates' effects are not shown.

	No covariates	Age (years)	Male sex	Ethnicity	Metabolic syndrome	Duration on ART (months)	Height (inches)	D'drug exposure
BDI-II	0.092	0.088	0.104	0.095	0.081	0.109	0.074	0.092
BDI-II Cognitive	0.066	0.056	0.076	0.067	0.054	0.075	0.054	0.067
BDI-II Apathy	0.080	0.087	0.091	0.083	0.073	0.102	0.055	0.080
BDI-II Somatic	0.087	0.092	0.099	0.089	0.080	0.108	0.075	0.087
BDI-II Affective	0.080	0.077	0.085	0.085	0.070	0.092	0.052	0.080
Global Mean T	0.034	0.035	0.028	0.036	0.037	0.035	0.031	0.034
Verbal T	0.006	0.008	0.010	0.006	0.014	0.009	0.021	0.006
Executive T	0.077	0.082	0.065	0.076	0.058	0.073	0.081	0.084
SIP T	0.089	0.086	0.069	0.094	0.091	0.080	0.078	0.089
Learning T	0.003	0.002	0.004	0.001	0.001	0.001	0.009	0.003
Recall T	0.003	0.003	0.005	0.003	0.002	0.001	0.013	0.004
Working Memory T	0.050	0.042	0.042	0.050	0.039	0.051	0.042	0.054
Motor T	0.008	0.006	0.006	0.006	0.003	0.008	0.010	0.009
Total Cholesterol (mg/dL)	0.018	0.017	0.016	0.020	0.018	0.025	0.012	0.018
Triglycerides (mg/dL)	0.026	0.019	0.022	0.036	0.007	0.015	0.002	0.026

For MT target average, models adjusted for covariates show results consistent with results of univariable analyses.

P-values for predicting BDI-II Total and Somatic from this predictor become statistically significant with 0.05 level in presence of some covariates. Note that, when model selection was applied for these outcomes (see section 3.2), none of the covariates remained in the models.

Table 12: Coefficients (p-values) for effect of **GFAP target average** on outcomes (rows) alone and with control for a covariate (columns). Covariates' effects are not shown.

	No covariates	Age (years)	Male sex	Ethnicity	Metabolic syndrome	Duration on ART (months)	Height (inches)	D'drug exposure
BDI-II	-3.1 (0.753)	-1.8 (0.867)	-4.3 (0.680)	-3.0 (0.763)	-2.1 (0.839)	-7.0 (0.516)	-1.9 (0.852)	-3.3 (0.749)
BDI-II Cognitive	-1.1 (0.792)	-0.1 (0.991)	-1.7 (0.708)	-1.1 (0.800)	-0.6 (0.885)	-2.4 (0.603)	-0.7 (0.870)	-1.5 (0.740)
BDI-II Apathy	-0.8 (0.757)	-0.9 (0.741)	-1.1 (0.678)	-0.7 (0.770)	-0.6 (0.820)	-2.1 (0.431)	-0.4 (0.885)	-0.6 (0.807)
BDI-II Somatic	-0.7 (0.878)	-0.6 (0.901)	-1.1 (0.804)	-0.7 (0.884)	-0.3 (0.946)	-2.4 (0.611)	-0.2 (0.959)	-0.5 (0.909)
BDI-II Affective	-1.3 (0.546)	-1.2 (0.623)	-1.5 (0.523)	-1.3 (0.562)	-1.1 (0.617)	-2.1 (0.365)	-0.9 (0.671)	-1.3 (0.565)
Global Mean T	-2.3 (0.664)	-2.7 (0.627)	-3.3 (0.547)	-2.4 (0.647)	-2.3 (0.671)	-2.0 (0.728)	-2.5 (0.638)	-2.2 (0.688)
Verbal T	-13.5 (0.064)	-14.6 (0.060)	-13.0 (0.085)	-13.4 (0.069)	-12.9 (0.081)	-13.1 (0.094)	-12.8 (0.081)	-14.3 (0.054)
Executive T	3.7 (0.582)	4.0 (0.580)	2.2 (0.747)	3.9 (0.564)	2.7 (0.688)	3.6 (0.614)	3.6 (0.600)	5.1 (0.444)
SIP T	4.9 (0.460)	4.6 (0.521)	2.6 (0.696)	4.6 (0.491)	4.9 (0.474)	5.1 (0.473)	4.5 (0.508)	4.9 (0.476)
Learning T	-9.9 (0.114)	-10.6 (0.117)	-10.6 (0.104)	-10.2 (0.098)	-9.5 (0.141)	-7.5 (0.254)	-10.4 (0.103)	-10.4 (0.102)
Recall T	-11.9 (0.071)	-13.1 (0.064)	-13.1 (0.055)	-12.4 (0.063)	-11.9 (0.079)	-10.6 (0.130)	-12.7 (0.056)	-12.9 (0.053)
Working Memory T	5.6 (0.476)	4.5 (0.593)	4.4 (0.586)	5.7 (0.473)	4.8 (0.545)	4.5 (0.589)	5.1 (0.517)	7.1 (0.359)
Motor T	-6.4 (0.443)	-6.0 (0.502)	-6.0 (0.486)	-6.6 (0.435)	-5.4 (0.529)	-6.6 (0.463)	-6.5 (0.444)	-6.1 (0.474)
Total Cholesterol (mg/dL)	-5.1 (0.883)	-9.4 (0.803)	-8.9 (0.805)	-5.4 (0.877)	-6.1 (0.864)	-6.0 (0.873)	-8.2 (0.817)	-4.8 (0.892)
Triglycerides (mg/dL)	104.1 (0.261)	95.1 (0.344)	98.7 (0.305)	100.7 (0.258)	80.3 (0.372)	69.5 (0.475)	77.0 (0.388)	111.7 (0.235)

Table 13: R-squared (first column) and partial R-squared (remaining columns) for effect of **GFAP target average** on outcomes (rows) alone and with control for a covariate (columns). Covariates' effects are not shown.

	No covariates	Age (years)	Male sex	Ethnicity	Metabolic syndrome	Duration on ART (months)	Height (inches)	D'drug exposure
BDI-II	0.003	0.001	0.005	0.002	0.001	0.012	0.001	0.003
BDI-II Cognitive	0.002	0.000	0.004	0.002	0.001	0.008	0.001	0.003
BDI-II Apathy	0.003	0.003	0.005	0.002	0.001	0.017	0.001	0.002
BDI-II Somatic	0.001	0.000	0.002	0.001	0.000	0.007	0.000	0.000
BDI-II Affective	0.010	0.007	0.011	0.009	0.007	0.023	0.005	0.009
Global Mean T	0.005	0.007	0.010	0.006	0.005	0.003	0.006	0.005
Verbal T	0.090	0.095	0.080	0.089	0.082	0.078	0.082	0.099
Executive T	0.008	0.009	0.003	0.009	0.005	0.007	0.008	0.016
SIP T	0.015	0.012	0.004	0.013	0.014	0.015	0.012	0.014
Learning T	0.068	0.069	0.074	0.076	0.061	0.038	0.074	0.075
Recall T	0.085	0.092	0.098	0.093	0.083	0.064	0.098	0.100
Working Memory T	0.014	0.008	0.008	0.014	0.010	0.008	0.012	0.023
Motor T	0.016	0.013	0.014	0.018	0.011	0.016	0.017	0.015
Total Cholesterol (mg/dL)	0.001	0.002	0.002	0.001	0.001	0.001	0.002	0.001
Triglycerides (mg/dL)	0.034	0.025	0.029	0.035	0.022	0.015	0.021	0.039

Overall, the multivariable models adjusting effect of GFAP target average for covariates yield results consistent with univariable analyses.

3.2 Controling for covariates significant at 0.20 level

Backward model selection with p < 0.20 as the criterion for covariates was used. The results are grouped by predictor and summarized below.

Predictor: **MT spot average area** No covariates were associated with this predictor at $\alpha = 0.20$, thus, no multivariable models were built for BDI-II total score and its subscores, for Global, Learning, Recall, and Verbal T-scores, and for total cholesterol, all of which also did not have significant associations with covariates at 0.20 level. Model regressing Motor T-score on this predictor did not retain covariates. Models for the remaining outcomes are summarized in tables below. Some of them have been shown previously in Tables 4 & 5.

Table 14: Multivariable model for effect of **MT spot average area** on **Executive T** (n=39; model R-squared=0.104).

	Model coefficient	P-value
MT spot average area	26.8	0.047
D'drug exposure	4.4	0.101

Table 15: Multivariable model for effect of **MT spot average area** on **SIP T** (n=39; model R-squared=0.056).

	Model coefficient	P-value
MT spot average area	15.7	0.247
Male sex	-6.0	0.127

Table 16: Multivariable model for effect of **MT spot average area** on **Working Memory T** (n=39; model R-squared=0.152).

	Model coefficient	P-value
MT spot average area	38.6	0.014
D'drug exposure	5.0	0.101

Table 17: Multivariable model for effect of **MT spot average area** on **Triglycerides (mg/dL)** (n=39; model R-squared=0.155).

	Model coefficient	P-value
MT spot average area	-138.7	0.442
White Ethnicity	-75.5	0.036
Metabolic syndrome	80.5	0.028

Predictor: **MT spot average intensity**

Sex, metabolic syndrome, and height were associated with this predictor at $\alpha = 0.20$, thus multivariable models were considered for all outcomes. Additional covariates were considered for some outcomes if their univariable association has p-value < 0.20. Models for BDI-II Total score and its subscores did not retain a covariate, as well as models for Global Mean T-score, SIP , Learning, Recall, and Motor T-scores, and total cholesterol. Models for the remaining outcomes are summarized in tables below. Some of them have been shown previously in Tables 6 & 7.

Table 18: Multivariable model for effect of **MT spot average intensity** on **Verbal T** (n=39; model R-squared=0.018).

	Model coefficient	P-value
MT spot average intensity	22.9	0.217
Height (inches)	0.5	0.200

Table 19: Multivariable model for effect of **MT spot average intensity** on **Executive T** (n=39; model R-squared=0.241).

	Model coefficient	P-value
MT spot average intensity	47.9	0.002
D'drug exposure	4.6	0.061

Table 20: Multivariable model for effect of **MT spot average intensity** on **Working Memory T** (n=39; model R-squared=0.293).

	Model coefficient	P-value
MT spot average intensity	62.4	0.000
D'drug exposure	5.2	0.060

Table 21: Multivariable model for effect of **MT spot average intensity** on **Triglycerides (mg/dL)** (n=39; model R-squared=0.176).

	Model coefficient	P-value
MT spot average intensity	-211.1	0.331
White Ethnicity	-57.1	0.131
Metabolic syndrome	74.3	0.046
Height (inches)	-7.1	0.168

Predictor: **MT spot total area/object**

Age, ART duration, ethnicity, metabolic syndrome, and height were considered as covariates for this predictor. Multivariable models were considered for all outcomes. Additional covariates were considered for some outcomes if their univariable association has p-value < 0.20. The models for the following outcomes did not retain covariates that would be significant at 0.20 level: BDI-II Total score, Cognitive, Somatic, and Affective subscores, Global Mean T-score, Verbal, Learning, Recall, Working Memory, and Motor T-scores, and total cholesterol. Models for the remaining outcomes are summarized in tables below. Some of them have been shown previously in Tables 8 & 9.

Table 22: Multivariable model for effect of **MT spot total area/object** on **BDI-II Apathy** (n=39; model R-squared=0.027).

	Model coefficient	P-value
MT spot total area/object	-1.3	0.352
Duration on ART (months)	0.0	0.132
Height (inches)	0.2	0.174

Table 23: Multivariable model for effect of **MT spot total area/object** on **Executive T** (n=39; model R-squared=0.022).

	Model coefficient	P-value
MT spot total area/object	3.2	0.366
D'drug exposure	3.8	0.166

Table 24: Multivariable model for effect of **MT spot total area/object** on **SIP T** (n=39; model R-squared=0.033).

	Model coefficient	P-value
MT spot total area/object	2.4	0.490
Male sex	-6.2	0.125

Table 25: Multivariable model for effect of **MT spot total area/object** on **Triglycerides (mg/dL)** (n=39; model R-squared=0.146).

	Model coefficient	P-value
MT spot total area/object	22.1	0.642
White Ethnicity	-77.0	0.035
Metabolic syndrome	74.1	0.046

Predictor: **MT target average**

Age, metabolic syndrome, and height were considered as covariates for this predictor. Multivariable models were considered for all outcomes. Additional covariates were considered for some outcomes if their univariable association has p-value < 0.20. The models for the following outcomes did not retain covariates that would be significant at 0.20 level: BDI-II Total score and all subscores, Global Mean T-score, Verbal, Learning, Recall, and Motor T-scores, and total cholesterol. Models for the remaining outcomes are summarized in tables below. Some of them have been shown previously in Tables 10 & 11.

Table 26: Multivariable model for effect of **MT target average** on **Executive T** (n=39; model R-squared=0.083).

	Model coefficient	P-value
MT target average	18.3	0.078
D'drug exposure	3.9	0.144

Table 27: Multivariable model for effect of **MT target average** on **SIP T** (n=39; model R-squared=0.088).

	Model coefficient	P-value
MT target average	16.6	0.110
Male sex	-5.3	0.173

Table 28: Multivariable model for effect of **MT target average** on **Working Memory T** (n=39; model R-squared=0.047).

	Model coefficient	P-value
MT target average	17.2	0.160
D'drug exposure	4.3	0.178

Table 29: Multivariable model for effect of **MT target average** on **Triglycerides (mg/dL)** (n=39; model R-squared=0.151).

	Model coefficient	P-value
MT target average	93.5	0.513
White Ethnicity	-76.1	0.035
Metabolic syndrome	71.3	0.056

Predictor: **GFAP target average**

Age, sex and ART duration were significant ($\alpha = 0.20$) covariates for this predictor. Multivariable models were considered for all outcomes. Additional covariates were considered for some outcomes if their univariable association has p-value < 0.20. The models for the following outcomes did not retain covariates that would be significant at 0.20 level: BDI-II Total score and all its subscores, Global Mean T-score, Verbal, Learning, Recall, and Motor T-scores, and total cholesterol. Models for the remaining outcomes are summarized in tables below. Some of them have been shown previously in Tables 10 & 11.

Table 30: Multivariable model for effect of **GFAP target average** on **Executive T** (n=39; model R-squared=0.015).

	Model coefficient	P-value
GFAP target average	5.1	0.444
D'drug exposure	4.1	0.140

Table 31: Multivariable model for effect of **GFAP target average** on **SIP T** (n=39; model R-squared=0.024).

	Model coefficient	P-value
GFAP target average	2.6	0.696
Male sex	-6.2	0.133

Table 32: Multivariable model for effect of **GFAP target average** on **Working Memory T** (n=39; model R-squared=0.016).

	Model coefficient	P-value
GFAP target average	7.1	0.359
D'drug exposure	4.6	0.157

Table 33: Multivariable model for effect of **GFAP target average** on **Triglycerides (mg/dL)** (n=39; model R-squared=0.159).

	Model coefficient	P-value
GFAP target average	76.0	0.376
White Ethnicity	-74.1	0.039
Metabolic syndrome	72.9	0.045
